# Unimolecular Reactions of *E*-Glycolaldehyde Oxide and Its Reactions with One and Two Water Molecules

**DOI:** 10.34133/research.0143

**Published:** 2023-07-10

**Authors:** Yan Sun, Bo Long, Donald G. Truhlar

**Affiliations:** ^1^ Department of Physics, Guizhou University, Guiyang 550025, China.; ^2^ College of Materials Science and Engineering, Guizhou Minzu University, Guiyang 550025, China.; ^3^ Department of Chemistry, Chemical Theory Center, and Supercomputing Institute, University of Minnesota, Minneapolis, MN 55455-0431, USA.

## Abstract

The kinetics of Criegee intermediates are important for atmospheric modeling. However, the quantitative kinetics of Criegee intermediates are still very limited, especially for those with hydroxy groups. Here, we calculate rate constants for the unimolecular reaction of *E*-glycolaldehyde oxide [*E*-hydroxyethanal oxide, *E*-(CH_2_OH)CHOO], for its reactions with H_2_O and (H_2_O)_2_, and for the reaction of the *E*-(CH_2_OH)CHOO…H_2_O complex with H_2_O. For the highest level of electronic structure, we use W3X-L//CCSD(T)-F12a/cc-pVDZ-F12 for the unimolecular reaction and the reaction with water and W3X-L//DF-CCSD(T)-F12b/jun-cc-pVDZ for the reaction with 2 water molecules. For the dynamics, we use a dual-level strategy that combines conventional transition state theory with the highest level of electronic structure and multistructural canonical variational transition state theory with small-curvature tunneling with a validated density functional for the electronic structure. This dynamical treatment includes high-frequency anharmonicity, torsional anharmonicity, recrossing effects, and tunneling. We find that the unimolecular reaction of *E*-(CH_2_OH)CHOO depends on both temperature and pressure. The calculated results show that *E*-(CH_2_OH)CHOO…H_2_O + H_2_O is the dominant entrance channel, while previous investigations only considered Criegee intermediates + (H_2_O)_2_. In addition, we find that the atmospheric lifetime of *E*-(CH_2_OH)CHOO with respect to 2 water molecules is particularly short with a value of 1.71 × 10^−6^ s at 0 km, which is about 2 orders of magnitude shorter than those usually assumed for Criegee intermediate reactions with water dimer. We also find that the OH group in *E*-(CH_2_OH)CHOO enhances its reactivity.

## Introduction

Criegee intermediates [1] are key atmospheric species derived from the ozonolysis of volatile unsaturated organic compounds in the atmosphere [2–4]. Their unimolecular reactions are often considered to be the main nonphotolytic source of the OH cleanser radical during the night [4–14], although atmospheric OH formation from photolytic sources significantly exceeds that from ozonolysis in general. Criegee intermediates also make important contributions as sinks for other atmospheric species [3,15–22], and they significantly influence the budget of sulfuric acid [19,23–28], the growth of secondary organic aerosols [29–34], and the production of some highly oxygenated products [35–38]. The rate constants of Criegee intermediates are key parameters for elucidating these chemical transformations; however, experimental knowledge of these rate constants is limited [39–41].

**Fig. 1.**
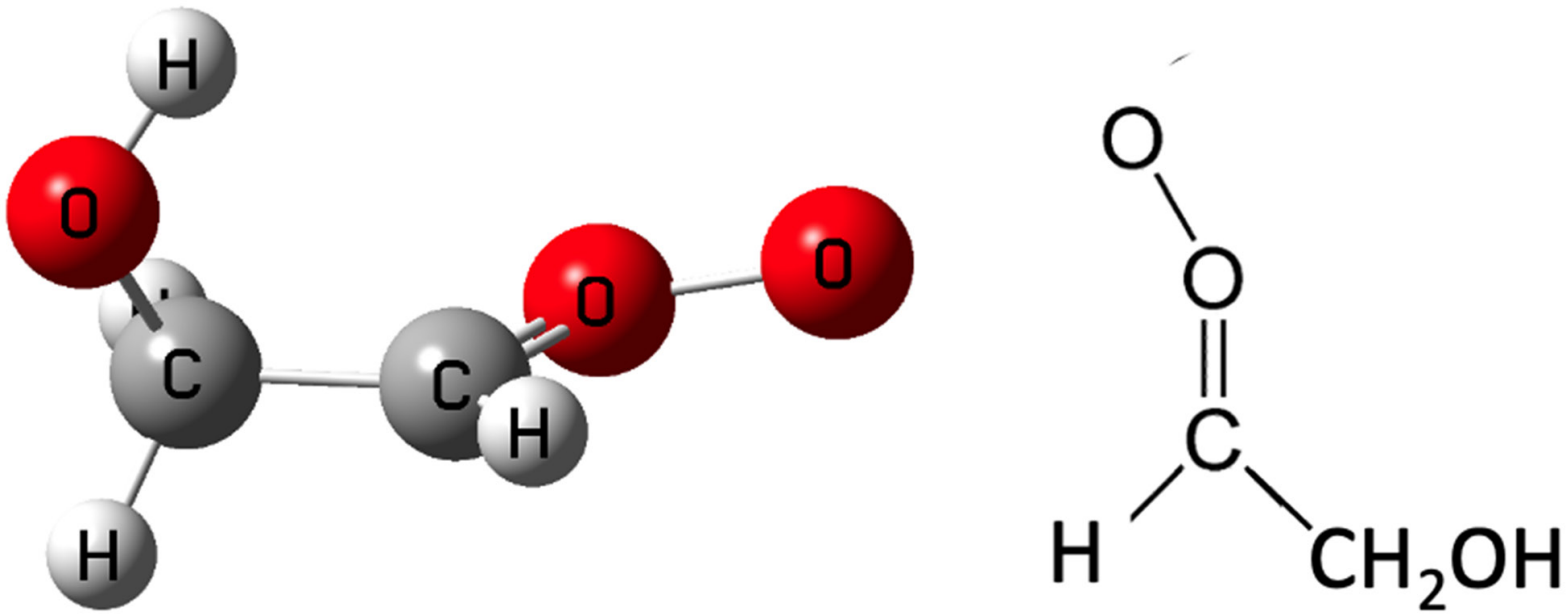
Two representations of *E*-glycolaldehyde oxide.

C5-C6 unsaturated alcohols such as *Z*-2-penten-1-ol and *E*-2-hexen-1-ol [42–47] have been found to be widespread in the atmosphere [42,48–53]. The atmospheric sink of these unsaturated alcohols is ozonolysis [42,46,54,55], and ozonolysis reactions can produce *E*-CH_2_(OH)CHOO (i.e., *E-*hydroxyethanal oxide, also known as *trans-*glycolaldehyde oxide or *anti-*glycolaldehyde oxide, where the hydroxymethyl group is *trans* to the oxide oxygen, as shown in Fig. 1) and *Z*-CH_2_(OH)CHOO [where the terminal oxygen atom is *cis* (also called *syn*) to CH_2_OH] [43]. The conversion between them requires high activation enthalpy (~40 kcal/mol at 0 K), which means they are not in equilibrium and react independently [56–58]. The relative yield of the two isomers depends on the starting alkene [58–61]. The present article focuses on the *trans* isomer.

Investigation of *E*-CH_2_(OH)CHOO kinetics has been carried out theoretically but not experimentally. There is one theoretical study, which is due to Vereecken et al. [62] who investigated the unimolecular reaction of *E*-CH_2_(OH)CHOO and its bimolecular reaction with H_2_O by using CCSD(T)//M06-2X with a beyond CCSD(T) empirical correction based on a large amount of data. Because obtaining quantitative kinetics by theoretical methods requires reliable geometrical optimizations, zero-point vibrational energies, and single-point energies [63], further theoretical calculations are required to check those results.

The present article is concerned with obtaining quantitative kinetics of the unimolecular reaction of *E*-CH_2_(OH)CHOO and the reaction of this intermediate with atmospheric water vapor by using theoretical methods. We chose *E*-CH_2_(OH)CHOO as a prototype to understand the effects of hydroxy substituents on Criegee intermediates. We consider only reactions of stabilized Criegee intermediates, and we caution that nascent Criegee intermediates may show different reactivity. The reactions considered are as follows:


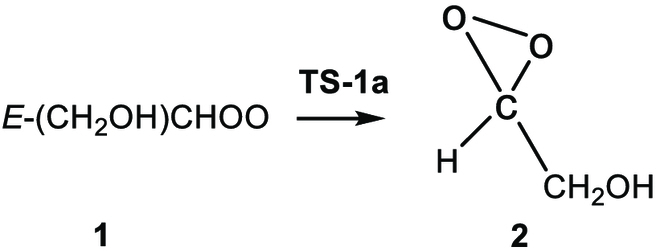
 (R1a)


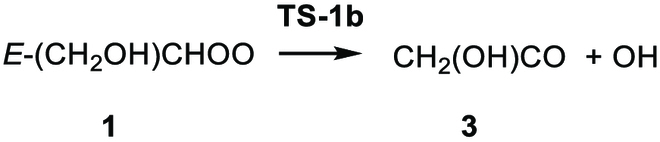
 (R1b)


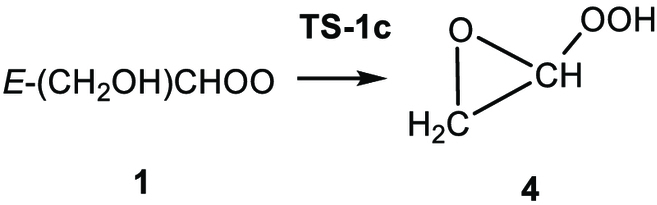
 (R1c)


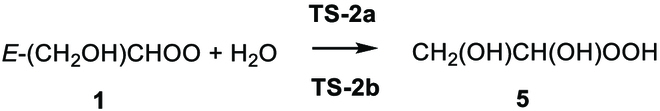
 (R2.1)


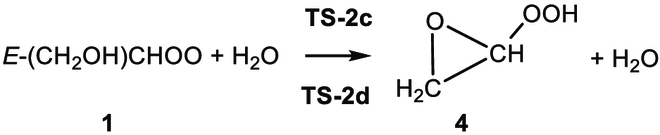
 (R2.2)


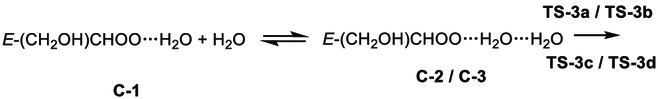
 (R3.1)


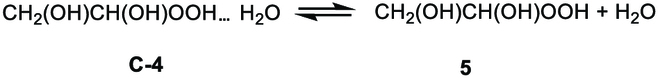
 (R4.1)


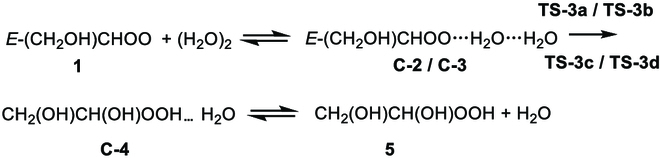
 (R3.2)


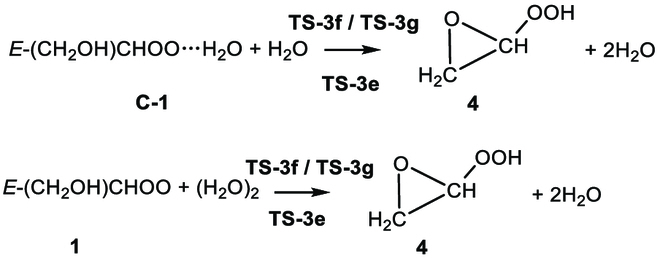
 (R4.2)

Water vapor is studied as a reactant because there are high concentrations of water monomers (~10^17^ molecules cm^−3^) [64,65] and water dimers (~10^14^ molecules cm^−3^) [56,66] in the troposphere. Moreover, previous investigations have shown that water vapor is a dominant sink for the simplest Criegee intermediate (CH_2_OO) [56,62,66–69].

## Computational Methods

### *Dynamics*: *Methods*

The rate constant calculations in the present paper were carried out by variational transition state theory (VTST) [70,71]. In the present work, we write the VTST rate constant as a product of factors.

The first factor equals the one-way equilibrium flux of reactants through a hypersurface in phase space dividing reactants from products [72–75]. The hypersurface is called the dividing surface or the transition state. When the dividing surface runs through a first-order saddle point and is normal to the imaginary-frequency normal mode, it is called the conventional transition state, and the first factor is called the conventional transition state theory rate constant. When applied to a unimolecular reaction, it is sometimes called Rice–Ramsperger–Kassel–Marcus (RRKM [76]) theory. Note that the transition state is missing one degree of freedom, which is the degree of freedom normal to the dividing surface and which is called the reaction coordinate. This first factor can be written as [77,78]

$$k_{\mathrm{TST}}=\frac{k_{B}T}{hC^{0}}\text{exp}\left[-\frac{\Delta G_{T}^{\mathrm{TS},0}}{\mathrm{RT}}\right]$$ (1)

where *k*_B_, *h*, *T*, and *R* are the Boltzmann constant, Planck constant, temperature, and gas constant, respectively; *C*^0^ is the reciprocal of the standard-state concentration for bimolecular reactions and unity for unimolecular reactions; and ${\Delta G}_{T}^{\mathrm{TS},0}$ is the conventional transition state theory standard-state free energy of activation defined by

$${\Delta G}_{T}^{\mathrm{TS},0}=G_{T}^{\mathrm{TS},0}-G_{T}^{R,0}$$ (2)

where $G_{T}^{\mathrm{TS},0}$ is the standard-state Gibbs free energy of the conventional transition state and $G_{T}^{R,0}$ is the standard-state Gibbs free energy of reactants (the subscript *T* is the temperature). Because the conventional transition state is not a stable species, Eq. 2 is a quasithermodynamic formulation. In general, the flux through the dividing surface can be calculated by classical trajectories [79–81] or by making the harmonic or quasiharmonic approximation and using the partition functions of statistical mechanics. Because the reactants are assumed to be in local equilibrium, transition state theory is sometimes called a statistical theory, but that should not obscure the fact that it is the calculation of a dynamical flux. An advantage of the partition function formulation is that one may use quantum mechanical partition functions; this replaces the classical calculation of the flux through the dividing surface by a quantized one, which is sometimes called quasiclassical. Since the transition state is missing one degree of freedom, the quasiclassical formulation quantizes all degrees of freedom except the reaction coordinate. In the present work, we use the quasiclassical partition function method with the quasiharmonic approximation.

The quasiharmonic approximation consists of using the harmonic oscillator formulas for the vibrational partition functions, but with scaled vibrational frequencies that account for anharmonicity in small-amplitude vibrations and for systematic errors in the method used to calculate vibrational frequencies. When the reacting system has multiple conformations (multiple structures), the anharmonicity of torsional vibrations can be accounted for by multiplying by the multistructural torsion (MS-T) factor *F*_act_, which equals the ratio of the multistructural rate constant [82] to the single-structural rate constant.

The final factor in the rate constant calculations is called the transmission coefficient. It may be written as a product of 3 more factors:

γ=Γκg

where Γ is the recrossing transmission coefficient, κ is the tunneling transmission coefficient, and *g* accounts for nonequilibrated reactants.

The recrossing transmission coefficient is defined as the ratio of the single-structure VTST rate constant to the single-structure conventional transition state theory rate constant. VTST again involves the flux though a surface, but the surface is chosen variationally to minimize the flux so that the one-way flux is a closer approximation to the net flux; minimizing the flux is equivalent to minimizing recrossing [70].

The tunneling transmission coefficient is the ratio of the dynamical flux calculated with tunneling and nonclassical reflection to that calculated quasiclassically. When the tunneling is calculated with a one-dimensional model, this may be considered the effect of adding quantum effects to the reaction coordinate. In our work, we use the small-curvature tunneling approximation [83], which is a multidimensional tunneling calculation that also includes nonseparable coupling of the reaction coordinate to the other degrees of freedom.

The nonequilibrium factor *g* is approximated as unity for bimolecular reactions, where the rate constant is assumed to be independent of pressure, and for the high-pressure limit (HPL) of unimolecular reactions. The final VTST rate constant for these cases is

$$k_{\mathrm{HPL}}=F_{\mathrm{act}}\;{\Gamma \kappa k}_{\mathrm{TST}}$$ (3)

where *k*_TST_ is the single-structure, quasiclassical, quasiharmonic conventional transition state theory rate constant.

For finite-pressure unimolecular reactions, one must consider the competition between depletion of energized states of the reactant due to reaction and pressure-dependent repopulation of those states by energy transfer collisions. We calculated the pressure dependence of the isomerization reaction R1a in 2 ways: system-specific quantum Rice–Ramsperger–Kassel (SS-QRRK) theory [84,85] and a master equation treatment.

### *Dynamics*: *Levels*

For R1a and R2.1, dual-level [56,57,66,86–92] multistructural [82] canonical VTST [70] with small-curvature tunneling [83] (DL-MS-CVT/SCT) [93] was used to obtain high-pressure-limit rate constants $\left(k_{\mathrm{HPL}}^{\mathrm{DL}}\right)$ by the following equation:

$$k_{\mathrm{HPL}}^{\mathrm{DL}}=\frac{k_{\mathrm{TST}}^{\mathrm{SS}-\mathrm{HL}}}{k_{\mathrm{TST}}^{\mathrm{SS}-\mathrm{LL}}}\;k_{\mathrm{MS}-\mathrm{CVT}/\mathrm{SCT}}^{\mathrm{LL}}=F_{\mathrm{act}}\;\Gamma \kappa \;k_{\mathrm{TST}}^{\mathrm{SS}-\mathrm{HL}}$$ (4)

where $k_{\mathrm{TST}}^{\mathrm{SS}-\mathrm{HL}}$ and $k_{\mathrm{TST}}^{\mathrm{SS}-\mathrm{LL}}$ denote higher-level and lower-level conventional transition state theory, respectively, employing the lowest-energy conformers of the reactants and transition states; *κ* is a tunneling transmission coefficient calculated by the small-curvature tunneling approximation for the lowest-energy reaction path; *Γ* is a recrossing transmission coefficient $\left(k_{\mathrm{CVT}}^{\mathrm{SS}-\mathrm{LL}}/k_{\mathrm{TST}}^{\mathrm{SS}-\mathrm{LL}}\right)$, calculated by canonical variation transition state theory for the lowest-energy reaction path; and *F*_act_ is the multistructural anharmonicity factor calculated by MS-T(coupled) [94]. All these calculations except $k_{\mathrm{TST}}^{\mathrm{SS}-\mathrm{HL}}$ are based on the lower level of electronic structure.

For R3.1 and R4.1, the rate-determining step at low temperatures is from the reactant to the pre-reaction complex. Therefore, for R3.1 and R4.1, we used the deep-intermediate limit [66] of canonical unified statistical theory (CUS) [95,96]

$$k_{x}=\frac{k_{x}^{\prime }k_{x}^{\prime \prime }}{k_{x}^{\prime }+k_{x}^{\prime \prime }}$$ (5)

where $k_{x}^{\prime \prime }$ is calculated for the association reaction as calculated by variable reaction coordinate variational transition-state theory [97,98] and $k_{x}^{\prime }$ is the rate constant for passing through the tight transition state (**TS-3a**, **TS-3b**, **TS-3c**, and **TS-3d**) as calculated by DL-MS-CVT/SCT) using Eq. 4. The rate constant $k_{x}^{\prime \prime }$ is for **C-1** + H_2_O → **C-2**/**C-3** for R3.1 and is for **1**+(H_2_O)_2_ → **C-2**/**C-3** for R4.1. The rate constant $k_{x}^{\prime }$ is for **C-1** + H_2_O → **5 +** H_2_O for R3.1 and is for **1** + (H_2_O)_2_ → **5 +** H_2_O for R4.1. We use CUS only in the HPL.

We explored the pressure dependence of the isomerization reaction R1a in 2 ways. (a) We used SS-QRRK theory with DL-MS-CVT/SCT rate constants as input. (b) We used the master equation with RRKM rate constants as input.

### Dynamics: Software

Polyrate 2017C and Gaussrate 2017B [99] were carried out to obtain $k_{\mathrm{TST}}^{\mathrm{SS}-\mathrm{LL}}$, $k_{\mathrm{TST}}^{\mathrm{SS}-\mathrm{HL}}$, *Γ*, and *κ*. In addition, MESS [100] and Polyrate 2017C were used for pressure dependence calculations. The torsional anharmonicity calculation and conformation search were performed using MSTor [101,102].

### Electronic structure: Acronyms and abbreviations

To make the paper easier to read and the notation less cumbersome, we use standard acronyms for electronic structure methods and basis sets. These are explained with references in Table 1.

**Table 1.** Electronic structure methods and basis sets.

Abbreviation	Explanation	Reference
CCSD(T)	Coupled cluster theory with single and double excitations and quasiperturbative connected triples	[114]
CCSD(T)-F12a	CCSD(T) with explicit correlation of type F12a	[115,116]
DF-CCSD(T)-F12b	CCSD(T) with density fitting and explicit correlation of type F12b	[117]
CCSDT(Q)	Coupled cluster theory with single, double, and triple excitations and quasiperturbative connected quadruples	[118]
CBS	Complete basis set limit obtained by extrapolation	
W2X	A composite method to approximate CCSD(T)/CBS	[107]
W2X+SC ^a^	W2X plus a semiempirical correction	
W3X-L	A composite method to approximate CCSDT(Q)/CBS	[107]
revM06	Revised Minnesota-2006 density functional	[119]
M06CR	revM06 density functional reparametrized for Criegee intermediate reactions	[66]
A/B ^b^	A calculation with electronic structure approximation A and basis set B	
A/B//C/D ^b,c^	A single-point energy calculation with method A/B at a geometry optimized by method C/D and with frequencies by method C/D.	
cc-pVDZ-F12	A polarized valence-double-zeta basis set for explicitly correlated calculations	[120]
jun-cc-pVDZ	A partially augmented polarized valence-double-zeta basis set	[121]
MG3S	A minimally augmented polarized valence-triple-zeta basis set	[122]
Level-1	CCSD(T)-F12a/cc-pVDZ-F12	
Level-2	DF-CCSD(T)-F12b/jun-cc-pVDZ	

^a^ SC is a semiempirical correction that is equal to the difference between W3X-L and W2X for the barrier of reaction through **TS-3a**.

^b^ For composite methods, A/B becomes A because the basis sets used for individual steps in the calculation are defined as part of the method.

^c^ Note that A/B//A/B is abbreviated as A/B; this is called a calculation with a consistently optimized geometry. Note that an A/B///C/D calculation in which A ≠ C and/or B ≠ D is called a single-point energy calculation.

All enthalpies of activation in this article are calculated by conventional transition state theory without tunneling and are for a temperature of 0 K. These values should not be confused with phenomenological enthalpies of activation calculated from observable rate constants because the phenomenological ones contain contributions from variational effects, recrossing, tunneling, and nonequilibrium effects [78].

### Anharmonicity

To treat anharmonicity and correct for systematic errors in calculated frequencies, we included anharmonicity by 2 methods: frequency scaling and torsional anharmonicity.

Scaling factors account for anharmonicity of small-amplitude vibrations and for systematic errors in specific combinations of density functional and basis sets [103]. Previous work has shown that these scale factors are largely transferable for calculating zero-point energies of stable species (the zero-point energy is dominated by small-amplitude, high-frequency vibrations), but sometimes they are quite significantly different for transition states and low-frequency vibrations. Two parametrizations are considered here: generic scale factors parametrized to the zero-point energies of small molecules. One is the use of scaling factors via the standard method [56,57,66], as listed in Table S1. The other is reaction-specific scale factors [87,104], which were obtained by using hybrid degeneracy-corrected second-order vibrational perturbation theory [105,106] with the MPW1K/6-311+G(2df,2p)electronic structure method as listed in Table S2.

The torsional anharmonicity was included by the multistructural method with torsional anharmonicity based on a coupled torsional potential [94]. Torsional frequencies are typically low frequencies, and they are scaled (like all other frequencies) prior to applying the multistructural method, which accounts for torsional potential anharmonicity and multiple conformational structures, when present. Note that harmonic potentials have only a single local minimum so the effect of multiple conformational structures may be considered to be an anharmonicity correction.

### Electronic structure: Higher-level methods for dynamics

Previous investigations have shown that CCSD(T)-F12a/cc-pVDZ-F12 (Level-1) can predict reliable geometries and zero-point vibrational energies (*E*_ZPE_) [66,88–90]. Therefore, for reactions R1a, R1b, R1c, and R2.1, we performed higher-level geometry optimizations and vibrational frequency calculations by using Level-1.

However, the CCSD(T) energies are not expected to be quantitatively reliable because previous investigations have indicated that Criegee intermediates are strongly correlated, which makes it necessary to perform beyond-CCSD(T) calculations in order to obtain reliable single-point energies [56,57,66,87–92]. Therefore, W3X-L//Level-1 was used for single-point energy calculations on reactions R1a and R2.1. Previous validation studies indicate that this puts our results close to the very accurate CCSDT(Q)/CBS level [56,57,66,107].

For R3.1 and R4.1, we performed higher-level geometry optimizations and frequency calculations by DF-CCSD(T)-F12b/jun-cc-pVDZ (Level-2), because this level has been validated in previous [66] investigations of the similar reaction CH_2_OO + (H_2_O)_2_. In addition, the reliability of Level-2 is also discussed in the “Electronic energies: Reactions involving 2 water molecules” section.

For the barrier of the transition states of reactions R3.1 and R4.1, we used single-point calculations. For **TS-3a**, we used W3X-L//Level-2 to reach an accuracy close to CCSDT(Q)/CBS. For **TS-3b, TS-3c**, and **TS-3d**, we used W2X//Level-2 + SC. For R3.1, the semiempirical correction was taken to be 0.60 kcal/mol for the transition structures and 0 for reactants. For R4.1, it is 0.83 kcal/mol for transition structures and 0 for reactants.

The empirical correction (0.6 kcal/mol) of R3.1 in the current article is derived from our calculations [W3X-L//DF-CCSD(T)-F12a/jun-cc-pVDZ] obtained for the most stable transition state structure (**TS-3a**) relative to the most stable complex and water (**C-1** + H_2_O), and the empirical correction of R4.1 (0.83 kcal/mol) is derived from the most stable transition state structure (**TS-3a**) relative to the most stable reactant [**1** + (H_2_O)_2_] using W3X-L//DF-CCSD(T)-F12a/jun-cc-pVDZ. In comparison, we note that the calculated results in a previous paper [65] show that the beyond-CCSD(T) difference (between W3X-L and W2X) is a very similar 0.72 kcal/mol for different transition states in the CH_2_OO+(H_2_O)_2_ reaction. These similarities confirm the reasonableness of these empirical corrections for these specific reactions.

### Electronic structure: Lower-level methods for dynamics

The choice of a lower-level method is explained in “Electronic energies: Unimolecular reactions of **1**,” “Electronic energies: Bimolecular reactions of *E*-CH_2_(OH)CHOO + H_2_O,” and “Electronic energies: Reactions involving 2 water molecules” sections.

### Electronic structure software

For the electronic structure calculations of coupled cluster theory, we used Molpro 2019 [108] and MRCC code [109], while calculations based on density functional theory (DFT) were performed by Gaussian 16 [110].

## Results and Discussion

### Electronic energies: Unimolecular reactions of **1**

There are 5 conformers for **1** as listed in Table S3. The W2X//M06CR/MG3S and M06CR/MG3S methods yield different structures as the global minimum. Here, we used the lowest-energy structure of **1** as obtained by W2X//M06CR/MG3S.

**Fig. 2.**
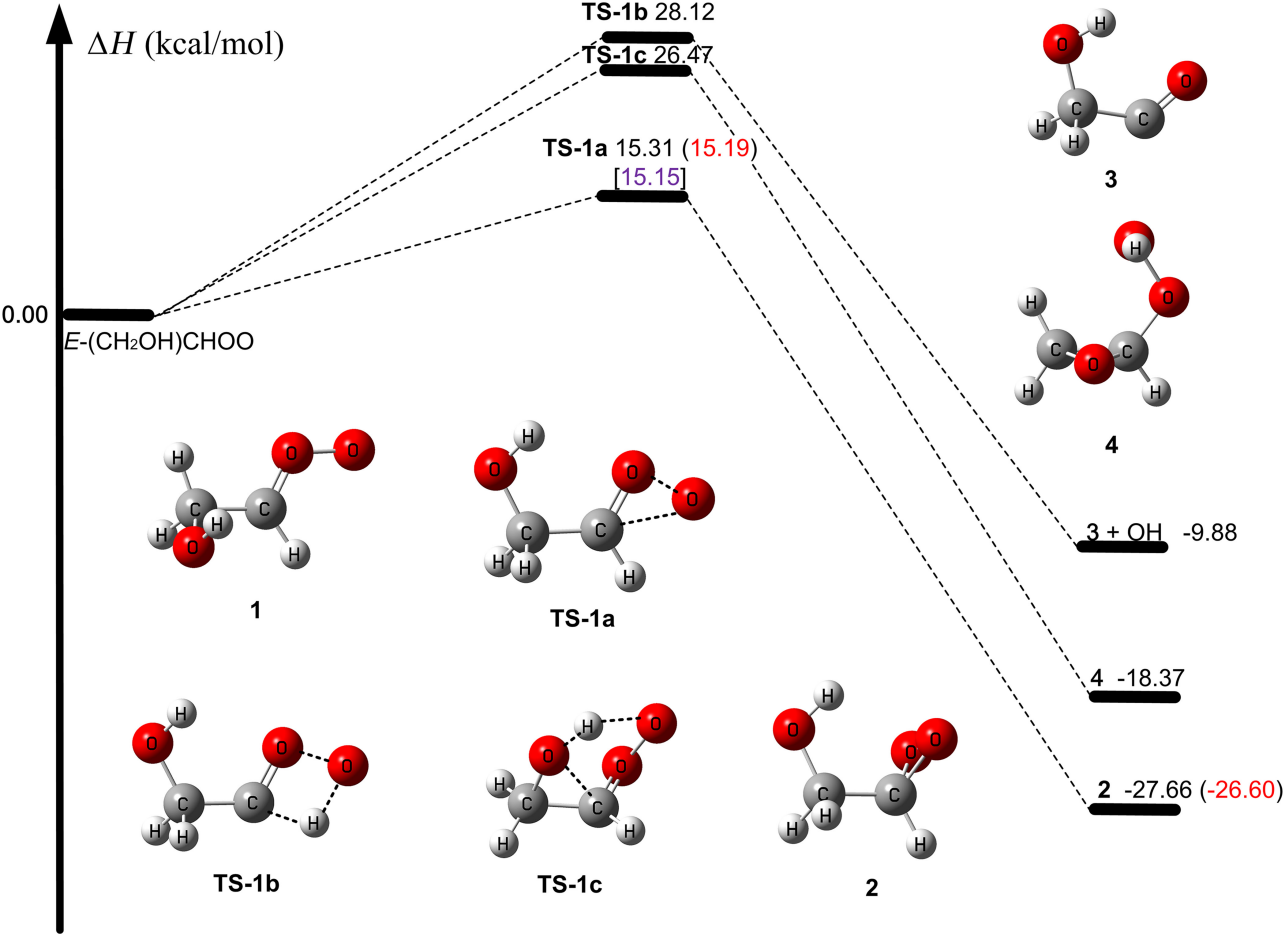
The relative enthalpy profile at 0 K (in kcal/mol) for unimolecular reactions of 1 calculated by W2X//Level-1. The values in parentheses are obtained using W3X-L//Level-1. The values in brackets are obtained using W3X-L//Level-1 with reaction-specific scale factors.

The enthalpy profile of reaction R1 at 0 K as obtained at W3X-L//Level-1 and W2X//Level-1 is depicted in Fig. 2. We found that there are 3 reaction pathways for the unimolecular reactions of *E*-CH_2_(OH)CHOO (**1**), in agreement with previous literature [56–62,66,67,91]. They are 1,3-ring closure via reaction R1a for the formation of dioxirane, 1,3-H-transfer via reaction R1b for the formation of **3** + OH [56–62,66,67,91], and a 1,2-insertion of -OH mechanism to produce **4** [62,111].

The activation enthalpy at 0 K ($\text{∆}H_{0}^{‡}$) of unimolecular rearrangement R1a is estimated to be 15.19 kcal/mol at W3X-L//Level-1 as depicted Fig. 2, which is 11 to 13 kcal/mol lower than those of reactions R1b and R1c. Therefore, unimolecular reaction of *E*-CH_2_(OH)CHOO (**1**) is dominated by **TS-1a**, and we do not consider the reaction pathways **TS-1b** and **TS-1c** in kinetics calculations.

The enthalpy of activation (15.19 kcal/mol) at 0 K for the unimolecular reaction via **TS-1a** is 3.84, 8.51, and 0.44 kcal/mol lower than those of the unimolecular ring closure reactions of CH_2_OO, *Z*-CH_3_CHOO, and *E*-CH_3_CHOO calculated by W3X-L//Level-1 [56]. Moreover, the enthalpy of activation at 0 K via **TS-1a** is 4.41 kcal/mol lower than that of the ring closure reaction of *Z*-CH_2_(OH)CHOO calculated by CCSD(T)//M06-2X [62,112]. This shows that the hydroxyl group in *E*-CH_2_(OH)CHOO enhances the reactivity for the unimolecular ring closure reaction.

$\text{∆}H_{0}^{‡}$ for **TS-1a** (15.19 kcal/mol) is 0.59 kcal/mol higher than the value reported in the literature that was obtained by CCSD(T)//M06-2X [62]. A Boltzmann factor corresponding to 0.59 kcal/mol would decrease the rate constants by a factor of 2.7 at 298 K. Moreover, the beyond CCSD(T) contribution is only 0.12 kcal/mol for **TS-1a** in Table 2, which is much lower than that of the average deviation (0.53 kcal/mol) between W2X and W3X-L in a previous investigation [66]. This shows that the beyond-CCSD(T) contribution is reaction specific, which implies that high-accuracy quantum chemical calculations are needed for obtaining quantitative relative enthalpies of activation.

**Table 2.** The enthalpy of activation at 0 K ($\text{∆}H_{0}^{‡}$ ^a^ in kcal/mol) calculated for reaction R1a. ^a^

Level	**TS-1a**	UD ^b^
W3X-L//Level-1 ^c^	15.19	0.00
M11-L/MG3S	15.28	0.09
W2X//Level-1	15.31	0.12
CCSD(T)//M06-2X	14.60 ^d^	0.59
MN15-L/MG3S	14.49	0.70
M06CR/MG3S	17.21	2.02

^a^ $\text{∆}H_{0}^{‡}$ is the enthalpy of activation at 0 K. *E*_ZPE_ is scaled by the generic scale factors.

^b^ UD denotes unsigned deviation from the best estimate.

^c^ W3X-L//Level-1 is our best estimate for **TS-1a**.

^d^ This datum is from [62].

For reaction R1a, $\text{∆}H_{0}^{‡}$ using M11-L/MG3S differs from our best estimate by only 0.09 kcal/mol in Table 2. Thus, we use M11-L/MG3S for direct dynamics calculations for R1a.

### Electronic energies: Bimolecular reactions of *E*-CH_2_(OH)CHOO + H_2_O

As depicted in Fig. 2, 2 different mechanisms, R2.1 and R2.2, are considered for the *E*-CH_2_(OH)CHOO (1) + H_2_O reaction. Details of the electronic structure calculations for **TS-2a** and **TS-2b** are provided in Table 3 and Table S4.

**Table 3.** The enthalpies of activation at 0 K ($\text{∆}H_{0}^{‡}$ in kcal/mol) for reaction R2.1.

Level	**TS-2a**	**TS-2b**	MUD ^a^
W3X-L//Level-1 ^b^	−0.09	0.60	0.00
M06CR/MG3S	0.20	0.91	0.30
MN15-L/MG3S	−0.65	0.12	0.52
W2X//Level-2	−0.73	0.02	0.65
W2X//Level-1	−0.73	−0.05	0.65
M11-L/MG3S	−0.89	−0.94	1.17

^a^ MUD is the mean unsigned deviation from the best estimate.

^b^ W3X-L//Level-1 is our best estimate for **TS-2a** and **TS-2b**. *E*_ZPE_ is scaled by the generic scale factors.

Our best estimate of $\text{∆}H_{0}^{‡}$ for **TS-2a** is −0.09 kcal/mol, which is 1.11 kcal/mol higher than that of the previous investigation [62].

We find that $\text{∆}H_{0}^{‡}$ for reaction R2.1 is lower than that for *E*-CH_3_CHOO + H_2_O by 1.29 kcal/mol at the W3X-L//QCISD/cc-pVDZ level [56]. This again shows that the reaction of *E*-CH_2_(OH)CHOO (**1**) with H_2_O is enhanced by the introduction of an OH group in Criegee intermediates. The energetically most favorable intermediates have an H-bond that increases their rigidity and thus reduces their contribution to the population.

Table 3 shows that the mean unsigned deviation (MUD) of W2X//Level-1 is 0.65 kcal/mol relative to W3X-L//Level-1 for reaction R2.1; this corresponds to a Boltzmann factor of 3.8 at 240 K. This again shows the need for beyond CCSD(T) calculations.

Table 3 shows that M06CR/MG3S (using the M06CR exchange-correlation functional previously re-parameterized for reactions of Criegee intermediates) performs well in describing reaction R2.1, and it significantly exceeds the accuracy of the popular CCSD(T)/CBS level. Therefore, we selected it to do direct dynamics calculations.

**Fig. 3.**
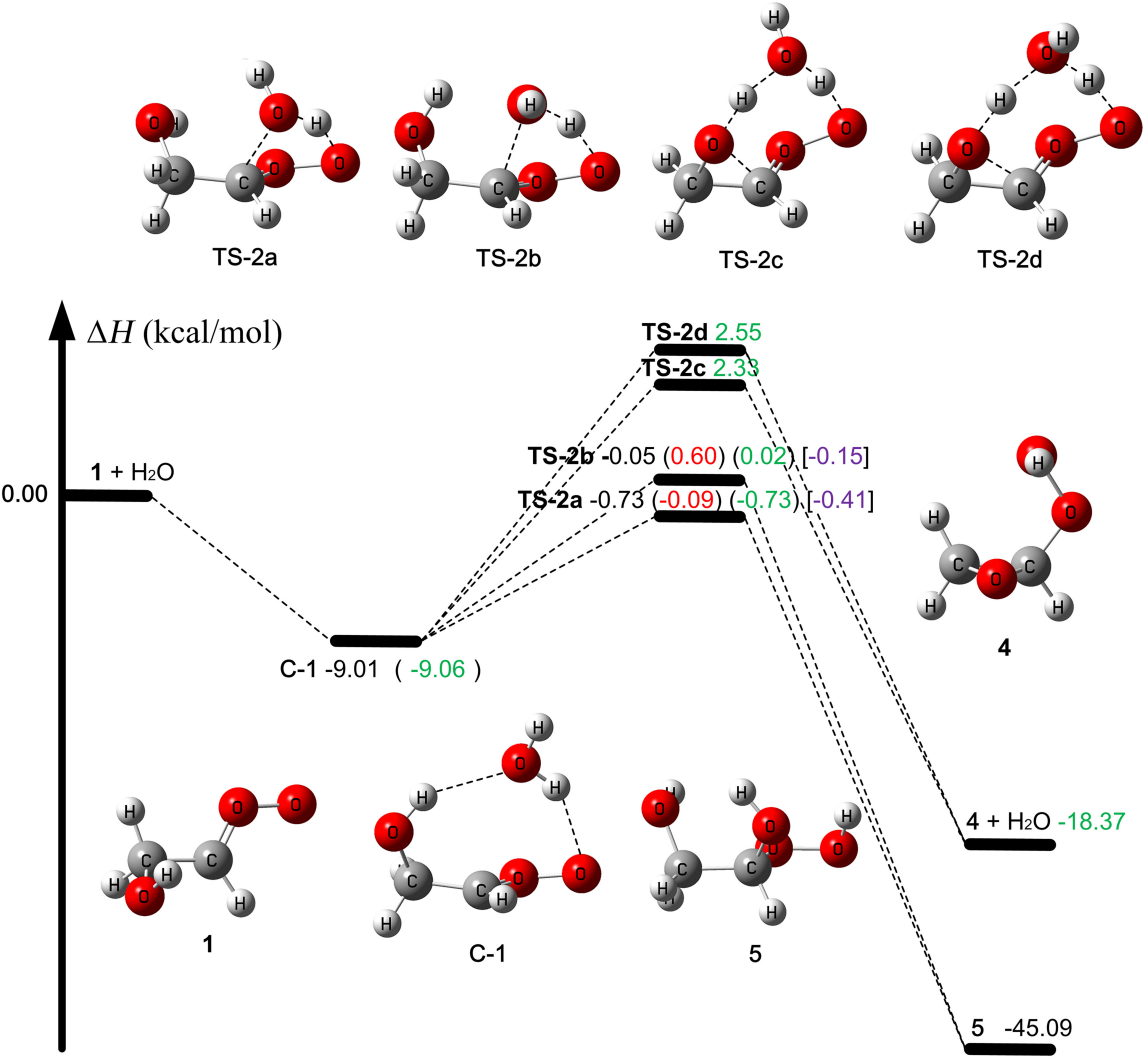
The relative enthalpy profile at 0 K (in kcal/mol) for 1 + H_2_O. The black value is calculated using W2X//Level-1. The red values in parentheses are the enthalpies of activation at 0 K calculated by W3X-L//Level-1. The green values come from W2X//Level-2. The values in brackets are obtained using W3X-L//Level-1 with reaction-specific scale factors.

One mechanism (R2.1) is an addition coupled to a hydrogen transfer reaction, which is the usual mechanism for reactions of Criegee intermediates with H_2_O [56,57,66–69]. It begins with the formation of a pre-reaction hydrogen-bonded complex **C-1**. Table S5 also shows that the W2X//M06CR/MG3S and M06CR/MG3S methods predict the same structure to be the lowest-energy structure of **C-1**. In the next step, **C-1** proceeds through **TS-2a** or **TS-2b** to isomerize into hydroxyl hydroperoxide **5** (see Fig. 3). The 2 transition states, **TS-2a** and **TS-2b,** differ in the orientation of the dangling H of H_2_O as shown in Fig. 3.

The other mechanism (reaction R2.2) is a 1,2-insertion of OH catalyzed by water as shown in Fig. 3. Specifically, the complex **C-1** undergoes isomerization via the transition state with transition structures **TS-2c** and **TS-2d**, which differ due to different orientations of H_2_O. In **TS-2c**, water acts as a bridge to enable the H atom from the hydroxyl group of **1** to transfer to the terminal oxygen, while simultaneously the oxygen atom of the OH group in **1** attacks the central carbon atom of **1**; this mechanism leads to the formation of **4**.

The most stable transition state of reaction R2.1 is **TS-2a**, and its $\text{∆}H_{0}^{‡}$ is 3.06 kcal/mol lower than that of **TS-2c** as calculated by W2X//Level-2 as shown in Fig. 3. Therefore, reaction R2.2 is not considered for kinetics calculations.

We also calculated the barrier for the isomerization of product **5** based on M06CR/MG3S. The results are in Fig. S1, and they show that the decomposition of **5** is not feasible in the atmosphere.

### Electronic energies: Reactions involving 2 water molecules

We first confirm the reliability of optimized geometries and calculated zero-point vibrational energies by using DF-CCSD(T)-F12b/jun-cc-pVDZ (Level-2). Our calculated results (see Table 3) show that the difference of MUD in $\text{∆}H_{0}^{‡}$ between W2X//Level-1 and W2X//Level-2 is less than 0.01 kcal/mol. This shows that Level-2 can obtain reliable optimized geometries, compared with Level-1. The difference in zero-point vibrational energies (see Table S3) between Level-1 and Level-2 is less than 0.04 kcal. We conclude that Level-2 can produce reliable zero-point vibrational energies, compared with Level-1. The reliability of Level-2 has also been shown for other Criegee reactions [66,88–90]. Therefore, we will use Level-2 to do geometrical optimization and frequency calculations in the reactions involving 2 water molecules.

We considered 2 reaction mechanisms for reactions R3 and R4, i.e., reactions R3.1 and R3.2 for R3 and reactions R4.1 and R4.2 for R4.

**Fig. 4.**
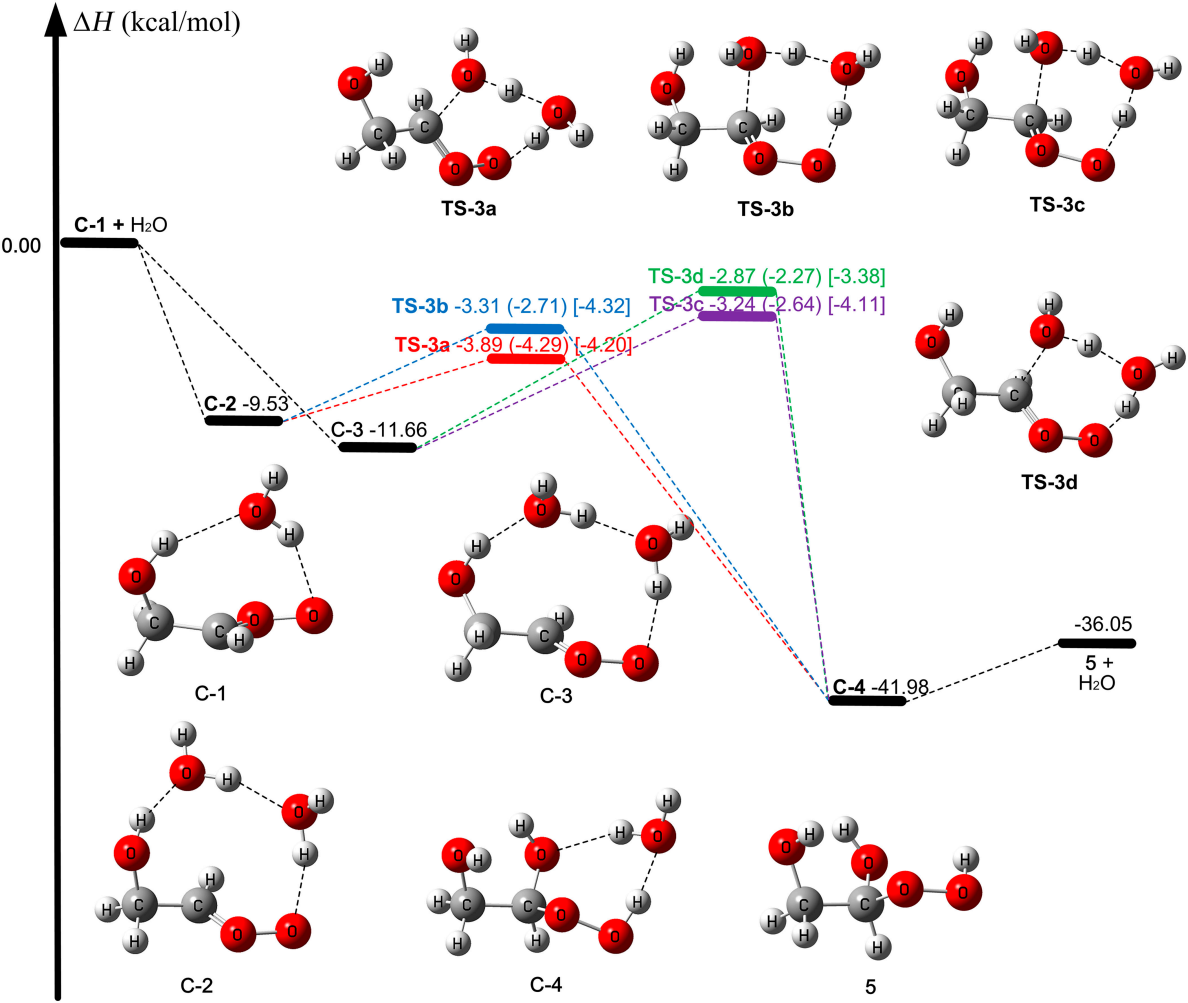
Relative enthalpy (0 K) with respect to C-1 and H_2_O (in kcal/mol). All values were calculated by W2X//Level-2 except that the results in parentheses are obtained by W3X-L//Level-2 for TS-3a and by SC+W2X//Level-2 for TS-3b, TS-3c, and TS-3d. The values in brackets are obtained using SC+W2X//Level-2 with reaction-specific scale factors.

In reactions R3.1 (see Fig. 4) and R4.1 (see Fig. S2), a water molecule acts as a bridge to catalyze the addition-coupled hydrogen transfer reaction via **TS-3a**, **TS-3b**, **TS-3c**, and **TS-3d**; this mechanism is similar to that for reaction of other Criegee intermediates with (H_2_O)_2_ [66–69]. In R3.1, the reaction occurs by **C-1** colliding with a water monomer responsible for the formation of 2 conformers of *E*-CH_2_(OH)CHOO…(H_2_O)_2_, labeled as **C-2** and **C-3**. In addition, C-2 and C-3 can also be formed by a water dimer colliding with *E*-CH_2_(OH)CHOO (**1**).

Reactions R3.2 and R4.2 are a 1,2-insertion of the OH group catalyzed by 2 H_2_O via **TS-3e**, **TS-3f**, and **TS-3g** as shown in Figs. S3 and S4. In these transition states, the 2 H_2_O together act as a bridge to transfer H from OH in Criegee intermediates (a triple H atom transfer process) and concert with the oxygen atom of OH to attack the central carbon atom. However, the lowest transition state (**TS-3e**) of reaction R3.2 is 7.58 kcal/mol higher than that (**TS-3a**) of reaction R3.1 as calculated by M06CR/MG3S (see Table 4 and Fig. S3); this leads to a Boltzmann factor of 8 × 10^6^ at 240 K. Similarly, for R4.2, it is still not feasible in terms of $\text{∆}H_{0}^{‡}$ comparing R4.1 (see Fig. S4). Therefore, we do not consider R3.2 and R4.2 for kinetics calculations.

**Table 4.** The enthalpies of activation (kcal/mol) at 0 K ($\text{∆}H_{0}^{‡}$ ^a^ in kcal/mol) for reaction R3.1 of C-1 + H_2_O calculated at 4 transition states. ^a^

Level	**TS-3a**	**TS-3b**	**TS-3c**	**TS-3d**	MUD ^b^
SC+W2X//Level-2 ^c^	−3.29	−2.71	−2.64	−2.27	0.00
M06CR/MG3S	−2.64	−2.14	−2.15	−1.69	0.57
W2X//Level-2	−3.89	−3.31	−3.24	−2.87	0.60
M11-L/MG3S	−2.26	−2.02	−2.02	−1.06	0.89
MN15-L/MG3S	2.38	2.74	2.86	3.40	5.57

^a^ $\text{∆}H_{0}^{‡}$ is the enthalpy of activation at 0 K relative to **C-1** + H_2_O. *E*_ZPE_ is scaled by the generic scale factors.

^b^ MUD denotes mean unsigned deviation from the best estimate (SC+W2X//Level-2).

^c^ SC+W2X//Level-2 includes the semiempirical correction (SC), which equals $\text{∆}H_{0}^{‡}$ result for **TS-3a** relative to **C-1** + H_2_O using W3X-L//Level-2 minus $\text{∆}H_{0}^{‡}$ for **TS-3a** relative to **C-1** + H_2_O using W2X//Level-2. By this definition, SC+W2X//Level-2 is the same as to W3X-L//Level-2 for $\text{∆}H_{0}^{‡}$ of **TS-3a** relative to **C-1** + H_2_O.

Compared with CH_2_OO + (H_2_O)_2_ [66], $\text{∆}H_{0}^{‡}$ relative to **1** + (H_2_O)_2_ by W3X-L//Level-2 is 3.6 kcal/mol lower than that of CH_2_OO + (H_2_O)_2_ calculated by using W3X-L//CCSD(T)-F12a/cc-pVTZ-F12 level [66].

For direct dynamics calculations of the kinetics, we made a semiempirical correction (SC) to better select the functional with the best performance. Table 4 shows that compared to the results of SC+W2X//Level-2, MUD (R3.1) of $\text{∆}H_{0}^{‡}$ is 0.57 kcal/mol using M06CR/MG3S. The imaginary frequencies are also listed in Table S6, where they are compared to the results obtained with Level-2; MUD between Level-2 (best estimate) and M06CR/MG3S for the 4 transition structures is only 45i cm^−1^. This supports using M06CR/MG3S for direct dynamics calculations of **C-1** + H_2_O and **1** + (H_2_O)_2_ (R3.1 and R4.1).

### Effect of anharmonicity on activation enthalpy

Tables S1 and S2 show that the reaction-specific scale factors for **TS-3a** (0.966), **TS-3b** (0.956), **TS-3c** (0.958), and **TS-3d** (0.963) differ very significantly from the generic scale factor (0.981 for Level-2). Table S7 shows that for many channels, this makes a significant change in the calculated enthalpy of activation. For example, $\text{∆}H_{0}^{‡}$ of **TS-3b** for reaction R3.1 is decreased by 1.61 kcal/mol, when anharmonicity is calculated using reaction-specific scale factors; this shows the importance of anharmonicity for controlling rate constants in atmospheric chemistry.

### Kinetics

Reaction R1 refers to the sum of reactions R1a, R1b, and R1c, but since we will see that pathways 1b and 1c have much higher barriers than 1a, our rate constant *k*_1_ for reaction R1 will be limited to pathway 1a. We will see that our calculations predict that the lowest-energy transition state of reaction R2.1 is ~3 kcal/mol lower than that of reaction R2.2, and so, we will not consider kinetics for reaction R2.2. Reaction R3 refers to the sum of R3.1 and R3.2, and reaction R4 refers to the sum of reactions R4.1 and R4.2. We will see that reactions R3.2 and R4.2 and reaction paths through transition states **TS-3e**, **TS-3f**, and **TS-3g** make insignificant contributions to the rates, and therefore, we will calculate the rate constants of R3 and R4 by considering only mechanisms R3.1 and R4.1 and only transition states **TS-3a**, **TS-3b**, **TS-3c**, and **TS-3d** for these mechanisms. In dynamics calculations on reactions R3.1 and R4.1, we consider both reaction through the loose transition state leading from **1** + (H_2_O)_2_ to **C-2/C-3** and reaction through the tight transition states **TS-3a**, **TS-3b, TS-3c,** and **TS-3d**. However, only tight transition states are considered for dynamics calculations on other reactions in this work.

We also consider the pressure effects on the unimolecular reaction of *E*-CH_2_(OH)CHOO (**1**) in R1a.

Table 5 and Tables S8 to S20 provide rate constants. It can be seen from Table 5 that *k*_3.1_ and *k*_4.1_ are much greater than *k*_2_, and this has the effect that bimolecular reactions involving 2 water molecules are more important than those involving only one water. We fitted the rate constants to [113]

$$k=A{\left(\frac{T+T_{0}}{300}\right)}^{n}\text{exp}\left[-\frac{E\left(T+T_{0}\right)}{R\left(T^{2}+T_{0}^{2}\right)}\right]$$ (6)

**Table 5.** Rate constants in the high-pressure limit ^a^^,^^b^ as calculated with reaction-specific scale factors.

*T*/K	*k*_1_ ^a^	*k*_2_ ^a^	*k*_3.1_	*k*_4.1_ ^a^
220	5.81 × 10^−3^	6.88 × 10^−14^	8.54 × 10^−10^	9.07 × 10^−10^
230	2.66 × 10^−2^	5.95 × 10^−14^	7.20 × 10^−10^	9.02 × 10^−10^
250	3.92 × 10^−1^	4.67 × 10^−14^	2.61 × 10^−10^	8.89 × 10^−10^
270	3.92 × 10^0^	3.84 × 10^−14^	6.22 × 10^−11^	8.25 × 10^−10^
290	2.87 × 10^1^	3.29 × 10^−14^	1.85 × 10^−11^	5.69 × 10^−10^
298	5.93 × 10^1^	3.12 × 10^−14^	1.24 × 10^−11^	4.25 × 10^−10^
300	7.06 × 10^2^	3.07 × 10^−14^	1.13 × 10^−11^	3.91 × 10^−10^
320	3.62 × 10^2^	2.75 × 10^−14^	4.99 × 10^−12^	1.44 × 10^−10^
350	2.97 × 10^3^	2.41 × 10^−14^	1.95 × 10^−12^	3.12 × 10^−11^

^a^ *k*_1_, *k*_2_, *k*_3.1_, and *k*_4.1_ are rate constants for reactions R1, R2, R3.1, and R4.1, respectively. The units are s^−1^ for the unimolecular reaction (*k*_1_) and cm^3^ molecule^−1^ s^−1^ for the bimolecular reactions (the other 3).

^b^ The rate constants in this table are all are obtained by the dual-level strategy, and the results of higher- and lower-level calculations can be found in the Supplementary Materials (see Tables S8 to S20).

where *R* is the gas constant (1.987212 × 10^−3^ kcal mol^−1^ K^−1^) and *A*, *n*, *T*_0_, and *E* are fitted parameters for each reaction and are listed in Table S21. We also calculated the Arrhenius activation energies *E*_a_ (which are local slopes of Arrhenius plots) of each reaction at 190 to 350 K by

$$E_{a}=\frac{E\left(T^{4}+2T_{0}T^{3}-T_{0}^{3}T^{2}\right)}{{\left(T^{2}+T_{0}^{2}\right)}^{2}}+\frac{\mathrm{nR}T^{2}}{T+T_{0}}$$ (7)

and the results are presented in Table S22.

The reaction rate of **1** with H_2_O is expressed by

$$\begin{array}{c} v_{1}=-\frac{d\left[E-{\mathrm{CH}}_{2}\left(\mathrm{OH}\right)\text{CHOO}\right]}{\mathrm{dt}}\\ =K_{\mathrm{eq}1}k_{3.1}\left[H_{2}O\right]\left[H_{2}O\right]\left[E-{\mathrm{CH}}_{2}\left(\mathrm{OH}\right)\text{CHOO}\right] \end{array}$$ (8)

where *K*_eq1_ is the equilibrium constants for forming complex **C-1** and *k*_3.1_ is the bimolecular rate constant of **C-1** + H_2_O (R3.1). The reaction rate of **1** + (H_2_O)_2_ is expressed by

$$\begin{array}{c} v_{2}=-\frac{d\left[E-{\mathrm{CH}}_{2}\left(\mathrm{OH}\right)\text{CHOO}\right]}{\mathrm{dt}}\\ =K_{\mathrm{eq}2}k_{4.1}\left[H_{2}O\right]\left[H_{2}O\right]\left[E-{\mathrm{CH}}_{2}\left(\mathrm{OH}\right)\text{CHOO}\right] \end{array}$$ (9)

where *K*_eq2_ is the equilibrium constants for forming (H_2_O)_2_ and *k*_4.1_ is the bimolecular rate constant of **1** + (H_2_O)_2_ (R4.1). The rate ratio (*v*_1_/*v*_2_) is defined by

$$\frac{v_{1}}{v_{2}}=\frac{K_{\mathrm{eq}1}k_{3.1}}{K_{\mathrm{eq}2}k_{4.1}}$$ (10)

and is given in Table 6, where for each altitude we use the temperature of a standard atmosphere at that altitude.

**Table 6.** Equilibrium constants and the rate ratio.

Height (km) ^a^	*T* (K) ^a^	*K*_eq1_ ^b^	*K*_eq2_ ^c^	*v*_1_/*v*_2_
0	288.8	6.76 × 10^−20^	2.44 × 10^−21^	1.27
5	259.3	3.87 × 10^−19^	4.62 × 10^−21^	11.6
10	229.7	5.58 × 10^−18^	1.05 × 10^−20^	4,115
15	212.6	1.65 × 10^−17^	1.90 × 10^−20^	796
20	215.5	1.23 × 10^−17^	1.71 × 10^−20^	595
25	218.6	9.20 × 10^−18^	1.53 × 10^−20^	447
30	223.7	5.77 × 10^−18^	1.29 × 10^−20^	274
35	235.1	2.25 × 10^−18^	8.93 × 10^−21^	96.1
40	249.9	7.14 × 10^−19^	5.87 × 10^−21^	35.7
45	266.1	2.48 × 10^−19^	3.95 × 10^−21^	6.75
50	271.0	1.83 × 10^−19^	3.49 × 10^−21^	4.73

^a^ Data are from [65].

^b^ *K*_eq1_ is the equilibrium constant between **C-1** and **1** + H_2_O (in molecules cm^−3^). For the calculation of this equilibrium constant, we considered all the conformers; details are given in the Supplementary Materials (see Table S5).

^c^ *K*_eq2_ is the equilibrium constant between water dimer and water monomer from the literature in our previous investigation (in molecules cm^−3^) [123].

We find that the overall rate (*v*_1_) through R3.1 is much faster than that (*v*_2_) through R4.1 at low temperature, with factors of 4,115 and 796 at 229.7 and 212.6 K, respectively. However, Table 6 shows that *v*_1_ and *v*_2_ are closer at higher temperature, with values decreasing from 35.7 at 249.9 K to 1.27 at 288.8 K.

The complexes formed by Criegee intermediates and water monomer may react with water monomer more rapidly than Criegee intermediates react directly with atmospheric water dimers, and this phenomenon may be common in many similar reactions of Criegee intermediates with 2 water molecules. For example, *E*-CH_3_CHOO^…^H_2_O + H_2_O may be more important than *E*-CH_3_CHOO + (H_2_O)_2_ at low temperatures. This has been not considered in the literature [67,68].

Tables S8 to S18 also provide the transmission coefficients and the multistructural torsional anharmonicity factors (*F*_act_). For **TS-1a**, we find that recrossing effects decrease the rate constants by only about 1%. The torsional anharmonicity of **TS-1a** increases the rate constant by 50% to 80%.

For R2.1, the torsional anharmonicity decreases the rate constants by about 40% for **TS-2a** and increases them by 15% to 31% for **TS-2b**. Recrossing effects for **TS-2b** cause the rate constant to drop by roughly 30% at 298 K.

For reaction R3.1, the effects of these 3 factors are larger. For example, at 298 K, torsional anharmonicity and recrossing effects for **TS-3a** decrease the rate constant by 39% and 47%, respectively, while tunneling effects increase the rate by a factor of approximately 2.

To consider whether tunneling is fast enough to allow reaction R3.2 to compete with reaction R3.1, we calculated tunneling transmission coefficient for the reaction path through **TS-2c**, and the results are listed in Table S23. This calculation shows that although tunneling increases the rate through **TS-3c** by a factor of 17 at 190 K, this is not enough to overcome the difference of $\text{∆}H_{0}^{‡}$ (a Boltzmann factor of 3,309 at 190 K when comparing **TS-2c** and **TS-1a**). For this reason, it is not necessary to include **TS-2c** and **TS-2d** in the kinetics calculations.

Tables S8 to S18 show that anharmonicity has a very large effect on quantitative rate prediction. For example, for reaction R3.1 through transition state **TS-3b**, the rate calculated using reaction-specific scale factors is 12 to 77 times faster than the rate calculated using generic scale factors at 190 to 350 K (see Table S12).

The rate constants for R1a and R2.1 calculated in a previous investigation [62] are 2.4 and 6.1 times faster, respectively, than the values in the present work at 298 K. We find that the reaction rate of reaction R2.1 (3.12 × 10^−14^ cm^3^ molecule^−1^ s^−1^ at 298 K in this work) is larger than the reaction rates of CH_2_OO, *Z*-CH_3_CHOO, and *E*-CH_3_CHOO with H_2_O (2.4 × 10^−16^, 1.9 × 10^−19^, and 5.2 × 10^−15^ cm^3^ molecule^−1^ s^−1^ at 298 K, respectively) [56], and this results in a shorter atmospheric lifetime for the present reactant. Therefore, water vapor is regarded as a more important sink for **1** than for the Criegee intermediates without a hydroxy substituent.

The RRKM/ME and SS-QRRK method give almost the same results in Tables S24 and S25. Therefore, we use the SS-QRRK results to consider the pressure dependence. For the unimolecular reaction (**TS-1a**) of **1**, Table S26 shows that the transition pressure, which is defined as the pressure at which the unimolecular rate constant is half of the high-pressure-limit value, changes from 1.3 × 10^−3^ bar to 0.11 bar over the temperature range from 200 to 350 K. This indicates that the pressure dependence should be considered under atmospheric conditions, that is, one should consider both the temperature dependence and the pressure dependence, as given in Tables S24 to S26.

### Atmospheric implications

Table 7 gives the lifetimes of *E*-CH_2_(OH)CHOO (**1**) with respect to its unimolecular isomerization R1a (*τ*_1_) and with respect to the reactions involving R2.1 (*τ*_2_), R3.1(*τ*_3.1_), and R4.1(*τ*_4.1_). The lifetimes with respect to the bimolecular reactions are given by

$$\tau_{2}=\frac{1}{k_{2}\left[H_{2}O\right]}$$ (11)

**Table 7.** Atmospheric lifetime as a function of height. ^a^

*h* (km) ^a^	*T* (K) ^a^	*P* (mbar) ^a^	[H_2_O] ^a^	*τ*_1_ (*s*) ^b^	*τ*_2_ (*s*) ^b^	*τ*_3.1_ (*s*) ^b^	*τ*_4.1_ (*s*) ^b^
0	288.8	1,013.25	4.40 × 10^17^	4.05 × 10^−2^	6.86 × 10^−5^	3.06 × 10^−6^	3.88 × 10^−6^
5	259.3	542	1.70 × 10^16^	8.44 × 10^−1^	1.39 × 10^−3^	7.17 × 10^−5^	8.36 × 10^−4^
10	229.7	269	3.00 × 10^14^	4.05 × 10^1^	5.58 × 10^−2^	2.77 × 10^−3^	1.14 × 10^0^

^a^ The temperature (*T)* and pressure (*P*) as functions of height (*h*) and water concentrations (molecules cm^−3^) are from [65].^b^The lifetimes of E-CH2(OH)CHOO with respect to its unimolecular isomerization by reaction R1a (*τ*1) and with respect reactions R2.1 (*τ*2), R3.1(*τ*3.1), and R4.1(*τ*4.1).

for reaction with H_2_O, by

$$\tau_{3.1}=\frac{1}{K_{\mathrm{eq}1}k_{3.1}{\left[H_{2}O\right]}^{2}}$$ (12)

for the **C-1** + H_2_O reaction, and by

$$\tau_{4.1}=\frac{1}{K_{\mathrm{eq}2}k_{4.1}{\left[H_{2}O\right]}^{2}}$$ (13)

for the **1** + (H_2_O)_2_ reaction. The atmospheric lifetime *τ*_2_ is changed from 6.9 × 10^−5^ to 5.6 × 10^−2^ s as the altitude is increased from sea level to 10 km, whereas the atmospheric lifetimes of **1** with respect to R3.1 (*τ*_3.1_) and R4.1 (*τ*_4.1_) are even shorter: 3.1 × 10^−6^ s to 2.8 × 10^−3^ s and 3.8 × 10^−6^ s to 1.1 s at heights below 10 km. The atmospheric lifetime relative to isomerization (*τ*_1_) changes from 4.0 × 10^−2^ s to 4.0 × 10^1^ s over the 0- to 10-km range. This shows that **1** is mainly removed by **C-1** + H_2_O and **1** + (H_2_O)_2_.

In addition, the internal rotation channel may reinforce the pressure effects of the unimolecular reactions of **1** to further decrease the rate constant of unimolecular reactions [88]. However, the unimolecular reactions are not a major sink of **1**. Thus, we do not further consider this point.

## Conclusions

In this work, we use high-level coupled cluster theory and multistructural canonical VTST with small-curvature tunneling to investigate the unimolecular reactions of *E*-CH_2_(OH)CHOO (**1**) and the bimolecular reactions of **1** with H_2_O, of *E*-CH_2_(OH))CHOO…H_2_O (**C-1**) with H_2_O, and of **1** with (H_2_O)_2_. We find that the beyond-CCSD(T) contribution to the energetics depends on the specific reaction. The calculated results show that the introduction of the OH group enhances the reactivity of the Criegee intermediate.

The present findings help to elucidate the dominant sink of *E*-CH_2_(OH)CHOO (**1**) by water vapor. We find that **1** + (H_2_O)_2_ and **C-1** + H_2_O are the most important loss process of **1** in the atmosphere. In addition, the present findings show that **C-1** + H_2_O is more important than **1** + (H_2_O)_2_ at low temperatures.

Our investigation also shows that for R2, R3.1, and R4.1, anharmonicity is a key parameter for obtaining quantitative rate constants.

The results for R1a and R2.1 calculated in [62] are 2.4 and 6.1 times faster, respectively, than the values in our quantitative results at 298 K. These errors are all within the uncertainty range of the predictions as described in [62], thus confirming the expected qualitative reliability of the structure–activity relations (SARs) from [62]. Future application of SARs for qualitative prediction may be reasonable.

## Data Availability

Data are available in the Supplementary Materials.

## Supplementary Materials

Supplementary 1 Details of dual-level calculations of high-pressure-limit rate constants Details of the variable reaction coordinate rate calculations Table A1 Tables S1 to S28 Figs. S1 to S13
