# Supplementary material for: Unimolecular Reactions of E-Glycolaldehyde Oxide and Its Reactions with One and Two Water Molecules
Source: Research (Wash D C). 2023 Jul 10;6:0143. doi: 10.34133/research.0143 (PMC10332847; doi:10.34133/research.0143)
Supplement: Supplementary 1 — Details of dual-level calculations of high-pressure-limit rate constants Details of the variable reaction coordinate rate calculations Table A1 Tables S1 to S28 Figs. S1 to S13 [file research.0143.f1.pdf]

SUPPORTING INFORMATION
MARCH 21, 2023

Unimolecular reactions of *anti*-glycolaldehyde oxide and its reactions with one and two water molecules

Yan Sun,[a] Bo Long,*[a,b] and Donald G. Truhlar[c*]

[a]Department of Physics, Guizhou University, Guiyang, 550025, China

[b]College of Materials Science and Engineering, Guizhou Minzu University, Guiyang 550025, China

[c]Department of Chemistry, Chemical Theory Center, and Supercomputing Institute, University of Minnesota, Minneapolis, Minnesota 55455-0431, USA

TABLE OF CONTENTS

Details of dual-level calculations of high-pressure-limit rate constants S-3

Details of the variable-reaction-coordinate rate calculations S-5

Table A1. Electronic structure methods S-6

Table S1. Vibrational frequency scale factors S-7

Table S2. Reaction-specific scale factors S-7

Table S3. The relative energies (in kcal/mol) for the conformers of **1** S-8

Table S4. The change of zero-point vibrational energy for **1** + **H_2O** (in kcal mol^{-1} by various methods and mean unsigned deviations S-8

Table S5. The relative energies (in kcal/mol) for the conformers of **C-1** S-9

Table S6. Imaginary frequencies (in cm^{-1}) of the transition state structures by various theoretical methods for **C-1** + H_2O and mean unsigned deviations S-10

Table S7 The enthalpy of activation at 0 K using different scaling ways (in kcal/mol) S-10

Tables S8-S18. The tunneling and recrossing transmission coefficients, torsional anharmonicity factors, and rate constants (s^{-1}) in the high-pressure limit S-11

Table S19. The high-pressure limiting rate constants, $k_{3.1}$, from calculations on the loose ($k''_{3.1}$ for C-**1** + H_2O → **C-2**/**C-3**) and tight ($k'_{3.1}$ for C-**1** + H_2O → **5** + H_2O) transition states of reaction R3.1 (all rate constants in cm^3 molecule^{-1} s^{-1}) S-22

Table S20. The high-pressure limiting rate constants, $k_{4.1}$, from calculations on the loose ($k''_{4.1}$ for **1**+$(H_2O)_2$ → **C-2**/**C-3**) and tight ($k'_{4.1}$ for **1** + $(H_2O)_2$ → **5** + H_2O) transition states of reaction R4.1 (all rate constants in cm^3 molecule^{-1} s^{-1}) S-23

* Corresponding author email addresses:
wwwltcommon@sina.com (Bo Long)
truhlar@umn.edu (Donald G. Truhlar)

Table S21. The fitting parameters of rate constants for isomerization of **1** (k_1), **1** + H_2O (k_2), **C-1**+ H_2O ($k_{3.1}$), and **1** + $(H_2O)_2$ ($k_{4.1}$)	S-24
Table S22. Arrhenius Activation Energy (kcal/mol) for unimolecular reaction (E_{a1}), **1** + H_2O (E_{a2}), **C-1** + H_2O (E'_{a3}), and **1** + H_2O…H_2O (E''_{a3})	S-24
Table S23. The HPL tunneling transmission coefficient for **TS-2c**	S-25
Table S24. The temperature-pressure dependent rate constant $k(T,P)$ of reaction R1a as calculated by RRKM/ME with the higher level of electronic structure (W3X-L//Level-1)	S-26
Table S25. The temperature-and-pressure-dependent rate constant $k(T,P)$ of reaction R1a is calculated by SS-QRRK	S-27
Table S26. The transition pressure $P_{1/2}$	S-28
Table S27. Cartesian coordinates (Å)	S-29
Table S28. Absolute energies in hartrees	S-47
Figure S1. Enthalpy profile of the decay of product **5** of **1** + H_2O at 0 K by M06CR/MG3S (in kcal/mol)	S-49
Figure S2. The relative enthalpy profile at 0 K for R4.1 by W2X//Level-2, W3X-L//Level-2, and W2X+SC//Level-2 (in kcal/mol)	S-50
Figure S3. The relative enthalpy profile at 0 K for R3.2 by M06CR/MG3S (in kcal/mol)	S-51
Figure S4. The relative enthalpy profile at 0 K for R4.2 by M06CR/MG3S (in kcal/mol)	S-52
Figures S5-S13. The distinguishable structures of each species	S-53
References	S-61

Details of dual-level calculations of high-pressure-limit rate constants

The high-pressure-limit (HPL) bimolecular rate constants were calculated by the following dual-level expression:

$$k_{\mathrm{HPL}}^{\mathrm{DL}} = \frac{k_{\mathrm{TST}}^{\mathrm{SS-HL}}}{k_{\mathrm{TST}}^{\mathrm{SS-LL}}}\, k_{\mathrm{MS-CVT/SCT}}^{\mathrm{LL}} = F_{\mathrm{act}}\, \Gamma\kappa\, k_{\mathrm{TST}}^{\mathrm{SS-HL}} \quad (1)$$

where F_{act} is explained below, Γ and κ are described in detail in Section 2 of the article, and

$$k_{\mathrm{TST}} = \frac{k_{\mathrm{B}}T}{h} \frac{Q_{\mathrm{TS}}}{Q_{\mathrm{R1}} Q_{\mathrm{R2}} \Phi_{\mathrm{rel}}} \exp\left[-\frac{[H_0^{\mathrm{TS}} - (H_0^{\mathrm{R1}} + H_0^{\mathrm{R2}})]}{RT}\right] \quad (2)$$

where R1 and R2 are the reactants, TS is the transition state, TST is conventional transition state theory, k_{B}, h, and T are the Boltzmann constant, Planck constant, and temperature, respectively, Q is the partition function excluding translation, Φ_{rel} is the relative translational partition function per unit volume, and H_0^{TS} is the enthalpy of activation at 0 K, which equals the Born-Oppenheimer energy plus the zero-point vibrational energy. Because we include zero-point vibrational energy in the exponential, the partition functions must be computed with their zero of energy at the zero-point level (although in most of our papers, we write partition functions with the zero of energy at the equilibrium structure or the saddle point, and we put potential energy without the zero-point vibrational energy in the exponential). The quantity k_{TST}^{SS-HL} results from a calculation employing the lowest-energy conformers of the reactants and transition states and using the higher-level electronic structure method, whereas k_{TST}^{SS-LL} denotes the same kind of calculation but at the lower level of electronic structure. The product $F_{\mathrm{act}}\Gamma\kappa$ is computed at the lower level of electronic structure.

For the isomerization, the treatment is the same except that $Q_{\mathrm{R1}} Q_{\mathrm{R2}} \Phi_{\mathrm{rel}}$ is replaced by the partition function Q_{R} of a single reactant.

For the calculation of isomerization of **1** and its reaction with water monomer, the higher level is Level 3, and for the calculation of [**1** + H_2O...H_2O] and [**C-1** + H_2O], the higher level is Level 7.

F_{act} is the torsional anharmonicity factor for the reaction rate, and it is given by

$$F_{\mathrm{act}} = \frac{F_{\mathrm{TS}}^{\mathrm{MS}}}{F_{\mathrm{R}}^{\mathrm{MS}}} \quad (3)$$

where $F_{\mathrm{TS}}^{\mathrm{MS}}$ denotes the effect of torsional anharmonicity on the transition state, and F_R^{MS}

denotes the effect of torsional anharmonicity on the reactants (in the bimolecular case) or the reactant (in the unimolecular case). The quantities in eq 3 are calculated as the multi-structure anharmonic partition function divided by the single-structure quasi-harmonic partition function.

For isomerization of **1**, we used M11-L/MG3S to obtain F_{act}. For the other reactions, we used M06CR/MG3S to obtain F_{act}.

See Sections 3.1, 3.2, and 3.3 of the article for more details.

For R2.1, we ignore **1** + H_2O → **C-1**, and we use DL-MS-CVT/SCT to obtain k_2. For R3.1 and R4.1, both **C-1**+H_2O → pre-reaction complexes and **1** + $(H_2O)_2$ → pre-reaction complexes are significant, and we use the deep-intermediate limit of CUS theory to obtain $k_{3.1}$ and $k_{4.1}$ (see Tables S19 and S20).

Details of the variable-reaction-coordinate rate calculations for C-1 + H2O →C-2/C-3

We calculated this rate constant of **C-1** + **H_2O →C-2/C-3** by using variable-reaction-coordinate variational transition state theory (VRC-VTST) (1,2). We used two pivot points to produce a multi-faceted dividing surface. One pivot point is located at a distance d from the center of mass (COM) of **C-1**, where the vector connecting the pivot point with **C-1**'s COM is x axis of **C-1**, and the other pivot point is located at a distance d from the COM of H_2O, where the vector connecting the pivot point with H_2O's COM is perpendicular to the H_2O plane. The length of the two vectors were fixed at 0.05 Å. Additionally, we also tested changing the vector distances; however, the effect is negligible.

The reaction coordinate s is the distance between a pivot point on one reactant and the other pivot points on the other reactant. The distance s among pivot points was varied from 4.0 to 8.5 Å with a 0.1 Å grid increment to find the optimum value. We used 500 configurations for sampling the multi-faceted dividing surfaces.

Table A1. Electronic structure methods[a]

Abbreviation	Explanation
Level-1	CCSD(T)-F12a/cc-pVDZ-F12
Level-2	DF-CCSD(T)-F12b/jun-cc-pVDZ

[a] The levels defined here are the same as in Table 1 of the article proper, but the definitions of those involved in the SI are repeated here for convenience.

Table S1. Generic vibrational-frequency scale factors (λ^{ZPE}) obtained with the standard parametrization method

**Level**	**Generic scale factor (GSF)**
Level 1 (CCSD(T)-F12a/cc-pVDZ-F12	0.983
Level 2 (DF-CCSD(T)-F12b/jun-cc-pVDZ)	0.981
M06CR/MG3S	0.980
M11-L/MG3S	0.985
MN15-L/MG3S	0.977

Table S2. Reaction-specific scale factors (RSSFs)[a]

	**1**	**C-1**	$(H_2O)_2$	H_2O	**TS-1a**	**TS-2a**
ZPE (harmonic)	42.015	58.606	30.108	13.939	40.688	58.682
ZPE (anharmonic)	41.448	57.616	29.388	13.725	40.130	57.571
λ^{Anh}	0.987	0.983	0.976	0.985	0.986	0.981
Level-1 λ^{ZPE}	0.984			0.982	0.983	0.978
Level-2 λ^{ZPE}	0.982	0.978	0.971	0.980		
	**TS-2b**	**TS-3a**	**TS-3b**	**TS-3c**	**TS-3d**	
ZPE (harmonic)	57.903	74.575	74.043	74.126	74.411	
ZPE (anharmonic)	56.315	72.383	71.153	71.329	72.004	
λ^{Anh}	0.973	0.971	0.961	0.962	0.968	
Level-1 λ^{ZPE}	0.970					
Level-2 λ^{ZPE}		0.966	0.956	0.958	0.963	

[a] The harmonic and anharmonic zero-point vibrational energy are calculated by using the MPW1K/6-311 + G(2df,2p). The anharmonic scale factor is factor is

$$\lambda^{\mathrm{Anh}} = \frac{\mathrm{ZPE(anharm)}}{\mathrm{ZPE(harm)}}$$

The zero-point energy scale factor is

$$\lambda^{\mathrm{ZPE}} = \lambda^{\mathrm{H}}\lambda^{\mathrm{Anh}}$$

where $\lambda^{\mathrm{H}} = 0.997$ for Level 1, and $\lambda^{\mathrm{H}} = 0.995$ for Level-2.

Table S3. The relative energies (in kcal/mol) of conformers of **1** with respect to the lowest energy structure of **1**.

**1**	W2X//M06CR/MG3S	M06CR/MG3S
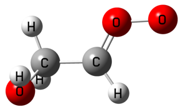	0.00	0.42
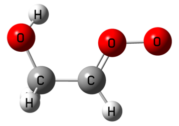	0.59	0.00
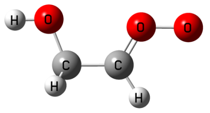	2.61	2.18
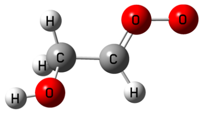	0.97	1.25
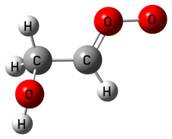	1.33	1.72

Table S4. The change of zero-point vibrational energy for reaction 2.1 (in kcal $mol^{-1}$by various methods and mean unsigned deviations from the best estimate

Method	$\Delta E^{\ddagger}_{\mathrm{ZPE}}$ [a] **TS-2a**	$\Delta E^{\ddagger}_{\mathrm{ZPE}}$ [a] **TS-2b**	MUD
Level-1	2.91	1.99	0.00
Level-2	2.88	2.03	0.04
MN15-L/MG3S	2.82	1.46	0.31
M06CR/MG3S	2.36	1.51	0.52
M11-L/MG3S	2.16	1.16	0.80

[a] $\Delta E^{\ddagger}_{\mathrm{ZPE}}$ denotes to the change of vibrational zero-point energies (E_{ZPE}) from the reactants to the transition states obtained by using the generic scale factor.

Table S5. The relative energies (in kcal/mol) for conformers of **C-1**

**C-1**	W2X//M06CR/MG3S	M06CR/MG3S
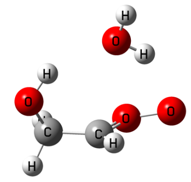	0.00	0.00
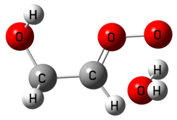	1.58	0.99
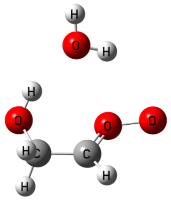	2.37	1.05
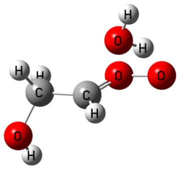	2.05	2.37
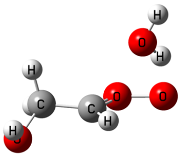	2.56	2.80
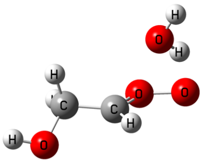	2.64	2.75

Table S6. Imaginary frequencies (in cm^{-1}) of transition stats structures and mean unsigned deviations from the best estimate

Method	**TS-3a**	**TS-3b**	**TS-3c**	**TS-3d**	MUD
Level-2	457i	548i	509i	486i	0.00
M06CR/MG3S	484i	608i	561i	529i	45i
MN15-L/MG3S	577i	874i	770i	653i	218i
M11-L/MG3S	666i	980i	922i	708i	319i

Table S7. Enthalpies of activation at 0 K (in kcal/mol)) by using a reaction-specific scale factor (RSSF) from Table S2 or by using a generic scale factor (GSF) from Table S1

Method	$\Delta H_0^{\ddagger}$			
**R3.1**	**TS-3a**	**TS-3b**	**TS-3c**	**TS-3d**
SC+W2X//Level2-RSSF	-4.20	-4.32	-4.11	-3.38
SC+W2X//Level2-GSF	-3.29	-2.71	-2.64	-2.27
**R4.1**	**TS-3a**	**TS-3b**	**TS-3c**	**TS-3d**
SC+W2X//Level2-RSSF	-10.08	-10.20	-9.99	-9.27
SC+W2X//Level2-GSF	-9.25	-8.67	-8.60	-8.23
**R2a**	**TS-2a**	**TS-2b**		
W3X-L//Level1-RSSF	-0.41	-0.15		
W3X-L//Level1-GSF	-0.09	0.60		
**R1a**	**TS-1a**			
W3X-L//Level1-RSSF	15.15			
W3X-L//Level1-GSF	15.19			

Table S8. The tunneling and recrossing coefficient, torsional anharmonic factor and HPL rate constant (s^{-1}) of the ring-closure reaction R1a[a]

T/K	$F_{act}^{MS\,a}$	$k_{TST}^{RSSF\,b}$	$\Gamma_{CVT}^{LL\,c}$	$\kappa_{SCT}^{LL\,c}$	$k_{HPL}^{DL\,d}$	$k_{TST}^{GSF\,e}$	$f^{\,f}$
190	1.50E+00	8.90E-06	9.92E-1	1.82	2.41E-5	8.01E-06	1.11
200	1.53E+00	6.90E-05	9.92E-1	1.70	1.79E-4	6.24E-05	1.11
210	1.56E+00	4.43E-04	9.92E-1	1.61	1.10E-3	4.03E-04	1.10
220	1.59E+00	2.40E-03	9.92E-1	1.54	5.81E-3	2.19E-03	1.10
230	1.62E+00	1.12E-02	9.92E-1	1.48	2.66E-2	1.03E-02	1.09
240	1.65E+00	4.62E-02	9.92E-1	1.43	1.08E-1	4.25E-02	1.09
250	1.68E+00	1.70E-01	9.91E-1	1.38	3.92E-1	1.57E-01	1.08
260	1.71E+00	5.65E-01	9.91E-1	1.35	1.29	5.23E-01	1.08
270	1.74E+00	1.73E+00	9.91E-1	1.32	3.92	1.61E+00	1.08
280	1.76E+00	4.87E+00	9.91E-1	1.29	1.10E+1	4.53E+00	1.07
290	1.79E+00	1.28E+01	9.91E-1	1.27	2.87E+1	1.19E+01	1.07
298	1.81E+00	2.65E+01	9.91E-1	1.25	5.93E+1	2.48E+01	1.07
300	1.81E+00	3.15E+01	9.91E-1	1.25	7.06E+1	2.95E+01	1.07
310	1.84E+00	7.35E+01	9.91E-1	1.23	1.64E+2	6.89E+01	1.07
320	1.86E+00	1.62E+02	9.91E-1	1.21	3.62E+2	1.52E+02	1.07
330	1.88E+00	3.41E+02	9.91E-1	1.20	7.61E+2	3.21E+02	1.06
340	1.90E+00	6.85E+02	9.90E-1	1.19	1.53E+3	6.46E+02	1.06
350	1.92E+00	1.33E+03	9.90E-1	1.17	2.97E+3	1.26E+03	1.06

[a] F_{act}^{MS} denotes the effect of torsional anharmonicity on a given reaction path. It was calculated by the lower-level electron structure method (M11-L/MG3S) with the generic scale factor. For this reaction path, five distinguishable conformers are included for reactants (see Figure S5), and five conformers are included for transition states (see Figure S6).

[b] The rate constants (k_{TST}^{RSSF}) calculated by conventional transition state theory based on the higher-level electron structure method (W3X-L//Level-1) with the reaction-specific scale factor

[c] The recrossing transmission coefficient and tunneling transmission was calculated based on the lower-level electron structure method (M11-L/MG3S) with generic scale factors.

[d] The rate constant (k_{HPL}^{DL}) in the high-pressure limit condition as obtained by the dual-level strategy ($k_{HPL}^{DL} = F_{act}^{MS} k_{TST}^{RSSF} \Gamma_{CVT}^{LL} \kappa_{SCT}^{LL}$)

[e] The rate constants calculated by conventional transition state theory based on the higher-level electron structure method (W3X-L//Level-1) with the generic scale factor

[f] $f = k_{TST}^{RSSF}/k_{TST}^{GSF}$, which represents the effects of scale factors using different methods on the rate constant

Table S9. The tunneling and recrossing transmission coefficients, the torsional anharmonic factors, and HPL rate constants (cm^3 $molecule^{-1}$ s^{-1}) for passage through **TS-2a**[a]

T/K	F_{act}^{MS}	k_{TST}^{RSSF} [b]	Γ_{CVT}^{LL}	κ_{SCT}^{LL}	k_{HPL}^{DL} [c]	k_{TST}^{GSF} [d]	f [e]
190	6.51E-01	5.05E-14	9.36E-1	1.67	5.13E-14	2.20E-14	2.30
200	6.43E-01	4.46E-14	9.42E-1	1.58	4.24E-14	2.02E-14	2.21
210	6.36E-01	3.98E-14	9.47E-1	1.51	3.62E-14	1.87E-14	2.13
220	6.30E-01	3.59E-14	9.51E-1	1.45	3.12E-14	1.74E-14	2.06
230	6.24E-01	3.26E-14	9.56E-1	1.40	2.73E-14	1.63E-14	2.00
240	6.20E-01	2.99E-14	9.60E-1	1.36	2.41E-14	1.54E-14	1.95
250	6.17E-01	2.76E-14	9.63E-1	1.32	2.18E-14	1.45E-14	1.90
260	6.13E-01	2.56E-14	9.67E-1	1.30	1.96E-14	1.38E-14	1.86
270	6.12E-01	2.39E-14	9.70E-1	1.27	1.80E-14	1.32E-14	1.82
280	6.10E-01	2.25E-14	9.73E-1	1.25	1.66E-14	1.26E-14	1.79
290	6.08E-01	2.12E-14	9.76E-1	1.23	1.55E-14	1.21E-14	1.75
298	6.08E-01	2.03E-14	9.78E-1	1.21	1.47E-14	1.18E-14	1.73
300	6.07E-01	2.01E-14	9.78E-1	1.21	1.44E-14	1.17E-14	1.72
310	6.07E-01	1.91E-14	9.80E-1	1.20	1.36E-14	1.13E-14	1.69
320	6.07E-01	1.83E-14	9.83E-1	1.18	1.29E-14	1.10E-14	1.67
330	6.07E-01	1.76E-14	9.85E-1	1.17	1.23E-14	1.07E-14	1.65
340	6.08E-01	1.69E-14	9.86E-1	1.16	1.17E-14	1.04E-14	1.63
350	6.09E-01	1.63E-14	9.88E-1	1.15	1.13E-14	1.02E-14	1.61

[a]The recrossing transmission coefficients, the tunneling transmission coefficients, and the torsional anharmonic factors were calculated by the lower-level electron structure method with a generic scale factor. For this reaction path, five distinguishable conformers are included for reactants (see Figure S5), and five conformers are included for transition state (see Figure S7).

[b] k_{TST}^{RSSF} comes from calculations using the higher-level electronic structure method (W3X-L//Level-1) with the reaction-specific scale factor.

[c] The rate constant (k_{HPL}^{DL}) in the high-pressure limit condition as obtained by the dual-level strategy ($k_{HPL}^{DL} = F_{act}^{MS} k_{TST}^{RSSF} \Gamma_{CVT}^{LL} \kappa_{SCT}^{LL}$)

[d] The rate constants (k_{TST}^{GSF}) calculated by conventional transition state theory based on the higher-level electron structure method (W3X-L//Level-1) with the generic scale factor

[e] $f = k_{TST}^{RSSF}/k_{TST}^{GSF}$, which represents the effects of scale factors using different methods on the rate constant

Table S10. The tunneling and recrossing transmission coefficients, the torsional anharmonic factors, and HPL rate constants (cm^3 $molecule^{-1}$ s^{-1}) for passage through **TS-2b**[a]

T/K	F_{act}^{MS}	$k_{TST}^{RSSF\ b}$	Γ_{CVT}^{LL}	κ_{SCT}^{LL}	$k_{HPL}^{DL\ c}$	$k_{TST}^{GSF\ d}$	f^e
190	1.31E+00	2.55E-14	5.48E-1	3.84	7.04E-14	3.37E-15	7.58
200	1.28E+00	2.31E-14	5.67E-1	3.28	5.51E-14	3.37E-15	6.86
210	1.26E+00	2.11E-14	5.85E-1	2.88	4.49E-14	3.36E-15	6.29
220	1.24E+00	1.94E-14	6.02E-1	2.59	3.76E-14	3.35E-15	5.79
230	1.22E+00	1.80E-14	6.19E-1	2.36	3.22E-14	3.34E-15	5.39
240	1.21E+00	1.68E-14	6.34E-1	2.18	2.81E-14	3.33E-15	5.05
250	1.20E+00	1.58E-14	6.48E-1	2.04	2.50E-14	3.32E-15	4.77
260	1.19E+00	1.48E-14	6.62E-1	1.92	2.24E-14	3.31E-15	4.48
270	1.18E+00	1.41E-14	6.74E-1	1.82	2.05E-14	3.30E-15	4.28
280	1.18E+00	1.34E-14	6.87E-1	1.74	1.89E-14	3.29E-15	4.08
290	1.17E+00	1.28E-14	6.98E-1	1.67	1.75E-14	3.28E-15	3.90
298	1.17E+00	1.23E-14	7.07E-1	1.62	1.65E-14	3.28E-15	3.76
300	1.17E+00	1.22E-14	7.09E-1	1.61	1.62E-14	3.28E-15	3.73
310	1.16E+00	1.17E-14	7.20E-1	1.56	1.53E-14	3.27E-15	3.58
320	1.16E+00	1.13E-14	7.30E-1	1.52	1.46E-14	3.27E-15	3.46
330	1.16E+00	1.10E-14	7.39E-1	1.48	1.38E-14	3.27E-15	3.36
340	1.16E+00	1.06E-14	7.48E-1	1.44	1.33E-14	3.27E-15	3.24
350	1.15E+00	1.03E-14	7.56E-1	1.41	1.27E-14	3.28E-15	3.15

[a]The recrossing transmission coefficients, the tunneling transmission coefficients, and the torsional anharmonic factors were calculated by the lower-level electron structure method with generic scale factors. For this reaction path, five distinguishable conformers are included for reactants (see Figure S5), and six conformers are included for transition state (see Figure S8).

[b] k_{TST}^{RSSF} comes from calculations using the higher-level electronic structure method (W3X-L//Level-1) with the reaction-specific scale factor.

[c] The rate constant (k_{HPL}^{DL}) in the high-pressure limit condition as obtained by the dual-level strategy ($k_{HPL}^{DL} = F_{act}^{MS} k_{TST}^{RSSF} \Gamma_{CVT}^{LL} \kappa_{SCT}^{LL}$)

[d]The rate constants (k_{TST}^{GSF}) calculated by conventional transition state theory based on the higher-level electron structure method (W3X-L//Level-1) with the generic scale factor

[e]$f = k_{TST}^{RSSF}/k_{TST}^{GSF}$, which represents the effects of scale factors using different methods on the rate constant

Table S11. The tunneling and recrossing transmission coefficients, the torsional anharmonic factors, and HPL rate constants (cm^3 $molecule^{-1}$ s^{-1}) for **C-1** + H_2O passage through **TS-3a**[a]

T/K	F_{act}^{MS}	k_{TST}^{RSSF} [b]	Γ_{CVT}^{LL}	κ_{SCT}^{LL}	k_{HPL}^{DL} [c]	k_{TST}^{GSF} [d]	f [e]
190	9.42E-01	4.48E-10	3.07E-1	1.92E+2	2.49E-08	3.94E-11	11.39
200	9.00E-01	2.29E-10	3.30E-1	8.07E+1	5.50E-09	2.26E-11	10.16
210	8.61E-01	1.24E-10	3.54E-1	3.78E+1	1.43E-09	1.36E-11	9.15
220	8.25E-01	7.05E-11	3.76E-1	1.97E+1	4.31E-10	8.50E-12	8.29
230	7.90E-01	4.22E-11	3.98E-1	1.13E+1	1.50E-10	5.55E-12	7.60
240	7.58E-01	2.63E-11	4.19E-1	7.17	5.98E-11	3.74E-12	7.03
250	7.27E-01	1.70E-11	4.39E-1	4.95	2.68E-11	2.60E-12	6.55
260	7.00E-01	1.13E-11	4.59E-1	3.69	1.34E-11	1.85E-12	6.11
270	6.74E-01	7.75E-12	4.79E-1	2.93	7.31E-12	1.35E-12	5.74
280	6.50E-01	5.45E-12	4.97E-1	2.45	4.32E-12	1.01E-12	5.42
290	6.27E-01	3.94E-12	5.16E-1	2.13	2.72E-12	7.65E-13	5.15
298	6.11E-01	3.08E-12	5.30E-1	1.95	1.94E-12	6.20E-13	4.97
300	6.07E-01	2.90E-12	5.33E-1	1.92	1.80E-12	5.90E-13	4.92
310	5.87E-01	2.18E-12	5.50E-1	1.76	1.24E-12	4.65E-13	4.69
320	5.70E-01	1.66E-12	5.66E-1	1.64	8.80E-13	3.71E-13	4.48
330	5.53E-01	1.29E-12	5.82E-1	1.56	6.45E-13	3.00E-13	4.31
340	5.37E-01	1.02E-12	5.97E-1	1.49	4.87E-13	2.45E-13	4.16
350	5.23E-01	8.10E-13	6.12E-1	1.43	3.71E-13	2.03E-13	4.00

[a] The recrossing transmission coefficients, the tunneling transmission coefficients, and the torsional anharmonic factors were calculated by the lower-level electron structure method with generic scale factors. For this reaction path, six distinguishable conformers are included for reactants (see Figure S9), and seven conformers are included for transition states (see Figure S10).

[b] k_{TST}^{RSSF} comes from calculations using the higher-level electronic structure method (W3X-L//Level-2) with the reaction-specific scale factor.

[c] The rate constant (k_{HPL}^{DL}) in the high-pressure limit condition as obtained by the dual-level strategy ($k_{HPL}^{DL} = F_{act}^{MS} k_{TST}^{RSSF} \Gamma_{CVT}^{LL} \kappa_{SCT}^{LL}$)

[d] The rate constants (k_{TST}^{GSF}) calculated by conventional transition state theory based on the higher-level electron structure method (W3X-L//Level-2) with the generic scale factor

[e] $f = k_{TST}^{RSSF}/k_{TST}^{GSF}$, which represents the effects of scale factors using different methods on the rate constant

Table S12. The tunneling and recrossing transmission coefficients, the torsional anharmonic factors, and HPL rate constants (cm^3 $molecule^{-1}$ s^{-1}) for **C-1** + H_2O passage through **TS-3b**[a]

T/K	F_{act}^{MS}	$k_{TST}^{RSSF\ b}$	Γ_{CVT}^{LL}	κ_{SCT}^{LL}	$k_{HPL}^{DL\ c}$	$k_{TST}^{GSF\ d}$	f^e
190	1.27E+00	8.40E-10	2.27E-1	9.85E+2	2.39E-07	1.09E-11	77.06
200	1.23E+00	4.25E-10	2.50E-1	3.70E+2	4.82E-08	6.80E-12	62.50
210	1.18E+00	2.29E-10	2.73E-1	1.55E+2	1.14E-08	4.40E-12	52.10
220	1.13E+00	1.30E-10	2.95E-1	7.19E+1	3.12E-09	2.96E-12	43.99
230	1.09E+00	7.70E-11	3.18E-1	3.66E+1	9.71E-10	2.05E-12	37.56
240	1.04E+00	4.77E-11	3.39E-1	2.04E+1	3.44E-10	1.46E-12	32.67
250	9.97E-01	3.07E-11	3.61E-1	1.23E+1	1.36E-10	1.07E-12	28.69
260	9.56E-01	2.04E-11	3.82E-1	8.07	6.00E-11	8.00E-13	25.50
270	9.15E-01	1.39E-11	4.02E-1	5.68	2.90E-11	6.10E-13	22.79
280	8.77E-01	9.75E-12	4.22E-1	4.27	1.54E-11	4.75E-13	20.55
290	8.40E-01	7.00E-12	4.41E-1	3.40	8.82E-12	3.75E-13	18.67
298	8.13E-01	5.45E-12	4.56E-1	2.92	5.89E-12	3.14E-13	17.36
300	8.06E-01	5.15E-12	4.60E-1	2.83	5.40E-12	3.01E-13	17.14
310	7.75E-01	3.86E-12	4.78E-1	2.44	3.50E-12	2.45E-13	15.79
320	7.45E-01	2.94E-12	4.95E-1	2.17	2.34E-12	2.01E-13	14.63
330	7.17E-01	2.28E-12	5.12E-1	1.97	1.65E-12	1.68E-13	13.61
340	6.92E-01	1.80E-12	5.28E-1	1.83	1.20E-12	1.41E-13	12.77
350	6.67E-01	1.43E-12	5.44E-1	1.72	8.91E-13	1.20E-13	11.97

[a] The recrossing transmission coefficients, the tunneling transmission coefficients, and the torsional anharmonic factors were calculated by the lower-level electron structure method with generic scale factors. For this reaction path, six distinguishable conformers are included for reactants (see Figure S9), and six conformers are included for transition states (see Figure S11).

[b] k_{TST}^{RSSF} comes from calculations using the higher-level electronic structure method (W3X-L//Level-2) with the reaction-specific scale factor.

[c] The rate constant (k_{HPL}^{DL}) in the high-pressure limit condition as obtained by the dual-level strategy ($k_{HPL}^{DL} = F_{act}^{MS} k_{TST}^{RSSF} \Gamma_{CVT}^{LL} \kappa_{SCT}^{LL}$)

[d] The rate constants (k_{TST}^{GSF}) calculated by conventional transition state theory based on the higher-level electron structure method (W3X-L//Level-2) with the generic scale factor

[e] $f = k_{TST}^{RSSF} / k_{TST}^{GSF}$, which represents the effects of scale factors using different methods on the rate constant

Table S13. The tunneling and recrossing transmission coefficients, the torsional anharmonic factors, and HPL rate constants (cm^3 $molecule^{-1}$ s^{-1}) for **C-1** + H_2O passage through **TS-3c**[a]

T/K	F_{act}^{MS}	$k_{TST}^{RSSF\ b}$	Γ_{CVT}^{LL}	κ_{SCT}^{LL}	$k_{HPL}^{DL\ c}$	$k_{TST}^{GSF\ d}$	f^{e}
190	1.06E+00	4.49E-10	2.80E-1	1.28E+4	1.70E-06	8.45E-12	53.14
200	1.03E+00	2.33E-10	3.05E-1	3.34E+3	2.44E-07	5.30E-12	43.96
210	9.94E-01	1.29E-10	3.30E-1	1.01E+3	4.27E-08	3.48E-12	37.07
220	9.61E-01	7.45E-11	3.54E-1	3.44E+2	8.74E-09	2.36E-12	31.57
230	9.27E-01	4.53E-11	3.78E-1	1.32E+2	2.09E-09	1.65E-12	27.46
240	8.95E-01	2.86E-11	4.01E-1	5.63E+1	5.78E-10	1.19E-12	24.14
250	8.61E-01	1.87E-11	4.24E-1	2.67E+1	1.82E-10	8.70E-13	21.49
260	8.30E-01	1.26E-11	4.46E-1	1.41E+1	6.57E-11	6.55E-13	19.24
270	7.98E-01	8.75E-12	4.67E-1	8.25	2.69E-11	5.05E-13	17.33
280	7.69E-01	6.25E-12	4.88E-1	5.36	1.25E-11	3.94E-13	15.88
290	7.40E-01	4.54E-12	5.08E-1	3.82	6.52E-12	3.13E-13	14.53
298	7.20E-01	3.57E-12	5.23E-1	3.09	4.16E-12	2.63E-13	13.60
300	7.14E-01	3.37E-12	5.27E-1	2.95	3.75E-12	2.52E-13	13.40
310	6.89E-01	2.56E-12	5.46E-1	2.43	2.35E-12	2.06E-13	12.46
320	6.66E-01	1.97E-12	5.64E-1	2.11	1.56E-12	1.70E-13	11.62
330	6.43E-01	1.54E-12	5.81E-1	1.89	1.09E-12	1.42E-13	10.85
340	6.22E-01	1.23E-12	5.97E-1	1.73	7.96E-13	1.20E-13	10.25
350	6.03E-01	9.85E-13	6.13E-1	1.62	5.91E-13	1.02E-13	9.66

[a] The recrossing transmission coefficients, the tunneling transmission coefficients, and the torsional anharmonic factors were calculated by the lower-level electron structure method by using the generic scale factor. For this reaction path, six distinguishable conformers are included for reactants (see Figure S9), and six structures are included for transition states (see Figure S12).

[b] k_{TST}^{RSSF} comes from calculations using the higher-level electronic structure method (W3X-L//Level-2) with the reaction-specific scale factor.

[c] The rate constant (k_{HPL}^{DL}) in the high-pressure limit condition as obtained by the dual-level strategy ($k_{HPL}^{DL} = F_{act}^{MS} k_{TST}^{RSSF} \Gamma_{CVT}^{LL} \kappa_{SCT}^{LL}$)

[d] The rate constants (k_{TST}^{GSF}) calculated by conventional transition state theory based on the higher-level electron structure method (W3X-L//Level-2) with the generic scale factor

[e] $f = k_{TST}^{RSSF} / k_{TST}^{GSF}$, which represents the effects of scale factors using different methods on the rate constant

Table S14. The tunneling and recrossing transmission coefficients, the torsional anharmonic factors, and HPL rate constants (cm^3 molecule^{-1} s^{-1}) for **C-1** + H_2O passage through **TS-3d**[a]

T/K	$F_{\mathrm{act}}^{\mathrm{MS}}$	$k_{\mathrm{TST}}^{\mathrm{RSSF}\ b}$	$\Gamma_{\mathrm{CVT}}^{\mathrm{LL}}$	$\kappa_{\mathrm{SCT}}^{\mathrm{LL}}$	$k_{\mathrm{HPL}}^{\mathrm{DL}\ c}$	$k_{\mathrm{TST}}^{\mathrm{GSF}d}$	f^e
190	9.72E-01	5.40E-11	2.59E-1	3.29E+4	1.16E-07	2.65E-12	20.38
200	9.57E-01	3.06E-11	2.81E-1	8.06E+3	1.87E-08	1.74E-12	17.59
210	9.39E-01	1.83E-11	3.04E-1	2.29E+3	3.63E-09	1.19E-12	15.44
220	9.23E-01	1.14E-11	3.26E-1	7.37E+2	8.23E-10	8.35E-13	13.65
230	9.06E-01	7.40E-12	3.47E-1	2.66E+2	2.15E-10	6.00E-13	12.33
240	8.89E-01	4.97E-12	3.68E-1	1.07E+2	6.39E-11	4.45E-13	11.17
250	8.71E-01	3.44E-12	3.88E-1	4.75E+1	2.14E-11	3.37E-13	10.22
260	8.56E-01	2.44E-12	4.08E-1	2.33E+1	8.10E-12	2.60E-13	9.39
270	8.40E-01	1.78E-12	4.28E-1	1.26E+1	3.45E-12	2.04E-13	8.73
280	8.25E-01	1.32E-12	4.47E-1	7.54	1.64E-12	1.63E-13	8.10
290	8.11E-01	1.00E-12	4.65E-1	4.98	8.71E-13	1.32E-13	7.58
298	7.99E-01	8.15E-13	4.79E-1	3.81	5.71E-13	1.13E-13	7.24
300	7.97E-01	7.75E-13	4.83E-1	3.59	5.19E-13	1.09E-13	7.14
310	7.83E-01	6.05E-13	5.00E-1	2.80	3.32E-13	9.00E-14	6.72
320	7.71E-01	4.83E-13	5.17E-1	2.32	2.31E-13	7.55E-14	6.40
330	7.59E-01	3.90E-13	5.33E-1	2.02	1.70E-13	6.40E-14	6.09
340	7.48E-01	3.19E-13	5.49E-1	1.82	1.31E-13	5.50E-14	5.8
350	7.37E-01	2.64E-13	5.64E-1	1.68	1.04E-13	4.74E-14	5.57

[a] The recrossing transmission coefficients, the tunneling transmission coefficients, and the torsional anharmonic factors were calculated by the lower-level electron structure method by using the generic scale factor. For this reaction path, six distinguishable conformers are included for reactants (see Figure S9), and seven structures are included for transition states (see Figure S13).

[b] $k_{\mathrm{TST}}^{\mathrm{RSSF}}$ comes from calculations using the higher-level electronic structure method (W3X-L//Level-2) with the reaction-specific scale factor.

[c] The rate constant ($k_{\mathrm{HPL}}^{\mathrm{DL}}$) in the high-pressure limit condition as obtained by the dual-level strategy ($k_{\mathrm{HPL}}^{\mathrm{DL}} = F_{\mathrm{act}}^{\mathrm{MS}} k_{\mathrm{TST}}^{\mathrm{RSSF}} \Gamma_{\mathrm{CVT}}^{\mathrm{LL}} \kappa_{\mathrm{SCT}}^{\mathrm{LL}}$)

[d] The rate constants ($k_{\mathrm{TST}}^{\mathrm{GSF}}$) calculated by conventional transition state theory based on the higher-level electron structure method (W3X-L//Level-2) with the generic scale factor

[e] $f = k_{\mathrm{TST}}^{\mathrm{RSSF}} / k_{\mathrm{TST}}^{\mathrm{GSF}}$, which represents the effects of scale factors using different methods on the rate constant

Table S15. The tunneling and recrossing transmission coefficients, the torsional anharmonic factors, and HPL rate constants (cm^3 $molecule^{-1}$ s^{-1}) for **1** + $(H_2O)_2$ passage through **TS-3a**[a]

T/K	F_{act}^{MS}	k_{TST}^{RSSF} [b]	Γ_{CVT}^{LL}	κ_{SCT}^{LL}	k_{HPL}^{DL} [c]	k_{TST}^{GSF} [d]	f [e]
190	5.05E-01	1.26E-05	3.07E-1	1.92E+2	3.76E-04	1.34E-06	9.44
200	4.92E-01	2.89E-06	3.30E-1	8.07E+1	3.79E-05	3.43E-07	8.44
210	4.82E-01	7.60E-07	3.54E-1	3.78E+1	4.90E-06	9.90E-08	7.68
220	4.74E-01	2.25E-07	3.76E-1	1.97E+1	7.89E-07	3.20E-08	7.03
230	4.67E-01	7.35E-08	3.98E-1	1.13E+1	1.55E-07	1.14E-08	6.46
240	4.61E-01	2.64E-08	4.19E-1	7.17	3.65E-08	4.40E-09	6.00
250	4.58E-01	1.03E-08	4.39E-1	4.95	1.02E-08	1.82E-09	5.65
260	4.55E-01	4.27E-09	4.59E-1	3.69	3.28E-09	8.08E-10	5.29
270	4.54E-01	1.90E-09	4.79E-1	2.93	1.21E-09	3.80E-10	5.00
280	4.53E-01	8.90E-10	4.97E-1	2.45	4.91E-10	1.88E-10	4.75
290	4.52E-01	4.40E-10	5.16E-1	2.13	2.19E-10	9.73E-11	4.52
298	4.52E-01	2.59E-10	5.30E-1	1.95	1.21E-10	5.93E-11	4.37
300	4.53E-01	2.27E-10	5.33E-1	1.92	1.05E-10	5.28E-11	4.30
310	4.53E-01	1.23E-10	5.50E-1	1.76	5.39E-11	2.95E-11	4.17
320	4.55E-01	6.85E-11	5.66E-1	1.64	2.90E-11	1.73E-11	3.97
330	4.56E-01	3.98E-11	5.82E-1	1.56	1.64E-11	1.04E-11	3.84
340	4.58E-01	2.38E-11	5.97E-1	1.49	9.69E-12	6.43E-12	3.70
350	4.60E-01	1.47E-11	6.12E-1	1.43	5.93E-12	4.08E-12	3.61

[a] The recrossing transmission coefficients, the tunneling transmission coefficients, and the torsional anharmonic factors were calculated by the lower-level electron structure method with the generic scale factors. For this reaction path, five distinguishable structures are included for reactants (see Figure S5), and seven structures are included for transition states (see Figure S10).

[b] k_{TST}^{RSSF} comes from calculations using the higher-level electronic structure method (W3X-L//Level-2) with the reaction-specific scale factor.

[c] The rate constant (k_{HPL}^{DL}) in the high-pressure limit condition as obtained by the dual-level strategy ($k_{HPL}^{DL} = F_{act}^{MS} k_{TST}^{RSSF} \Gamma_{CVT}^{LL} \kappa_{SCT}^{LL}$)

[d] The rate constants (k_{TST}^{GSF}) calculated by conventional transition state theory based on the higher-level electron structure method (W3X-L//Level-2) with the generic scale factor

[e] $f = k_{TST}^{RSSF}/k_{TST}^{GSF}$, which represents the effects of scale factors using different methods on the rate constant

Table S16. The tunneling and recrossing transmission coefficients, the torsional anharmonic factors, and HPL rate constants (cm^3 $molecule^{-1}$ s^{-1}) for **1** + $(H_2O)_2$ passage through **TS-3b**[a]

T/K	F_{act}^{MS}	$k_{TST}^{RSSF\ b}$	Γ_{CVT}^{LL}	κ_{SCT}^{LL}	$k_{HPL}^{DL\ c}$	$k_{TST}^{GSF\ d}$	f^e
190	6.81E-01	2.36E-05	2.27E-1	9.85E+2	3.60E-03	3.70E-07	63.78
200	6.70E-01	5.35E-06	2.50E-1	3.70E+2	3.31E-04	1.03E-07	51.94
210	6.60E-01	1.40E-06	2.73E-1	1.55E+2	3.91E-05	3.23E-08	43.41
220	6.51E-01	4.12E-07	2.95E-1	7.19E+1	5.69E-06	1.11E-08	37.03
230	6.43E-01	1.35E-07	3.18E-1	3.66E+1	1.01E-06	4.20E-09	32.14
240	6.35E-01	4.79E-08	3.39E-1	2.04E+1	2.10E-07	1.72E-09	27.93
250	6.28E-01	1.85E-08	3.61E-1	1.23E+1	5.16E-08	7.50E-10	24.67
260	6.21E-01	7.70E-09	3.82E-1	8.07	1.47E-08	3.50E-10	22
270	6.15E-01	3.40E-09	4.02E-1	5.68	4.77E-09	1.72E-10	19.83
280	6.10E-01	1.59E-09	4.22E-1	4.27	1.75E-09	8.85E-11	17.97
290	6.05E-01	7.85E-10	4.41E-1	3.40	7.13E-10	4.78E-11	16.44
298	6.02E-01	4.60E-10	4.56E-1	2.92	3.68E-10	3.00E-11	15.33
300	6.02E-01	4.04E-10	4.60E-1	2.83	3.16E-10	2.68E-11	15.10
310	5.98E-01	2.17E-10	4.78E-1	2.44	1.52E-10	1.56E-11	13.96
320	5.95E-01	1.22E-10	4.95E-1	2.17	7.76E-11	9.35E-12	13.05
330	5.92E-01	7.00E-11	5.12E-1	1.97	4.19E-11	5.80E-12	12.09
340	5.90E-01	4.20E-11	5.28E-1	1.83	2.39E-11	3.70E-12	11.35
350	5.88E-01	2.58E-11	5.44E-1	1.72	1.42E-11	2.41E-12	10.69

[a] The recrossing transmission coefficients, the tunneling transmission coefficients, and the torsional anharmonic factors were calculated by the lower-level electron structure method with the generic scale factors. For this reaction path, five distinguishable conformers are included for reactants (see Figure S5), and six conformers are included for transition states (see Figure S11).

[b] k_{TST}^{RSSF} comes from calculations using the higher-level electronic structure method (W3X-L//Level-2) with the reaction-specific scale factor.

[c] The rate constant (k_{HPL}^{DL}) in the high-pressure limit condition as obtained by the dual-level strategy ($k_{HPL}^{DL} = F_{act}^{MS} k_{TST}^{RSSF} \Gamma_{CVT}^{LL} \kappa_{SCT}^{LL}$)

[d] The rate constants (k_{TST}^{GSF}) calculated by conventional transition state theory based on the higher-level electron structure method (W3X-L//Level-2) with the generic scale factor

[e] $f = k_{TST}^{RSSF}/k_{TST}^{GSF}$, which represents the effects of scale factors using different methods on the rate constant

Table S17. The tunneling and recrossing transmission coefficients, the torsional anharmonic factors, and HPL rate constants (cm^3 molecule^{-1} s^{-1}) for **1** + $(H_2O)_2$ passage through **TS-3c**[a]

T/K	$F_{\mathrm{act}}^{\mathrm{MS}}$	$k_{\mathrm{TST}}^{\mathrm{RSSF}\ b}$	$\Gamma_{\mathrm{CVT}}^{\mathrm{LL}}$	$\kappa_{\mathrm{SCT}}^{\mathrm{LL}}$	$k_{\mathrm{HPL}}^{\mathrm{DL}\ c}$	$k_{\mathrm{TST}}^{\mathrm{GSF}d}$	f^e
190	5.66E-01	1.26E-05	2.80E-1	1.28E+4	2.55E-02	2.88E-07	43.83
200	5.61E-01	2.95E-06	3.05E-1	3.34E+3	1.69E-03	8.08E-08	36.53
210	5.56E-01	7.90E-07	3.30E-1	1.01E+3	1.46E-04	2.55E-08	30.98
220	5.52E-01	2.37E-07	3.54E-1	3.44E+2	1.60E-05	8.88E-09	26.70
230	5.48E-01	7.90E-08	3.78E-1	1.32E+2	2.16E-06	3.38E-09	23.41
240	5.45E-01	2.87E-08	4.01E-1	5.63E+1	3.53E-07	1.39E-09	20.65
250	5.42E-01	1.13E-08	4.24E-1	2.67E+1	6.92E-08	6.13E-10	18.45
260	5.39E-01	4.76E-09	4.46E-1	1.41E+1	1.61E-08	2.88E-10	16.56
270	5.37E-01	2.14E-09	4.67E-1	8.25	4.43E-09	1.42E-10	15.12
280	5.35E-01	1.02E-09	4.88E-1	5.36	1.43E-09	7.33E-11	13.93
290	5.33E-01	5.05E-10	5.08E-1	3.82	5.22E-10	3.98E-11	12.70
298	5.33E-01	3.00E-10	5.23E-1	3.09	2.59E-10	2.50E-11	12
300	5.33E-01	2.65E-10	5.27E-1	2.95	2.20E-10	2.24E-11	11.83
310	5.31E-01	1.44E-10	5.46E-1	2.43	1.02E-10	1.31E-11	11.013
320	5.31E-01	8.10E-11	5.64E-1	2.11	5.12E-11	7.90E-12	10.25
330	5.31E-01	4.75E-11	5.81E-1	1.89	2.77E-11	4.93E-12	9.64
340	5.31E-01	2.87E-11	5.97E-1	1.73	1.58E-11	3.15E-12	9.11
350	5.31E-01	1.78E-11	6.13E-1	1.62	9.40E-12	2.06E-12	8.65

[a] The recrossing transmission coefficients, the tunneling transmission coefficients, and the torsional anharmonic factors were calculated by the lower-level electron structure method by using the generic scale factor. For this reaction path, five distinguishable conformers are included for reactants (see Figure S5), and six conformers are included for transition states (see Figure S12).

[b] $k_{\mathrm{TST}}^{\mathrm{RSSF}}$ comes from calculations using the higher-level electronic structure method (W3X-L//Level-2) with the reaction-specific scale factor.

[c] The rate constant ($k_{\mathrm{HPL}}^{\mathrm{DL}}$) in the high-pressure limit condition as obtained by the dual-level strategy ($k_{\mathrm{HPL}}^{\mathrm{DL}} = F_{\mathrm{act}}^{\mathrm{MS}} k_{\mathrm{TST}}^{\mathrm{RSSF}} \Gamma_{\mathrm{CVT}}^{\mathrm{LL}} \kappa_{\mathrm{SCT}}^{\mathrm{LL}}$)

[d] The rate constants ($k_{\mathrm{TST}}^{\mathrm{GSF}}$) calculated by conventional transition state theory based on the higher-level electron structure method (W3X-L//Level-2) with the generic scale factor

[e] $f = k_{\mathrm{TST}}^{\mathrm{RSSF}} / k_{\mathrm{TST}}^{\mathrm{GSF}}$, which represents the effects of scale factors using different methods on the rate constant

Table S18. The tunneling and recrossing transmission coefficients, the torsional anharmonic factors, and HPL rate constants (cm^3 $molecule^{-1}$ s^{-1}) for **1** + $(H_2O)_2$ passage through **TS-3d**[a]

T/K	F_{act}^{MS}	k_{TST}^{RSSF} [b]	Γ_{CVT}^{LL}	κ_{SCT}^{LL}	k_{HPL}^{DL} [c]	k_{TST}^{GSF} [d]	f [e]
190	5.21E-01	1.51E-06	2.59E-1	3.29E+4	6.69E-03	9.00E-08	16.78
200	5.23E-01	3.86E-07	2.81E-1	8.06E+3	4.58E-04	2.65E-08	14.57
210	5.25E-01	1.12E-07	3.04E-1	2.29E+3	4.08E-05	8.68E-09	12.91
220	5.30E-01	3.63E-08	3.26E-1	7.37E+2	4.62E-06	3.13E-09	11.62
230	5.35E-01	1.29E-08	3.47E-1	2.66E+2	6.38E-07	1.23E-09	10.47
240	5.41E-01	4.99E-09	3.68E-1	1.07E+2	1.06E-07	5.23E-10	9.55
250	5.48E-01	2.08E-09	3.88E-1	4.75E+1	2.10E-08	2.36E-10	8.80
260	5.57E-01	9.20E-10	4.08E-1	2.33E+1	4.86E-09	1.13E-10	8.12
270	5.65E-01	4.34E-10	4.28E-1	1.26E+1	1.32E-09	5.73E-11	7.58
280	5.74E-01	2.15E-10	4.47E-1	7.54	4.16E-10	3.03E-11	7.11
290	5.84E-01	1.12E-10	4.65E-1	4.98	1.51E-10	1.68E-11	6.68
298	5.92E-01	6.85E-11	4.79E-1	3.81	7.42E-11	1.07E-11	6.39
300	5.94E-01	6.05E-11	4.83E-1	3.59	6.26E-11	9.63E-12	6.29
310	6.04E-01	3.42E-11	5.00E-1	2.80	2.89E-11	5.73E-12	5.97
320	6.15E-01	2.00E-11	5.17E-1	2.32	1.48E-11	3.53E-12	5.67
330	6.27E-01	1.20E-11	5.33E-1	2.02	8.12E-12	2.22E-12	5.40
340	6.38E-01	7.45E-12	5.49E-1	1.82	4.74E-12	1.44E-12	5.17
350	6.49E-01	4.76E-12	5.64E-1	1.68	2.92E-12	9.55E-13	4.98

[a] The recrossing transmission coefficients, the tunneling transmission coefficients, and the torsional anharmonic factors were calculated by the lower-level electron structure method by using the generic scale factor. For this reaction path, five distinguishable conformers are included for reactants (see Figure S5), and seven conformers are included for transition states (see Figure S13).

[b] k_{TST}^{RSSF} comes from calculations using the higher-level electronic structure method (W3X-L//Level-2) with the reaction-specific scale factor.

[c] The rate constant (k_{HPL}^{DL}) in the high-pressure limit condition as obtained by the dual-level strategy ($k_{HPL}^{DL} = F_{act}^{MS} k_{TST}^{RSSF} \Gamma_{CVT}^{LL} \kappa_{SCT}^{LL}$)

[d] The rate constants (k_{TST}^{GSF}) calculated by conventional transition state theory based on the higher-level electron structure method (W3X-L//Level-2) with the generic scale factor

[e] $f = k_{TST}^{RSSF}/k_{TST}^{GSF}$, which represents the effects of scale factors using different methods on the rate constant

Table S19. The high-pressure limiting rate constants, $k_{3.1}$, from calculations on the loose ($k''_{3.1}$ for **C-1** + H_2O → **C-2**/**C-3**) and tight ($k'_{3.1}$ for C-**1** + H_2O → **5** + H_2O) transition states of reaction R3.1[a] (all rate constants in cm^3 molecule^{-1} s^{-1})

T/K	$k'_{3.1}$ [b]	$k''_{3.1}$ [c]	$k_{3.1}$ [d]
190	2.08E-06	9.20E-10	9.20E-10
200	3.16E-07	9.19E-10	9.16E-10
210	5.92E-08	9.16E-10	9.02E-10
220	1.31E-08	9.13E-10	8.54E-10
230	3.43E-09	9.12E-10	7.20E-10
240	1.05E-09	9.11E-10	4.87E-10
250	3.66E-10	9.11E-10	2.61E-10
260	1.47E-10	9.11E-10	1.27E-10
270	6.67E-11	9.12E-10	6.22E-11
280	3.39E-11	9.13E-10	3.27E-11
290	1.89E-11	9.15E-10	1.85E-11
298	1.26E-11	9.16E-10	1.24E-11
300	1.15E-11	9.17E-10	1.13E-11
320	7.42E-12	9.21E-10	4.99E-12
350	5.02E-12	9.29E-10	1.95E-12

[a] For reaction R3.1, the rate-determining step at low temperatures is from the reactant to the pre-reaction complex at 190 K – 230 K.

[b] $k'_{3.1}$ is the rate constants that goes through the tight transition state forming **P2**, and it comes from the sum of the four $k^{\mathrm{DL}}_{\mathrm{HPL}}$ in Tables S11-S14.

[c] $k''_{3.1}$ is the rate constant for the reaction by which **C-1** + H_2O goes through the loose transition state to produce **C-2** and **C-3**, which is calculated by variable-reaction-coordinate variational transition-state theory.

[d] The overall rate $k_{3.1}$ (cm^3 molecule^{-1} s^{-1}) for **C-1** + H_2O comes from canonical unified statistical theory with the limit of deep intermediate, and its value is calculated by the following equation:

$$k_{3.1} = \frac{k'_{3.1}\, k''_{3.1}}{k'_{3.1} + k''_{3.1}}$$

Table S20. The high-pressure limiting rate constants, $k_{4.1}$, from calculations on the loose ($k''_{4.1}$ for **1**+$(H_2O)_2$ → **C-2**/**C-3**) and tight ($k'_{4.1}$ for **1** + $(H_2O)_2$ → **5** + H_2O) transition states of reaction R4.1[a] (all rate constants in cm^3 $molecule^{-1}$ s^{-1})

T/K	$k'_{4.1}$ [b]	$k''_{4.1}$ [c]	$k_{4.1}$ [d]
190	3.62E-02	9.26E-10	9.26E-10
200	2.51E-03	9.19E-10	9.19E-10
210	2.31E-04	9.13E-10	9.13E-10
220	2.71E-05	9.07E-10	9.07E-10
230	3.96E-06	9.02E-10	9.02E-10
240	7.06E-07	8.98E-10	8.97E-10
250	1.52E-07	8.94E-10	8.89E-10
260	3.90E-08	8.91E-10	8.71E-10
270	1.17E-08	8.87E-10	8.25E-10
280	4.08E-09	8.84E-10	7.27E-10
290	1.61E-09	8.81E-10	5.69E-10
298	8.22E-10	8.79E-10	4.25E-10
300	7.04E-10	8.78E-10	3.91E-10
320	1.73E-10	8.76E-10	1.44E-10
350	3.24E-11	8.71E-10	3.12E-11

[a] For reaction R4.1, the rate-determining step at low temperatures is from the reactant to the pre-reaction complex, so the step of converting reactants to the pre-reaction complex cannot be ignored. But for other reactions, that step can be ignored.

[b] $k'_{4.1}$ is the rate constants that goes through the tight transition state forming **P2**, and it comes from the sum of the four k^{DL}_{HPL} in Tables S15-S18.

[c] $k''_{4.1}$ is the rate constant for the reaction by which **1** + $(H_2O)_2$ goes through the loose transition state to produce **C-2** and **C-3**. Its value is approximated by setting it equal to the value calculated for this kind of step in a previous investigation [3] of the CH_2OO + $(H_2O)_2$ reaction.

[d] The overall rate $k_{4.1}$ (cm^3 $molecule^{-1}$ s^{-1}) for **1** + $(H_2O)_2$ comes from canonical unified statistical theory with the limit of deep intermediate, and its value is calculated by the following equation:

$$k_{4.1} = \frac{k'_{4.1}k''_{4.1}}{k'_{4.1} + k''_{4.1}}$$

Table S21. The fitting parameters of rate constants for reactions R1a, R2.1, R3.1, and R4.1[a]

	k_1	k_2	$k_{3.1}$	$k_{4.1}$
A	171.98515	8.62E-16	6.80E-03	2.40E+100
T_0	-95.75475	19.10168	17.76892	182.76278
E	-6.29914	-1.94636	10.03072	75.82064
n	19.38626	1.86008	-36.33273	-217.63967

[a] The rate constants used for these fits were obtained by the dual-level strategy, and the results of higher-level and lower-level calculations can be found Tables S8-S20.

Table S22. Arrhenius activation energies (kcal/mol) for reactions R1a (E_{a1}), R2.1 (E_{a2}), R3.1 (E_{a3}), and R4.1 (E_{a3})[a]

T/K	E_{a1}	E_{a2}	E_{a3}	E_{a4}
220	15.27	-1.49	-3.26	0.35
230	15.21	-1.44	-4.03	0.35
250	15.19	-1.35	-5.56	-0.39
270	15.30	-1.26	-7.08	-2.04
290	15.50	-1.17	-8.59	-4.56
298	15.60	-1.14	-9.19	-5.80
300	15.63	-1.13	-9.34	-6.13
320	15.94	-1.04	-10.84	-9.80
350	16.50	-0.91	-13.08	-16.51

[a] These results are based on the rate constants in Table S19 and Table S20.

Table S23. The high-pressure-limit tunneling transmission coefficients for passage through **TS-2c**[a]

T/K	κ_{SCT}^{LL}
190	3.84
200	3.28
210	2.88
220	2.59
230	2.36
240	2.18
250	2.04
260	1.92
270	1.82
280	1.74
290	1.67
298	1.62
300	1.61
310	1.56
320	1.52
330	1.48
340	1.44
350	1.41

[a] The results are based on the lower-level electronic structure (M06CR/MG3S) by using the generic scale factor.

Table S24. The temperature-pressure dependent rate constant $k(T,P)$ of reaction R1a as calculated by RRKM/ME with the higher level of electronic structure[a] (W3X-L//Level-1)

pressure(bar)	$T = 350$	$T = 320$	$T = 298$	$T = 280$
0.0316	5.16E+02	8.72E+01	1.79E+01	3.93E+00
0.1	7.95E+02	1.24E+02	2.40E+01	5.02E+00
0.178	9.43E+02	1.42E+02	2.66E+01	5.46E+00
0.316	1.09E+03	1.57E+02	2.89E+01	5.80E+00
0.562	1.21E+03	1.71E+02	3.06E+01	6.06E+00
1	1.32E+03	1.81E+02	3.19E+01	6.24E+00
1.78	1.41E+03	1.88E+02	3.28E+01	6.37E+00
3.16	1.48E+03	1.94E+02	3.34E+01	6.44E+00
5.62	1.52E+03	1.97E+02	3.38E+01	6.49E+00
10	1.55E+03	1.99E+02	3.40E+01	6.52E+00
31.6	1.58E+03	2.01E+02	3.42E+01	6.54E+00
100	1.60E+03	2.02E+02	3.43E+01	6.55E+00
1000	1.60E+03	2.02E+02	3.43E+01	6.56E+00
pressure(bar)	$T = 260$	$T = 240$	$T = 220$	$T = 200$
0.0316	5.50E-01	5.37E-02	3.33E-03	1.16E-04
0.1	6.67E-01	6.19E-02	3.68E-03	1.24E-04
0.178	7.10E-01	6.47E-02	3.78E-03	1.26E-04
0.316	7.41E-01	6.66E-02	3.85E-03	1.27E-04
0.562	7.64E-01	6.79E-02	3.89E-03	1.28E-04
1	7.79E-01	6.87E-02	3.92E-03	1.28E-04
1.78	7.88E-01	6.92E-02	3.94E-03	1.28E-04
3.16	7.94E-01	6.95E-02	3.95E-03	1.29E-04
5.62	7.98E-01	6.97E-02	3.95E-03	1.29E-04
10	8.00E-01	6.98E-02	3.95E-03	1.29E-04
31.6	8.02E-01	6.98E-02	3.96E-03	1.29E-04
100	8.02E-01	6.99E-02	3.96E-03	1.29E-04
1000	8.03E-01	6.99E-02	3.96E-03	1.29E-04

[a] Using the reaction-specific scale factors

Table S25. The temperature-pressure dependent rate constant *k*(*T,P*) of reaction R1a as calculated by dual-level DL-MS-CVT/SCT /SS-QRRK

pressure(bar)	*T* = 350	*T* = 320	*T* = 298	*T* = 280
0.0316	4.00E+2	9.27E+1	2.26E+1	5.38
0.1	9.18E+2	1.78E+2	3.75E+1	8.00
0.178	1.26E+3	2.21E+2	4.38E+1	8.96
0.316	1.60E+3	2.59E+2	4.88E+1	9.65
0.562	1.93E+3	2.91E+2	5.25E+1	1.01E+1
1	2.21E+3	3.14E+2	5.50E+1	1.04E+1
1.78	2.44E+3	3.32E+2	5.67E+1	1.06E+1
3.16	2.62E+3	3.43E+2	5.78E+1	1.08E+1
5.62	2.75E+3	3.50E+2	5.84E+1	1.08E+1
10	2.83E+3	3.55E+2	5.88E+1	1.09E+1
31.6	2.92E+3	3.59E+2	5.91E+1	1.09E+1
100	2.95E+3	3.61E+2	5.93E+1	1.09E+1
1000	2.97E+3	3.61E+2	5.93E+1	1.09E+1
pressure(bar)	*T* = 260	*T* = 240	*T* = 220	*T* = 200
0.0316	7.77E-1	7.32E-2	4.08E-3	1.13E-4
0.1	1.05	9.28E-2	5.09E-3	1.51E-4
0.178	1.14	9.85E-2	5.37E-3	1.62E-4
0.316	1.20	1.02E-1	5.55E-3	1.69E-4
0.562	1.23	1.04E-1	5.66E-3	1.73E-4
1	1.26	1.06E-1	5.72E-3	1.76E-4
1.78	1.27	1.07E-1	5.75E-3	1.77E-4
3.16	1.28	1.07E-1	5.77E-3	1.78E-4
5.62	1.28	1.07E-1	5.79E-3	1.78E-4
10	1.29	1.07E-1	5.79E-3	1.79E-4
31.6	1.29	1.08E-1	5.80E-3	1.79E-4
100	1.29	1.08E-1	5.80E-3	1.79E-4
1000	1.29	1.08E-1	5.80E-3	1.79E-4

Table S26. The transition pressure of reaction R1a as calculated by RRKM/ME with W3X-L//Level-1[a]

T (K)	transition pressure $p_{1/2}$ (bar)
200	0.0013
220	0.0054
240	0.011
260	0.0195
280	0.035
298	0.056
320	0.10
350	0.11

[a] The transition pressure is the pressure at which the rate constant is equal to half of the high-pressure limit.

Table S27. Cartesian coordinates (Å)
(a) optimized by Level-1

Species	Cartesian coordinates (Å)		
E-CH_2(OH)CHOO	C	0.6110047795	-0.5479382741	0.1292433022
	H	0.8968701267	-1.5626320138	0.3871671863
	C	-0.7927058818	-0.0416425781	0.0687990860
	O	-1.4448612707	-0.2058743209	1.3185695344
	O	1.5444804994	0.2905045507	-0.0844870088
	O	2.8200357462	-0.1443131689	-0.0065272381
	H	-1.0376755662	0.3896726516	1.9535236502
	H	-0.7830877481	0.9978445825	-0.2675201875
	H	-1.3649431346	-0.6346067790	-0.6450373546
**TS-1a**	C	-0.2724998635	0.6124635499	-0.2591497521
	H	-0.8387410316	1.5305998167	-0.3784196497
	C	1.1711725002	0.6150095822	0.1083731150
	O	1.7779569473	-0.6439605951	0.0230584760
	O	-0.8721781456	-0.5019922781	-0.4351771478
	O	-2.0309177636	-0.0905256059	0.3936389918
	H	1.1720793779	-1.3008035486	0.3820563742
	H	1.7074338022	1.2978898211	-0.5549183975
	H	1.2253951768	1.0486182578	1.1199619902
**TS-1b**	C	-0.2486558373	0.6138626332	-0.0167535297
	H	-1.5131224650	1.0580809493	-0.0133143188
	C	1.2437074430	0.6261760929	0.0288068003
	O	1.8798983656	-0.6189785371	-0.1130368186
	O	-0.8184115324	-0.5115067458	-0.0142648076
	O	-2.2338298219	-0.0728685654	-0.0084586139
	H	1.5032959809	-1.2396926170	0.5168594243
	H	1.5907546945	1.2682572093	-0.7831420161
	H	1.5070241727	1.1381125806	0.9658378801
**TS-1c**	C	-0.1072380701	-0.6601948439	0.4791462529
	H	0.0678737440	-0.4619822661	1.5318741212
	C	-1.3888185669	-0.3456107848	-0.1695339479
	O	-0.9103544110	1.0009247030	-0.0182397867
	O	0.9013714435	-0.7270074193	-0.3267783511
	O	1.5318882492	0.5723559206	0.0199088956
	H	0.1820946715	1.0875680006	-0.1513737263
	H	-1.4678736569	-0.6798166505	-1.2005471129
	H	-2.2946184032	-0.4871876597	0.4117516553
**2**	C	0.4870390405	0.6481307709	-0.0331758207
	H	0.9520150809	1.6282342100	-0.1334264198
	C	-1.0182343365	0.6271214989	-0.0305110676
	O	-1.5445795604	-0.6684756042	0.0831748710
	O	1.1620024782	-0.2492857942	0.7815940740
	O	1.1161952473	-0.3815115688	-0.7300604625
	H	-0.9241414776	-1.2696842421	-0.3428568558
	H	-1.3774328828	1.2191258658	0.8141417778
	H	-1.3487585895	1.1246408636	-0.9522380966
	H	1.50467500	0.79834400	-0.25016100

3	C	-0.2486558373	0.6138626332	-0.0167535297
	H	-1.5131224650	1.0580809493	-0.0133143188
	C	1.2437074430	0.6261760929	0.0288068003
	O	1.8798983656	-0.6189785371	-0.1130368186
	O	-0.8184115324	-0.5115067458	-0.0142648076
	O	-2.2338298219	-0.0728685654	-0.0084586139
	H	1.5032959809	-1.2396926170	0.5168594243
	H	1.5907546945	1.2682572093	-0.7831420161
	H	1.5070241727	1.1381125806	0.9658378801
4	C	-0.2881747115	-0.1366803013	0.4730872955
	H	-0.1163324756	-0.0103073329	1.5353024734
	C	-1.5976412207	-0.3871887758	-0.1131573574
	O	-0.9804776294	0.9186736176	-0.1610644951
	O	0.7821540624	-0.6630266231	-0.2196146550
	O	1.9058420035	0.2153829861	0.0644241499
	H	1.8067982444	0.8576973464	-0.6517002948
	H	-1.6412494182	-0.9000274104	-1.0659648775
	H	-2.4668728549	-0.4633085064	0.5280787608
**TS-2a**	C	0.0439888938	-0.1964943417	0.6623313839
	H	0.5742658817	0.0537644124	1.5752444991
	C	-1.4524769557	-0.1924662947	0.5976753588
	O	-1.9342056863	-0.1408310531	-0.7234948010
	O	0.6469328487	-0.8919829643	-0.2148467016
	O	2.0456181608	-0.4823076404	-0.1946793259
	H	-1.5470863975	-0.8708601875	-1.2159858187
	H	-1.8102302497	-1.0770060411	1.1409085932
	H	-1.8161156109	0.6929111249	1.1159249308
	O	0.6258814770	1.5824720197	-0.0091099709
	H	0.1672250580	1.6917981586	-0.8503183040
	H	1.4508305801	1.0070348071	-0.2143208437
**TS-2b**	C	0.0313409389	-0.2822098048	0.6591386034
	H	0.5529410215	-0.0574006875	1.5848452225
	C	-1.4633658702	-0.2114251543	0.5746659982
	O	-1.9236343332	-0.0537886941	-0.7383947521
	O	0.6579651758	-0.9561908405	-0.2209497463
	O	2.0382269918	-0.4862799417	-0.1604468379
	H	-1.4241954625	0.6733253875	-1.1272204239
	H	-1.8746957595	-1.1462285867	0.9647329320
	H	-1.7845064124	0.6004035156	1.2360738913
	O	0.5978947785	1.4563581397	-0.1230069881
	H	0.8323513302	2.1301032106	0.5221162108
	H	1.4674466014	0.8157134561	-0.2353271100
**C-1**	C	-0.1324326927	-0.7713533218	0.4075971624
	H	0.0980747474	-0.5048399079	1.4350244750
	C	-1.5131991328	-0.7414439425	-0.1591722553
	O	-2.0064740096	0.5680273481	0.0519283856
	O	0.8308236789	-0.8859982204	-0.3939161722
	O	2.0800314814	-0.5542673242	0.1084299038
	H	-1.2973441343	1.1912502377	-0.1698264015
	H	-1.4786396296	-1.0194817892	-1.2155213524
	H	-2.1703025732	-1.4263139118	0.3763196831
	O	0.6420988270	1.8339290618	-0.0962505596

	H	0.9662952678	2.7033969527	0.1414557527
	H	1.4114771696	1.2378238175	-0.0273826218
**5**	C	0.0923374237	0.2440913701	0.5594333977
	H	0.5250706345	0.4441948136	1.5404787064
	C	-1.3350557906	-0.2780173141	0.6322975244
	O	-1.9156594665	-0.2895910472	-0.6656136099
	O	0.8180756144	-0.7923005751	-0.0743543639
	O	2.1988505540	-0.3709868542	-0.1163469773
	H	-1.4904026833	-0.9915699660	-1.1667516405
	H	-1.3472750785	-1.2682139758	1.0920303749
	H	-1.9311273439	0.4059951316	1.2336801570
	O	0.1653810170	1.4281189487	-0.1801375517
	H	-0.5005948578	1.3469655151	-0.8763715622
	H	2.1346539771	0.3965849533	-0.7045074548
H_2O	O	0.0000000000	0.0000000000	0.1181142055
	H	0.0000000000	0.7573463466	-0.4697881027
	H	0.0000000000	-0.7573463466	-0.4697881027

(b) optimized by Level-2

Species	Cartesian coordinates (Å)			
**1**	C	0.6094993022	-0.5517866809	0.1279734809
	H	0.9011728330	-1.5781434802	0.3770395378
	C	-0.7967619750	-0.0417468011	0.0712815001
	O	-1.4462524546	-0.2038761068	1.3214954257
	O	1.5459933795	0.2929217786	-0.0751584700
	O	2.8228379633	-0.1371016231	-0.0010674142
	H	-1.0289107172	0.3972863176	1.9518435830
	H	-0.7852138971	1.0053256592	-0.2725692282
	H	-1.3732468835	-0.6418644132	-0.6471074451
**TS-1a**	C	-0.26399800	0.60688400	-0.23523500
	H	-0.81579200	1.53964400	-0.33373900
	C	1.16893800	0.60372700	0.10519100
	O	1.78799700	-0.63808000	0.07959100
	O	-0.87059100	-0.48546100	-0.43339700
	O	-2.00269300	-0.10885800	0.39047800
	H	1.15384300	-1.32494100	0.30406600
	H	1.68865400	1.28574400	-0.57848300
	H	1.22594200	1.09507700	1.09505000
**TS-2a**	C	0.0401833053	-0.1904491514	0.6525981613
	H	0.5728579540	0.0407208865	1.5814997410
	C	-1.4594346733	-0.1921923276	0.5915334451
	O	-1.9454489806	-0.1560213614	-0.7273033586
	O	0.6428870862	-0.8818582659	-0.2348617432
	O	2.0493572709	-0.4742454602	-0.2070239050
	H	-1.5719794207	-0.9111718897	-1.2016683753
	H	-1.8104431502	-1.0789870313	1.1541767353
	H	-1.8270813132	0.7038093836	1.1082473633
	O	0.6307412322	1.5840571487	0.0064622890
	H	0.2067641666	1.7158943230	-0.8554254284

	H	1.4662245228	1.0164757457	-0.1989059246
**TS-2b**	C	0.0288254821	-0.2865191737	0.6672244229
	H	0.5502581178	-0.0664089977	1.6063106128
	C	-1.4685501982	-0.2165285709	0.5727248018
	O	-1.9166221129	-0.0408488295	-0.7412816933
	O	0.6617557871	-0.9602991381	-0.2143075588
	O	2.0436792103	-0.4779755976	-0.1516077673
	H	-1.4092465820	0.6991076280	-1.1075811624
	H	-1.8817142187	-1.1660775385	0.9498973187
	H	-1.7974443827	0.5901390327	1.2521491569
	O	0.5855193683	1.4458614505	-0.1282572953
	H	0.8466231417	2.1489168078	0.4823592456
	H	1.4646853875	0.8130129269	-0.2514030817
**TS-2c**	C	-0.6119633978	-0.8271004625	0.3097073509
	O	0.3642191342	-1.0747860210	-0.4721183345
	O	1.6410159700	-0.9189713596	0.2317335696
	H	-0.4703464722	-0.9354501690	1.3889992523
	O	1.1677591512	1.4053229896	-0.2217426371
	H	1.5761199179	0.3796084768	0.0252675371
	H	1.5373007648	2.0521464819	0.3908334212
	C	-1.7648527611	-0.1047576181	-0.2665222710
	H	-1.8010186561	-0.1385596954	-1.3641752530
	H	-2.7381003914	-0.3134114520	0.1928430840
	O	-1.1667923244	1.0484800859	0.2890994538
	H	-0.0467499349	1.2912397434	-0.0430351734
**TS-2d**	C	-0.5980329127	-0.8387995487	0.2964979615
	O	0.3843986303	-1.0609564305	-0.4853740107
	O	1.6561295157	-0.8972598124	0.2271725747
	H	-0.4539769009	-0.9495191091	1.3754662323
	O	1.1519645109	1.4503280612	-0.0175508399
	H	1.5622807120	0.4027408012	0.1143421041
	H	1.4807819060	1.8166539156	-0.8469398232
	C	-1.7621995624	-0.1326780295	-0.2762401634
	H	-1.7963520274	-0.1637467113	-1.3746353546
	H	-2.7324142728	-0.3679002475	0.1769454796
	O	-1.1988458159	1.0319536915	0.2886669096
	H	-0.0701897827	1.3021014194	0.0180389300
**TS-3a**	C	-0.4504858964	-0.5050211620	-0.2763596022
	H	-0.3963509389	-0.5556676073	-1.3699466652
	O	0.4805110027	-1.1029512922	0.3743377561
	O	1.6822218045	-1.2246847527	-0.4646789596
	O	2.3495582869	1.0352780978	0.2393154812
	H	2.7553325948	1.0495296581	1.1134619505
	H	2.2454580187	0.0158648539	-0.0477209274
	O	-0.0539383047	1.3026867540	-0.1742621171
	H	0.9827890011	1.2960169950	0.0984892989
	H	-0.0652866777	1.7018417173	-1.0578226191
	C	-1.7956968534	-0.4973468195	0.4095786980
	H	-2.2454896606	-1.4871269767	0.2381643541
	H	-1.6405575021	-0.3675314706	1.4929533295

	O	-2.6672858045	0.4575138451	-0.1437944521
	H	-2.3212620703	1.3226671601	0.1123244744
**TS-3b**	C	-0.3758663411	-0.4312595013	-0.2050726644
	H	-0.1076708386	-0.1852431542	-1.2399709862
	O	0.4270995932	-1.2003957910	0.4395767554
	O	1.7395746252	-1.2730867862	-0.2160834731
	O	2.2345271651	1.1262741438	-0.3080088808
	H	3.0043709625	1.3929811642	0.2063362078
	H	2.1451169123	0.0486700652	-0.2594161507
	O	-0.0851083061	1.3078442442	0.3921960032
	H	0.9567847738	1.4015133794	0.1251344540
	H	-0.0752239022	1.2282879221	1.3596267953
	C	-1.8362208141	-0.5469571840	0.1535968129
	H	-2.1869792538	-1.5101380680	-0.2469998987
	H	-1.9429614826	-0.5775118458	1.2527519174
	O	-2.6010038960	0.4628175554	-0.4526441099
	H	-2.1829331978	1.3058648559	-0.2276187821
**TS-3c**	C	-0.4016889147	-0.4408787056	-0.2149554863
	H	-0.1733173054	-0.2470317907	-1.2698028158
	O	0.4201471877	-1.1870826007	0.4297077529
	O	1.7059290961	-1.2836417278	-0.2746367393
	O	-0.0744326316	1.3274141646	0.2740576335
	H	-0.1143179684	1.3476559305	1.2441984826
	H	0.9827238543	1.3818323904	0.0773812139
	O	2.3293547169	1.0834421837	-0.0205270990
	H	2.1968023079	0.0213771334	-0.1259123629
	H	2.8488336691	1.3897587497	-0.7722964849
	C	-1.8476198537	-0.5241333779	0.2055798408
	H	-2.2177262624	-1.5090987830	-0.1165311380
	H	-1.9137964818	-0.4811250181	1.3074387741
	O	-2.6312863493	0.4481938498	-0.4382294652
	H	-2.2048910645	1.3027516017	-0.2851521064
**TS-3d**	C	0.4476268985	-0.5018303140	0.2763078881
	H	0.4043950611	-0.5692540343	1.3698111907
	O	-0.4813225305	-1.1074640585	-0.3737695805
	O	-1.6861729308	-1.2136733119	0.4617202273
	O	0.0444705079	1.2861838536	0.2004034675
	H	0.0628553966	1.6522865451	1.0974489912
	H	-0.9930665930	1.2956446034	-0.0861431824
	O	-2.3013720599	0.9827176379	-0.4446238847
	H	-2.2372830548	-0.0118422691	-0.0597873282
	H	-3.0604824501	1.4095875029	-0.0327769857
	C	1.7904867062	-0.4885142265	-0.4149638801
	H	2.2375688891	-1.4825244544	-0.2632063987
	H	1.6322015951	-0.3370055473	-1.4947751331
	O	2.6668403607	0.4544544997	0.1539899934
	H	2.3401492037	1.3247415733	-0.1094963847
**C-1**	C	-0.1339288497	-0.7825542066	0.4187938638
	H	0.0976905656	-0.5436392347	1.4638380441
	C	-1.5140204766	-0.7336947412	-0.1591993606
	O	-2.0165850684	0.5650043437	0.0865088089
	O	0.8355326378	-0.8879305401	-0.3829338393

	O	2.0899295777	-0.5899964011	0.1296779475
	H	-1.3212883173	1.1955310287	-0.1722079872
	H	-1.4680892847	-0.9867614459	-1.2310118119
	H	-2.1769927462	-1.4427907226	0.3558843900
	O	0.6443250981	1.8437455445	-0.1177416612
	H	0.9590972721	2.7218328113	0.1209472708
	H	1.4347385914	1.2719825639	-0.0538696650
**C-2**	C	0.0740901017	-0.8610917366	0.5083818428
	H	-0.1544156052	-0.0977214235	1.2611593822
	C	1.4684629470	-1.2545638932	0.1066803994
	O	1.9842517265	-0.3165244906	-0.8108707215
	O	-0.8959374568	-1.3655871600	-0.1190486410
	O	-2.1588361153	-0.8716049724	0.1952800359
	H	1.8998195152	0.5684059929	-0.4065302162
	H	1.4385555079	-2.2307705806	-0.3944457278
	H	2.0824989212	-1.3324918125	1.0212008092
	O	1.0467090056	1.9444494125	0.5008998379
	H	1.3297812048	2.8348397879	0.7343425257
	H	-1.9237490694	0.8171599215	-0.1694409970
	H	0.1353823586	2.0419880767	0.1493036970
	O	-1.5234082018	1.6912980040	-0.3980352074
	H	-1.8434278400	1.8716048738	-1.2888840193
**C-3**	C	0.3089107844	-1.0042589178	0.3264928776
	H	0.0196225985	-0.9028590642	1.3809909543
	O	-0.6031595608	-1.2161791966	-0.5111295242
	O	-1.9051866694	-1.3201080143	0.0020666198
	O	0.5117657093	2.0013527597	-0.4239712715
	H	0.5022493840	2.6399742492	-1.1442694810
	H	-0.4277633370	1.9012177555	-0.1584685773
	O	-2.0418073782	1.3590331630	0.2646103375
	H	-2.1978169678	0.3895311921	0.1561533226
	H	-2.5840494742	1.6174066613	1.0171431547
	C	1.7256504334	-0.8639443816	-0.1476913528
	H	2.2649553018	-1.7879641937	0.1181913600
	H	1.7259763204	-0.7616764413	-1.2456617913
	O	2.3476125192	0.1984231598	0.5233230448
	H	1.8753093362	1.0066832688	0.2425163268
**C-4**	C	0.4997758260	0.2342774296	-0.6285875435
	H	0.2751751303	0.7162975260	-1.5898834836
	O	-0.0464494156	1.0073831458	0.4171295236
	O	-1.3335268463	1.5085653289	-0.0152300252
	O	-0.0493454605	-1.0665403299	-0.6663663218
	H	0.4510321057	-1.5669172835	0.0007368151
	H	-1.9616266342	-1.2289721462	-0.1926947589
	O	-2.7475242742	-0.8425163045	0.2292397836
	H	-1.9318791096	0.7860622345	0.2760916588
	H	-2.9826851971	-1.4572193528	0.9323918654
	C	1.9935732803	0.1613726605	-0.3343872897
	H	2.4972535151	-0.3523773911	-1.1642103018
	H	2.4046725800	1.1774134489	-0.2308610526
	O	2.2207337436	-0.6273128227	0.8283435126
	H	1.9288577565	-0.1040941436	1.5871606180

**5**	C	0.0866552475	0.2523274274	0.5565632422
	H	0.5267472515	0.4335356687	1.5481366314
	C	-1.3399907753	-0.2781263976	0.6266362780
	O	-1.9178309520	-0.3005816225	-0.6716995343
	O	0.8157340891	-0.7719589352	-0.1010105336
	O	2.2044489863	-0.3718620019	-0.1010552054
	H	-1.5003228740	-1.0250344506	-1.1564959868
	H	-1.3487573264	-1.2722849650	1.1010791486
	H	-1.9445857549	0.4137000357	1.2281692208
	O	0.1574087629	1.4510964815	-0.1596820145
	H	-0.5035458809	1.3727786538	-0.8674788221
	H	2.1782932264	0.3716811057	-0.7293254242
H_2O	O	0.0000000000	0.0000000000	0.1237506983
	H	0.0000000000	0.7561720962	-0.4726063491
	H	0.0000000000	-0.7561720962	-0.4726063491
**4**	C	0.4038486369	0.6545423755	-0.1627782859
	H	0.7276472269	1.6859990021	-0.3354074201
	C	1.2055836390	-0.5062290110	-0.5456637974
	O	1.0627574239	-0.0667480970	0.8428743927
	O	-0.9884548056	0.6887129658	-0.1370125040
	O	-1.4935312277	-0.6611447650	-0.0182584900
	H	-1.5555783750	-0.7400061414	0.9484608705
	H	0.6977409391	-1.4440694312	-0.7733745276
	H	2.2033655425	-0.3584698977	-0.9669712383

(c) optimized by M06CR/MG3S

Species	Cartesian coordinates (Å)			
**1**	C	-0.14269800	-0.05937100	0.40873800
	H	-0.29373500	-0.78612700	1.20462400
	C	1.17349600	0.54499700	0.08099800
	O	2.15991200	-0.44560300	-0.10545500
	O	-1.13583000	0.25662400	-0.28573800
	O	-2.32903900	-0.25480300	-0.03148500
	H	1.97848700	-0.93769400	-0.90791200
	H	1.06194400	1.20929300	-0.78057200
	H	1.50816800	1.15102500	0.92685800
**TS-1a**	C	-0.26399800	0.60688400	-0.23523500
	H	-0.81579200	1.53964400	-0.33373900
	C	1.16893800	0.60372700	0.10519100
	O	1.78799700	-0.63808000	0.07959100
	O	-0.87059100	-0.48546100	-0.43339700
	O	-2.00269300	-0.10885800	0.39047800
	H	1.15384300	-1.32494100	0.30406600
	H	1.68865400	1.28574400	-0.57848300
	H	1.22594200	1.09507700	1.09505000
**TS-2a**	C	0.03030500	-0.12827000	0.66841600
	H	0.55680600	0.12517100	1.58569800
	C	-1.45245000	-0.03374000	0.59154900
	O	-1.94751600	-0.09330400	-0.71428600

	O	0.59676600	-0.91603000	-0.15096800
	O	1.98628500	-0.64349500	-0.16846500
	H	-1.60275900	-0.87274600	-1.15575700
	H	-1.85973500	-0.82497700	1.23938200
	H	-1.75439300	0.91877700	1.02635300
	O	0.77740400	1.53915300	-0.09806400
	H	0.33301200	1.66331900	-0.94190600
	H	1.55642300	0.87192000	-0.25929500
**TS-2b**	C	0.02158000	-0.25992200	0.64876000
	H	0.54021100	-0.06631700	1.58630100
	C	-1.46099700	-0.16561100	0.57133800
	O	-1.94891300	-0.07265600	-0.72820700
	O	0.63864200	-0.93135200	-0.23440300
	O	1.99910500	-0.53696400	-0.20551400
	H	-1.44711000	0.60158400	-1.19572500
	H	-1.87703500	-1.06815700	1.03262900
	H	-1.76012000	0.67955400	1.20354100
	O	0.65765300	1.47490600	-0.11120500
	H	0.90397700	2.13672200	0.53746100
	H	1.50467500	0.79834400	-0.25016100
**TS-2c**	C	-0.61248000	-0.82607300	0.31023800
	O	0.37940500	-1.05497500	-0.44625000
	O	1.62777700	-0.91333900	0.22255800
	H	-0.51470000	-0.98453900	1.37885600
	O	1.18565600	1.48137400	-0.05163600
	H	1.57688700	0.45892100	0.09052300
	H	1.52055900	1.85630800	-0.86582900
	C	-1.76854100	-0.15638300	-0.27838000
	H	-1.79271100	-0.15461800	-1.36732900
	H	-2.74106000	-0.36141300	0.16113300
	O	-1.15261600	0.96113400	0.32451700
	H	-0.08463200	1.28652100	0.01798900
**TS-2d**	C	-0.62828400	-0.81704600	0.31908800
	O	0.36093900	-1.06419400	-0.43589700
	O	1.61104900	-0.93366400	0.23199500
	H	-0.54031600	-0.98597000	1.38686200
	O	1.19682900	1.43885500	-0.22979400
	H	1.58766300	0.43708600	0.01275600
	H	1.60069900	2.10497200	0.32608200
	C	-1.77191700	-0.12981800	-0.27361600
	H	-1.79044700	-0.12067100	-1.36198000
	H	-2.74907400	-0.31127700	0.16525700
	O	-1.12389900	0.96908200	0.33537400
	H	-0.06665100	1.27640600	-0.01523700
**TS-3a**	C	-0.46076800	-0.49583800	-0.27817600
	H	-0.41268600	-0.50822400	-1.36548300
	O	0.45847100	-1.12041700	0.34443600
	O	1.64804600	-1.26712500	-0.42358200
	O	2.38753500	0.99247500	0.21107600
	H	2.79427400	1.04966100	1.07472200

	H	2.23739400	-0.04611000	-0.05546000
	O	-0.02926800	1.32066400	-0.13582200
	H	1.03036200	1.28701600	0.09340900
	H	-0.10696100	1.74310300	-0.99582600
	C	-1.79945300	-0.49394100	0.39689900
	H	-2.22963400	-1.48559900	0.23672800
	H	-1.65230200	-0.36616700	1.47293500
	O	-2.67922000	0.44277700	-0.15549100
	H	-2.38363700	1.31800500	0.10168800
**TS-3b**	C	-0.38735200	-0.42547700	-0.21128400
	H	-0.11895200	-0.14219900	-1.22780000
	O	0.39784300	-1.22276300	0.39976400
	O	1.69455500	-1.32154600	-0.17200900
	O	2.26099700	1.06121400	-0.30846300
	H	3.03151500	1.34384700	0.18191900
	H	2.13197500	-0.03457400	-0.24644500
	O	-0.04674100	1.31273800	0.43974100
	H	1.00769000	1.36604600	0.15565500
	H	-0.06718200	1.22057100	1.39732800
	C	-1.83956900	-0.52313300	0.14103400
	H	-2.19677000	-1.47012000	-0.27151000
	H	-1.94554900	-0.58640700	1.23089500
	O	-2.59437700	0.50191100	-0.43136300
	H	-2.17941800	1.34205800	-0.21990100
**TS-3c**	C	-0.40612300	-0.43271400	-0.21675500
	H	-0.17120700	-0.19597100	-1.25284400
	O	0.39524800	-1.20904200	0.39766200
	O	1.67065000	-1.33170600	-0.21715200
	O	-0.03743800	1.33247600	0.33740500
	H	-0.09566200	1.32566900	1.29839600
	H	1.02981000	1.35537600	0.11322400
	O	2.35136900	1.02885600	-0.07673500
	H	2.17613000	-0.05165600	-0.15357200
	H	2.80741800	1.34856900	-0.85397500
	C	-1.84678700	-0.50554300	0.18585400
	H	-2.21953400	-1.47164100	-0.16340600
	H	-1.91950000	-0.50927900	1.28017100
	O	-2.61660600	0.49048400	-0.41845100
	H	-2.19577300	1.33992700	-0.26442200
**TS-3d**	C	0.46224800	-0.49393300	0.28454900
	H	0.42983200	-0.52153000	1.37247000
	O	-0.46015900	-1.12107300	-0.33322000
	O	-1.64810600	-1.25576300	0.43846300
	O	0.02502100	1.30949000	0.17088200
	H	0.10193700	1.70389000	1.04356200
	H	-1.03459100	1.28545400	-0.08196500
	O	-2.34223300	0.94596500	-0.39682400
	H	-2.22973200	-0.06890900	-0.02037400
	H	-3.07528400	1.38334800	0.03324300
	C	1.79381200	-0.48596600	-0.40527600
	H	2.22637600	-1.47882900	-0.26091600

	H	1.63394600	-0.34548400	-1.47769400
	O	2.67871100	0.44618000	0.14839200
	H	2.38529200	1.32306400	-0.10549800
**C-1**	C	-0.14247000	-0.78187800	0.41231900
	H	0.06877600	-0.52602700	1.44911900
	C	-1.51046200	-0.74952700	-0.16164100
	O	-2.04077300	0.53069900	0.08732700
	O	0.82879800	-0.90601100	-0.35965200
	O	2.06870900	-0.66338100	0.10444600
	H	-1.38284700	1.19600800	-0.15110600
	H	-1.46687900	-0.99930600	-1.22569100
	H	-2.15563700	-1.47282700	0.34076700
	O	0.69101600	1.89959200	-0.07670300
	H	1.00853900	2.78767400	0.08006100
	H	1.46363700	1.31571500	-0.04056000
**C-2**	C	0.07599600	-0.87703200	0.49140000
	H	-0.13111600	-0.08031200	1.20317800
	C	1.45440000	-1.28707000	0.10483800
	O	2.01568700	-0.32806800	-0.75384000
	O	-0.89908800	-1.40011400	-0.08285800
	O	-2.14086600	-0.94459400	0.18198900
	H	1.92930000	0.55619400	-0.36520900
	H	1.41388600	-2.23642300	-0.42789500
	H	2.04567000	-1.42394200	1.01885100
	O	1.07047400	2.04529200	0.49814300
	H	1.35356900	2.93537200	0.70145900
	H	-1.94401300	0.80610200	-0.17164500
	H	0.15817600	2.09393900	0.16029200
	O	-1.56217300	1.68193000	-0.39645800
	H	-1.88012500	1.89811600	-1.27225200
**C-3**	C	0.28175500	-0.96094900	0.30668300
	H	-0.02377700	-0.72025300	1.32511200
	O	-0.60101400	-1.30126000	-0.50118300
	O	-1.89007700	-1.38332400	-0.07944500
	O	0.52357900	2.05382900	-0.46250600
	H	0.51927500	2.71811800	-1.14960500
	H	-0.39950600	1.92316900	-0.17907600
	O	-2.01939900	1.29894300	0.34420900
	H	-2.17968300	0.34070100	0.19450900
	H	-2.59616000	1.58022900	1.05228700
	C	1.69795300	-0.86740900	-0.13482600
	H	2.19398900	-1.80536800	0.14220200
	H	1.72730600	-0.79517000	-1.22748000
	O	2.35536700	0.17254800	0.51898500
	H	1.93266100	1.00282800	0.25043000
**C-4**	C	0.55292300	-0.36602100	0.29349400
	H	0.82270700	-0.95586800	1.17533700
	O	-0.42460200	-1.03807600	-0.44286400
	O	-1.48648100	-1.37548600	0.43139900
	O	0.12190300	0.91842400	0.69576100
	H	-0.36124400	0.84645300	1.52122900
	H	-1.72316600	1.37559400	-0.42718400

	O	-2.64626600	1.11729200	-0.34045500
	H	-2.14813300	-0.70421100	0.19077900
	H	-3.08301400	1.35252900	-1.15860700
	C	1.73672000	-0.18203000	-0.63457800
	H	2.10193100	-1.15899100	-0.94522600
	H	1.39398200	0.35498600	-1.52731500
	O	2.78707100	0.48584200	0.01357800
	H	2.44607400	1.31385300	0.35814000
**5**	C	0.10538400	0.15714300	0.54225100
	H	0.53616600	0.22734400	1.54412400
	C	-1.29591200	-0.42228100	0.58446700
	O	-1.92172300	-0.14384500	-0.65373500
	O	0.87859100	-0.74170100	-0.22423800
	O	2.22900300	-0.34062800	-0.14358200
	H	-2.59472000	-0.79339400	-0.84696400
	H	-1.22509600	-1.49445700	0.77709000
	H	-1.83880500	0.05220100	1.40742200
	O	0.12784300	1.43806000	-0.00408600
	H	-0.48987500	1.45089600	-0.74322600
	H	2.24578900	0.45314500	-0.69362600
H_2O	O	0.00000000	0.00000000	0.11540500
	H	0.00000000	0.76305700	-0.46161900
	H	0.00000000	-0.76305700	-0.46161900
**TS-3e**	C	-0.92755100	-0.43097500	-0.47197000
	H	-0.24906300	-0.10285200	-1.25494300
	C	-2.00896000	0.44393400	-0.03950900
	O	-0.99174400	1.10560800	0.67217600
	O	-0.66427000	-1.51643400	0.11715200
	O	0.62513700	-2.01990400	-0.15711500
	H	-0.11068300	1.63483800	0.11139200
	H	-2.73954300	-0.01492000	0.62459700
	H	-2.48852100	1.02526000	-0.82853900
	O	0.98124500	1.91492100	-0.42687500
	H	1.37291500	2.75581700	-0.19655700
	H	1.39148200	-1.07674000	0.06816500
	H	1.61358300	0.98185100	-0.08904700
	O	2.09889500	-0.12760100	0.23281100
	H	2.43478700	-0.13373400	1.12861900
**TS-3f**	C	-1.00798300	0.16211800	0.46497100
	H	-0.25195600	0.05747800	1.23780000
	C	-1.83413500	-0.97788800	0.11095000
	O	-0.66059500	-1.24666800	-0.63358800
	O	-1.10103700	1.28031900	-0.12704000
	O	0.02979700	2.11100300	0.06174700
	H	0.33846800	-1.59031900	-0.13696400
	H	-2.68543900	-0.77390100	-0.53363500
	H	-2.06503800	-1.68709600	0.90439000
	O	1.50584800	-1.65370200	0.31270500
	H	2.04986100	-2.28910600	-0.15285200
	H	1.05169100	1.37686100	-0.12542400
	H	1.83240900	-0.59956100	0.07796400
	O	1.98072200	0.67171800	-0.22467900

	H	2.74483300	1.09890100	0.16003500
**TS-3g**	C	1.02528100	-0.22250600	0.45382200
	H	0.31391600	-0.07268400	1.26095600
	C	1.91202600	0.85874100	0.06583700
	O	0.73246700	1.22817100	-0.61769100
	O	1.00642600	-1.34296000	-0.13847500
	O	-0.16289000	-2.09635500	0.13027900
	H	-0.26075400	1.63024300	-0.11007500
	H	2.71567400	0.59782600	-0.61899100
	H	2.24012000	1.53696300	0.85314400
	O	-1.44728500	1.75265200	0.19160500
	H	-1.68468600	2.13375500	1.03546000
	H	-1.17577000	-1.29997400	-0.05207800
	H	-1.81392900	0.69345700	0.05795100
	O	-2.05731100	-0.57752400	-0.13956100
	H	-2.52967000	-0.74887200	-0.95357600
**C-5**	C	-1.51285100	0.38509600	0.21282300
	H	-1.99817500	0.89601200	1.03770200
	C	-2.12322100	-0.72097200	-0.49478800
	O	-1.12681100	-0.95136400	0.50460600
	O	-0.63944800	1.15113800	-0.49849600
	O	0.17281600	1.86359400	0.42508400
	H	0.60496700	-1.72495800	0.18739200
	H	-1.80032000	-0.91133700	-1.51269800
	H	-3.12452000	-1.04640800	-0.23579300
	O	1.51720000	-1.98810900	-0.00040300
	H	1.76350500	-2.62424500	0.67022000
	H	1.05432800	1.46362300	0.25631400
	H	2.35917800	-0.33510100	-0.11832500
	O	2.57665200	0.61217800	-0.13114900
	H	2.95419500	0.79817900	-0.99016100
**4**	C	0.39957000	0.64495300	-0.17306100
	H	0.72924100	1.66258800	-0.36343900
	C	1.19569100	-0.51209800	-0.52500600
	O	1.05607100	-0.05756000	0.83362300
	O	-0.98631000	0.68369000	-0.14692500
	O	-1.52547300	-0.61299800	-0.02221100
	H	-1.53000700	-0.74334200	0.93389300
	H	0.69228700	-1.44494600	-0.74341900
	H	2.18260700	-0.37649000	-0.95452700
**2**	C	0.48254700	0.64931900	-0.03462800
	H	0.94798100	1.63147700	-0.12746500
	C	-1.01602300	0.62428900	-0.03286800
	O	-1.55275800	-0.65733700	0.08677900
	O	1.16626900	-0.25970200	0.74619000
	O	1.12744800	-0.37640600	-0.70576600
	H	-0.96083300	-1.28461700	-0.33674200
	H	-1.36985600	1.23063000	0.80535200
	H	-1.34410500	1.12842600	-0.95379900
**TS-4a**	C	-0.15494700	0.66091000	0.60492800
	H	0.11668700	1.10162100	1.56884400
	C	-1.45010300	-0.15085400	0.51398300

	O	-1.18276300	-0.73898900	-0.80299400
	O	0.78609700	-0.67923300	0.50142600
	O	1.96299400	-0.32225500	-0.17851500
	H	-0.16883400	-1.07179500	-0.43781900
	H	-1.55642500	-0.95511200	1.23625300
	H	-2.33782300	0.47198900	0.45841300
	O	0.02222700	1.33262600	-0.48271900
	H	-0.80251000	0.15505400	-1.20481200
	H	1.67076600	0.50071600	-0.63193200
**TS-4b**	C	-0.21580100	0.61133900	0.54071700
	H	0.32451300	0.76687800	1.49227100
	C	-1.34217200	-0.37463800	0.65988900
	O	-1.91502100	-0.74927900	-0.55433300
	O	1.06195300	-0.21767500	-0.39812000
	O	2.28854800	-0.69210800	0.07986400
	H	-1.21954600	-1.00748900	-1.16310400
	H	-0.98180200	-1.23095700	1.24424500
	H	-2.11795000	0.11599800	1.25811800
	O	-0.20512100	1.53308300	-0.35525900
	H	0.83226100	0.88517200	-0.76771700
	H	2.66748200	0.05803300	0.55533500
**TS-4c**	C	0.11416200	0.19940800	0.12235100
	H	1.11369600	-0.05219000	0.87318600
	C	-1.10150700	-0.59064900	0.59586600
	O	-2.21183300	-0.34651300	-0.24720900
	O	0.82212800	-0.32263800	-0.83095000
	O	2.34005300	-0.51459800	0.22680700
	H	-2.09898500	-0.82789500	-1.06840700
	H	-0.84131200	-1.64952000	0.64299800
	H	-1.38619700	-0.24561100	1.58741000
	O	-0.06650800	1.57702400	0.10461300
	H	-0.88542200	1.77705900	-0.35837800
	H	2.95156400	0.19940000	-0.01219100
**6**	C	-0.82561900	0.47977400	0.00000000
	H	-1.41998900	1.41542700	0.00000000
	C	0.66166800	0.64160300	0.00000000
	O	1.33851400	-0.57120000	0.00000000
	H	0.92387200	1.25118800	-0.87739700
	H	0.92387200	1.25118800	0.87739700
	O	-1.35346500	-0.60059600	0.00000000
	H	0.67555600	-1.27170000	-0.00000100
**7**	C	-0.82561900	0.47977400	0.00000000
	H	-1.41998900	1.41542700	0.00000000
	C	0.66166800	0.64160300	0.00000000
	O	1.33851400	-0.57120000	0.00000000
	H	0.92387200	1.25118800	-0.87739700
	H	0.92387200	1.25118800	0.87739700
	O	-1.35346500	-0.60059600	0.00000000
	H	0.67555600	-1.27170000	-0.00000100
H_2O_2	O	0.00000000	-0.70888400	-0.05695900
	H	0.79016500	-0.90550600	0.45567300
	O	0.00000000	0.70888400	-0.05695900

	H	-0.79016500	0.90550600	0.45567300

(d) optimized by M11-L/MG3S

Species	Cartesian coordinates (Å)			
**1**	C	-0.14525800	-0.12424000	0.36931700
	H	-0.29524200	-0.96309100	1.06239200
	C	1.15947000	0.51536500	0.12019100
	O	2.15003200	-0.40896600	-0.12867600
	O	-1.12907400	0.29680600	-0.25701100
	O	-2.30258200	-0.24559100	-0.03970600
	H	1.95281300	-0.85376500	-0.94814000
	H	1.03787000	1.27279000	-0.67517100
	H	1.47227700	1.05932600	1.02701700
**TS-1a**	C	-0.26149700	0.60757500	-0.25589600
	H	-0.82087800	1.55215900	-0.32715100
	C	1.15470500	0.58570900	0.10835500
	O	1.73068900	-0.64168600	0.09751800
	O	-0.85433900	-0.47120300	-0.47244100
	O	-1.93772200	-0.11022400	0.42749500
	H	1.04787100	-1.29232000	0.25678800
	H	1.70303600	1.26959400	-0.56518200
	H	1.20169700	1.09576500	1.10021200
**TS-2a**	C	0.04752200	-0.13564200	0.66231000
	H	0.58633300	0.11636900	1.58658400
	C	-1.42861200	-0.06215300	0.58041900
	O	-1.88801400	-0.09591900	-0.70836600
	O	0.59954000	-0.90413800	-0.15958900
	O	1.97701300	-0.57495500	-0.16755500
	H	-1.50203800	-0.85177900	-1.14475500
	H	-1.83370600	-0.87621700	1.21797900
	H	-1.75677200	0.87875100	1.04018700
	O	0.69466100	1.50743800	-0.08599400
	H	0.23896900	1.57048800	-0.92380500
	H	1.48814500	0.88974600	-0.26052800
**TS-2b**	C	0.03613800	-0.35691500	0.62544200
	H	0.56548400	-0.19706100	1.57616200
	C	-1.43913800	-0.28010000	0.52884000
	O	-1.86728300	0.02265600	-0.72539700
	O	0.66813300	-0.90306400	-0.30572200
	O	1.98896200	-0.41197200	-0.21163400
	H	-1.34043100	0.76471200	-1.02265200
	H	-1.85744000	-1.25901300	0.82104600
	H	-1.77273400	0.43729800	1.30405200
	O	0.54093300	1.44227200	-0.02069300
	H	0.74899500	2.05804200	0.67479300
	H	1.42815700	0.81897800	-0.17152800
**TS-3a**	C	-0.44063800	-0.52683400	-0.27653100
	H	-0.39703600	-0.56820900	-1.37497600
	O	0.48049600	-1.09490200	0.35449300

	O	1.64309200	-1.21004700	-0.45553300
	O	2.29170100	1.00145000	0.25917100
	H	2.56672000	0.99852100	1.16838400
	H	2.17786900	-0.01516900	-0.04074000
	O	-0.05999000	1.28677600	-0.19134500
	H	0.98020200	1.25338600	0.08644000
	H	-0.05420700	1.62501600	-1.08351100
	C	-1.76296300	-0.48796500	0.40905300
	H	-2.21830700	-1.48264300	0.28102100
	H	-1.58835400	-0.35567600	1.49232900
	O	-2.60874100	0.43467500	-0.13573800
	H	-2.21774400	1.28995600	0.02753600
**TS-3b**	C	-0.35755600	-0.47468600	-0.21835700
	H	-0.07136500	-0.17074600	-1.23601500
	O	0.43212500	-1.20569000	0.41896100
	O	1.71133200	-1.27183500	-0.18024700
	O	2.14947300	1.07527500	-0.29627300
	H	2.83825400	1.34954800	0.29726700
	H	2.04631600	-0.03722300	-0.23083900
	O	-0.10932900	1.33712100	0.40259300
	H	0.95968700	1.35710200	0.13245000
	H	-0.12732200	1.25031300	1.35304900
	C	-1.79753900	-0.54830600	0.13557500
	H	-2.17710800	-1.49976100	-0.27362000
	H	-1.89020600	-0.63025800	1.23732100
	O	-2.51140700	0.47081400	-0.41466200
	H	-2.02524800	1.27349900	-0.22589500
**TS-3c**	C	-0.37367100	-0.47633900	-0.22025300
	H	-0.11441400	-0.20917800	-1.25500100
	O	0.42711300	-1.19804300	0.41253000
	O	1.69013500	-1.28094200	-0.22087500
	O	-0.09760600	1.34262100	0.32654100
	H	-0.15059500	1.32567800	1.28018500
	H	0.98218300	1.34390000	0.11811900
	O	2.23454900	1.04938700	-0.06224700
	H	2.08706000	-0.04442500	-0.15321700
	H	2.58113100	1.37091500	-0.88596200
	C	-1.80581100	-0.53136500	0.16890700
	H	-2.19802400	-1.49669900	-0.19222900
	H	-1.87510600	-0.56679600	1.27463300
	O	-2.52877000	0.46776400	-0.40801100
	H	-2.03872000	1.27652800	-0.26196400
**TS-3d**	C	0.44637400	-0.52586800	0.28118700
	H	0.41994700	-0.58097400	1.38007900
	O	-0.47556200	-1.10190700	-0.34315000
	O	-1.63682000	-1.19862700	0.46890900
	O	0.04686300	1.27156700	0.22531500
	H	0.03334800	1.57145700	1.13049700
	H	-0.99057400	1.24530700	-0.07787900
	O	-2.24487400	0.94295200	-0.45149500
	H	-2.16969800	-0.04034700	-0.03443300
	H	-2.92559300	1.40567200	0.02216100

	C	1.76132600	-0.47427100	-0.41841200
	H	2.22391700	-1.46735600	-0.30672800
	H	1.57407100	-0.32897600	-1.49755100
	O	2.60717900	0.44764300	0.12976300
	H	2.21408800	1.30301400	-0.02753700
**C-1**	C	-0.09837700	-0.73915900	0.41414800
	H	0.11933300	-0.39829000	1.43629700
	C	-1.46635900	-0.76408100	-0.13596100
	O	-2.01011900	0.48124200	0.07354400
	O	0.85277200	-0.88527400	-0.35671100
	O	2.06223400	-0.53443400	0.11012400
	H	-1.34193100	1.13099200	-0.16075900
	H	-1.42578500	-1.06676300	-1.19802300
	H	-2.08980500	-1.49774500	0.39744300
	O	0.58901000	1.81502500	-0.13344900
	H	0.80617500	2.58927000	0.37246100
	H	1.36924600	1.24950800	-0.06461900
H_2O	O	0.00000000	0.00000000	0.11735200
	H	0.00000000	0.74824700	-0.46940700
	H	0.00000000	-0.74824700	-0.46940700

(d)optimized by MN15-L/MG3S

Species	Cartesian coordinates (Å)			
**1**	C	0.14181100	0.02442300	0.43070800
	H	0.30486000	0.71598000	1.26986900
	C	-1.19100100	-0.55568600	0.06746600
	O	-2.15282200	0.47123500	-0.10413700
	O	1.12914700	-0.25685500	-0.30758300
	O	2.33927500	0.26676700	-0.02176000
	H	-1.89568800	1.00376300	-0.86637100
	H	-1.07415500	-1.20164600	-0.82335600
	H	-1.56467000	-1.17969800	0.89865200
**TS-1a**	C	-0.26170000	0.61541300	-0.24965400
	H	-0.82490400	1.55622100	-0.33805400
	C	1.18322100	0.60514800	0.11069000
	O	1.78827800	-0.64842200	0.08391400
	O	-0.86724400	-0.48851000	-0.44542300
	O	-2.01962600	-0.10723400	0.40134500
	H	1.12251800	-1.31336100	0.30776000
	H	1.72811100	1.28967100	-0.56938000
	H	1.23389200	1.09743600	1.11476700
**TS-2a**	C	0.06838500	-0.17799500	0.68392700
	H	0.59895000	0.05533500	1.61879300
	C	-1.43849700	-0.16942100	0.59740200
	O	-1.87822000	-0.06426900	-0.73576800
	O	0.66493100	-0.90963400	-0.18685800
	O	2.05245800	-0.48175500	-0.20598100
	H	-1.48403600	-0.78996200	-1.23702100
	H	-1.81747600	-1.08252100	1.10520000
	H	-1.82111400	0.70390300	1.14750300

	O	0.56882200	1.53581200	-0.02510300
	H	0.05714900	1.55506300	-0.84821100
	H	1.42326700	1.00145300	-0.24455300
**TS-2b**	C	0.03836000	-0.27227800	0.65589100
	H	0.56658500	-0.07987700	1.60282100
	C	-1.46366600	-0.19267100	0.57121500
	O	-1.92382100	-0.07292700	-0.74170700
	O	0.65883200	-0.94035100	-0.24672800
	O	2.02732200	-0.46593100	-0.20614000
	H	-1.38824200	0.61063400	-1.16879400
	H	-1.88847400	-1.11913600	1.00256000
	H	-1.77923600	0.64722200	1.22576300
	O	0.58326400	1.45669500	-0.10134700
	H	0.81210800	2.09650700	0.58296400
	H	1.46431800	0.81446100	-0.24057000
**TS-3a**	C	-0.45127000	-0.49069600	-0.27320700
	H	-0.46663800	-0.61261700	-1.36846000
	O	0.49448400	-1.09988500	0.36419300
	O	1.67105200	-1.21017700	-0.49449700
	O	2.34139100	1.02150500	0.30413200
	H	2.57005200	0.99472500	1.23808800
	H	2.24598100	0.00765600	-0.02518400
	O	-0.03584200	1.23329300	-0.25686400
	H	1.00902500	1.23240700	0.06701800
	H	-0.01560700	1.54036000	-1.17339700
	C	-1.78087800	-0.45978700	0.46196400
	H	-2.21167800	-1.47387600	0.40404400
	H	-1.59116700	-0.22875300	1.52805700
	O	-2.69279600	0.42105400	-0.14449000
	H	-2.37338100	1.31667800	0.01749900
**TS-3b**	C	-0.35754600	-0.40471400	-0.20720800
	H	-0.07428700	-0.10319100	-1.22847600
	O	0.42490000	-1.22379600	0.41078300
	O	1.74739200	-1.28960400	-0.18644400
	O	2.19115800	1.10292700	-0.33645900
	H	2.93086400	1.38635400	0.20818600
	H	2.10093600	-0.00465000	-0.26548400
	O	-0.07441700	1.26811100	0.49332400
	H	0.98341100	1.35004000	0.18122800
	H	-0.05035100	1.08449600	1.44358200
	C	-1.83153200	-0.54079200	0.12136700
	H	-2.17676100	-1.49480700	-0.31409400
	H	-1.94714400	-0.61838100	1.22358400
	O	-2.59311100	0.48593600	-0.45075500
	H	-2.19957000	1.32458400	-0.17707900
**TS-3c**	C	-0.37860600	-0.40984700	-0.21125200
	H	-0.13387600	-0.16975200	-1.25810100
	O	0.42025100	-1.20606300	0.41400300
	O	1.71641100	-1.30632700	-0.23747900
	O	-0.06380100	1.28903700	0.37714500
	H	-0.09299200	1.19896800	1.34127000
	H	1.01219000	1.33632600	0.13922000

	O	2.30681500	1.06628500	-0.08423300
	H	2.15873700	-0.02048500	-0.16691400
	H	2.64200300	1.38404300	-0.92748300
	C	-1.84195700	-0.51820900	0.17345900
	H	-2.20396400	-1.49627900	-0.18803700
	H	-1.92326500	-0.52399900	1.28147300
	O	-2.61945800	0.47361700	-0.43957100
	H	-2.21720800	1.32711600	-0.23359100
**TS-3d**	C	0.45353100	-0.48934600	0.27963400
	H	0.48310300	-0.62159500	1.37401500
	O	-0.49191700	-1.10600600	-0.35343400
	O	-1.67091200	-1.19889800	0.50154200
	O	0.02795800	1.22144400	0.28441300
	H	-0.00094600	1.49602500	1.21043300
	H	-1.01702100	1.22685900	-0.06303700
	O	-2.27986000	0.96167500	-0.48761600
	H	-2.22996500	-0.02414500	-0.05616200
	H	-2.97994700	1.44526800	-0.03999600
	C	1.77655600	-0.45003500	-0.46756100
	H	2.21118900	-1.46297700	-0.42198900
	H	1.57557000	-0.20976200	-1.52915600
	O	2.69071900	0.42946900	0.13916000
	H	2.36960100	1.32513600	-0.01905700
**C-1**	C	-0.12302300	-0.75606500	0.41774000
	H	0.09255300	-0.43655600	1.44848500
	C	-1.50791400	-0.77048600	-0.15418900
	O	-2.07051600	0.50299600	0.08874200
	O	0.84882500	-0.91136300	-0.36684200
	O	2.10523200	-0.62689600	0.11439000
	H	-1.41920100	1.17089300	-0.17810100
	H	-1.45875900	-1.03507100	-1.22783800
	H	-2.14326600	-1.50809500	0.36562600
	O	0.65458600	1.89287300	-0.09381900
	H	0.96256200	2.77800600	0.11889000
	H	1.44671400	1.32924000	-0.04814300
H_2O	O	0.00000000	0.00000000	0.11835200
	H	0.00000000	0.75855000	-0.47340900
	H	0.00000000	-0.75855000	-0.47340900

Table S28. Absolute energies in hartrees

Species	Method	Total energies (a.u.)
**1**	W3X-L//Level-1	-304.3403743
	W2X//Level-1	-304.3366750
	W3X-L//Level-2	-304.3362886
	M11-L/MG3S	-304.1277669
	M06CR/MG3S	-303.9419443
	MN15-L/MG3S	-303.9441712
**TS-1a**	W3X-L//Level-1	-304.3232091
	W2X//Level-1	-304.3201488
	M11-L/MG3S	-304.1013292
	M06CR/MG3S	-303.9123079
	MN15-L/MG3S	-303.9190319
**TS-1b**	W2X//Level-1	-304.2869621
**TS-1c**	W2X//Level-1	-304.2908367
**TS-2a**	W3X-L//Level-1	-380.8395944
	W2X//Level-1	-380.8364340
	W2X//Level-2	-380.8359274
	M11-L/MG3S	-380.5689671
	M06CR/MG3S	-380.3364538
	MN15-L/MG3S	-380.3270358
**TS-2b**	W3X-L//Level-1	-380.8370269
	W2X//Level-1	-380.8338883
	W2X//Level-2	-380.8333777
	M11-L/MG3S	-380.5674539
	M06CR/MG3S	-380.3339715
	MN15-L/MG3S	-380.3236285
**TS-2c**	W2X//Level-2	-380.8252658
	M06CR/MG3S	-380.3283545
**TS-2d**	W2X//Level-2	-380.8249474
	M06CR/MG3S	-380.3283498
**TS-3a**	W3X-L//Level-2	-457.3554457
	M11-L/MG3S	-457.0220215
	M06CR/MG3S	-456.7493877
	MN15-L/MG3S	-456.7177337
	SC+W2X//Level-2	-457.3554457
	W2X//Level-2	-457.3520676
**TS-3b**	SC+W2X//Level-2	-457.3534589
	M11-L/MG3S	-457.0203106
	M06CR/MG3S	-456.7477197
	MN15-L/MG3S	-456.7155106
	W2X//Level-2	-457.3500808
**TS-3c**	W2X//Level-2	-457.3502608
	M11-L/MG3S	-457.0205108
	M06CR/MG3S	-456.7479355
	MN15-L/MG3S	-456.7156291
	SC+W2X//Level-2	-457.3536389
**TS-3d**	W2X//Level-2	-457.3501028
	M11-L/MG3S	-457.0199018
	M06CR/MG3S	-456.7477197

	MN15-L/MG3S	-456.7156956
	SC+W2X//Level-2	-457.3534809
**C-1**	W2X//Level-2	-380.8485727
	W2X//Level-1	-380.8491446
	W3X-L//Level-2	-380.8524192
	M11-L/MG3S	-380.581286
	M06CR/MG3S	-380.3520557
	MN15-L/MG3S	-380.3418584
**5**	W2X//Level-1	-380.9111131
	W2X//Level-2	-380.9106282
	M06CR/MG3S	-380.4063504
H_2O	W2X//Level-2	-76.49388013
	W3X-L//Level-1	-76.49443348
	W2X//Level-1	-76.49395334
	W3X-L//Level-2	-76.49388013
	M11-L/MG3S	-76.43603789
	M06CR/MG3S	-76.39107357
	MN15-L/MG3S	-76.37732625
**2**	W2X//Level-1	-304.3820878
**3**	W2X//Level-1	-228.5536404
**4**	W2X//Level-1	-304.3671915
	W2X//Level-2	-304.3666494
	M06CR/MG3S	-303.9813187
**C-2**	W2X//Level-2	-457.3619127
	M06CR/MG3S	-456.7616823
**C-3**	W2X//Level-2	-457.3647562
	M06CR/MG3S	-456.7654013
**C-4**	W2X//Level-2	-457.4178802
	M06CR/MG3S	-456.8135409
**C-5**	M06CR/MG3S	-456.7954969
$(H_2O)_2$	M06CR/MG3S	-151.473326
	W3X-L//Level-2	-152.9966627
	W2X//Level-2	-152.9956821
**TS-3e**	M06CR/MG3S	-456.7301378
**TS-3f**	M06CR//MG3S	-456.7294799
**TS-3g**	M06CR/MG3S	-456.7284922
**6**	M06CR//MG3S	-228.9104976
**7**	M06CR//MG3S	-304.1371266

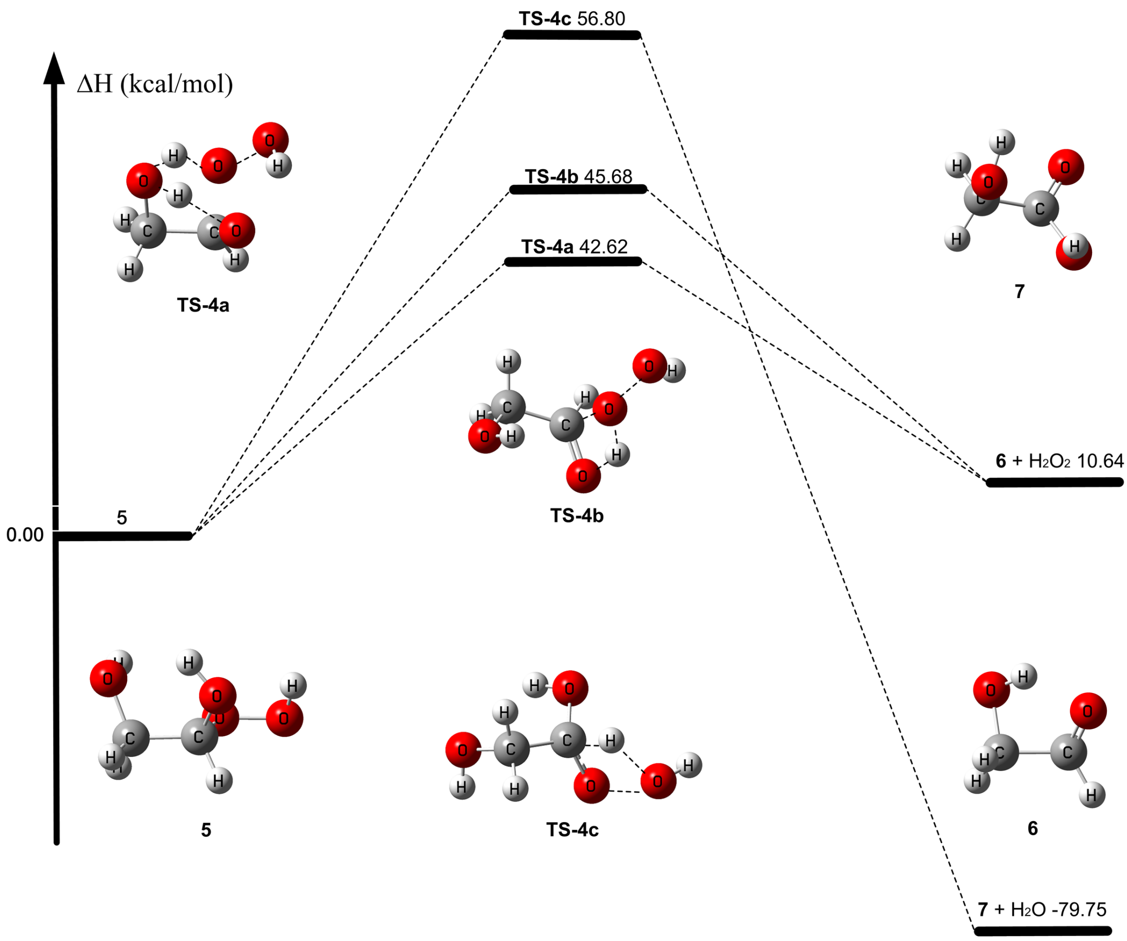

Figure S1. Enthalpy (at 0 K) profile of the decay of the product **5** of **1**+ H_2O calculated by M06CR/MG3S (in kcal/mol) with generic scale factors .

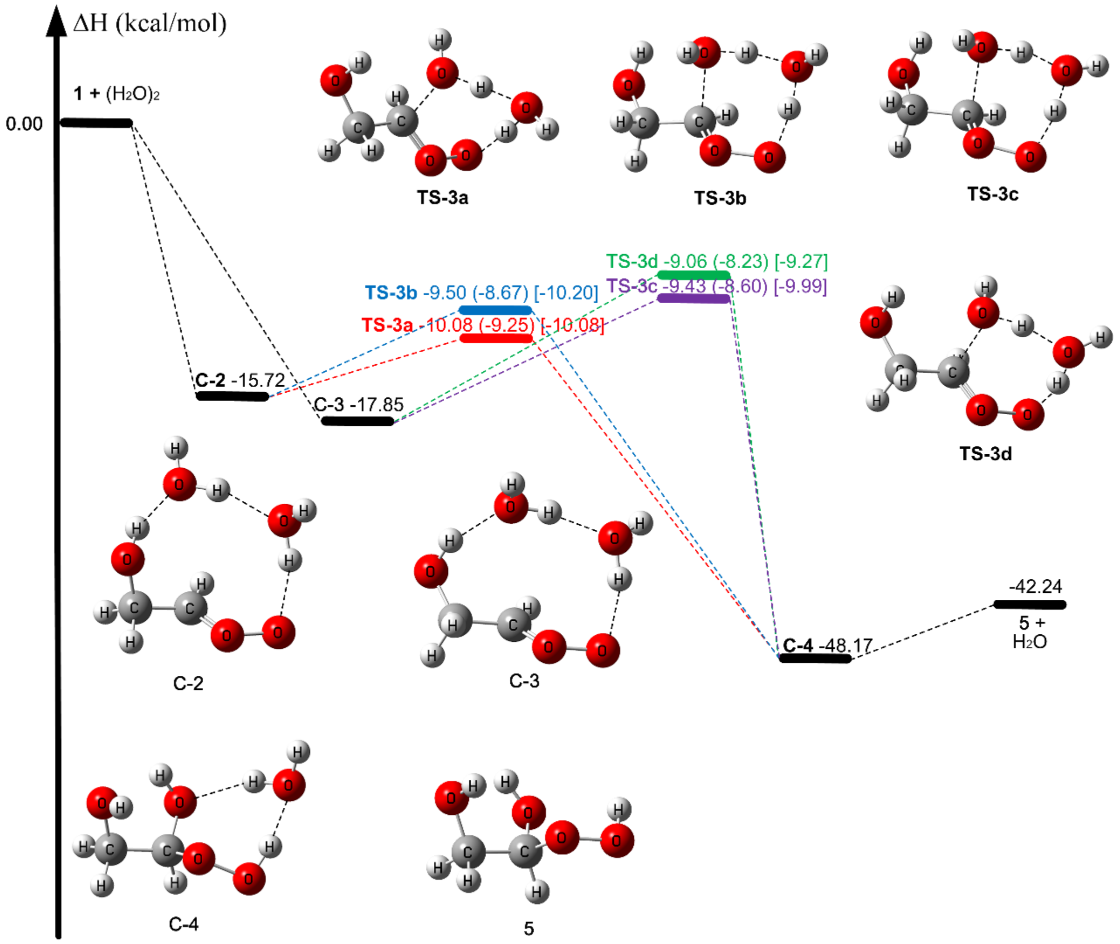

Figure S2. Relative enthalpy (0 K) of R4.1 with respect to **1** and $(H_2O)_2$. All values were calculated by W2X//Level-2 except that the results in parentheses are obtained by W3X-L//Level-2 for **TS-3a** and by SC+W2X//Level-2 for **TS-3b**, **TS-3c**, and **TS-3d**. SC+W2X//Level-2 includes the semiempirical correction (0.83 kcal/mol for transition states and 0 kcal/mol for reactants), which equals $\Delta H_0^\ddagger$ result for **TS-3a** relative to **1** + $(H_2O)_2$ using W3X-L//Level-2 minus $\Delta H_0^\ddagger$ for **TS-3a** relative to **1** + $(H_2O)_2$ using W2X//Level-2. By this definition, SC+W2X//Level-2 is the same as to W3X-L//Level-2 for the $\Delta H_0^\ddagger$ of **TS-3a** relative to **1** + $(H_2O)_2$ with generic scale factors. The values in brackets are obtained using SC+W2X//Level-2 with reaction-specific scale factors.

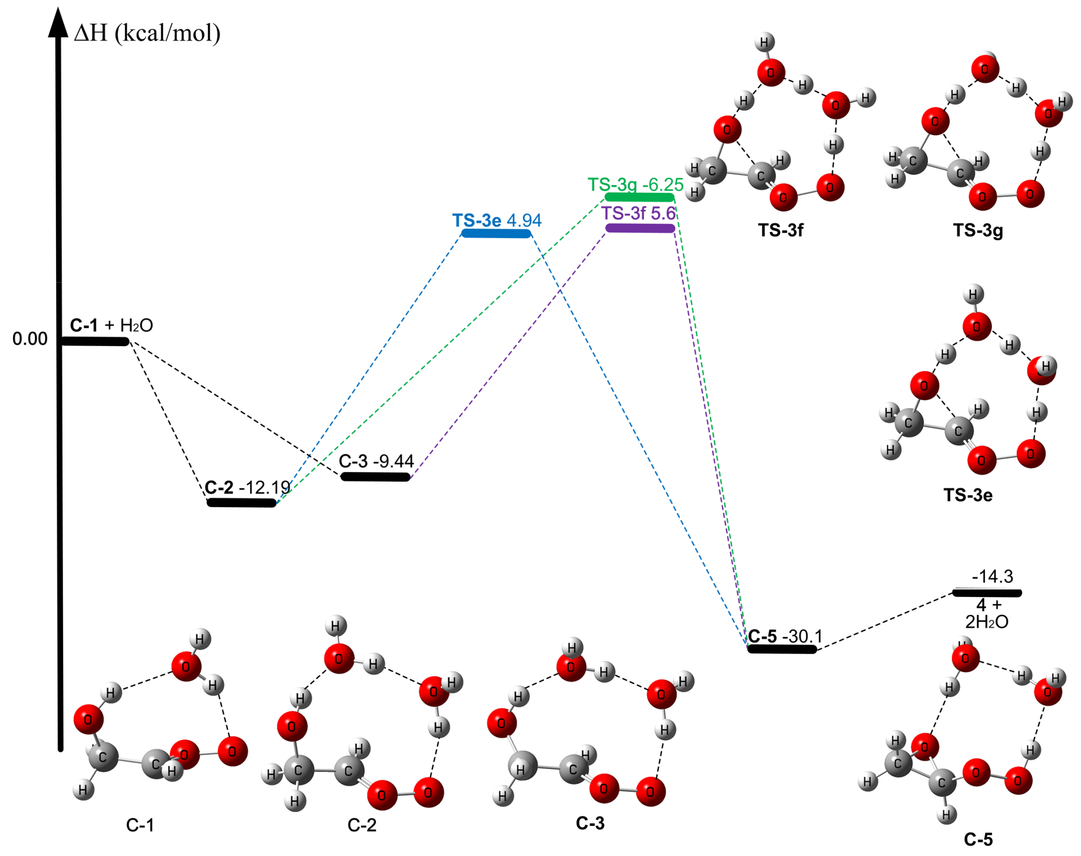

Figure S3. Relative enthalpy profile at 0 K (in kcal/mol) for R3.2 calculated by M06CR/MG3S with generic scale factors .

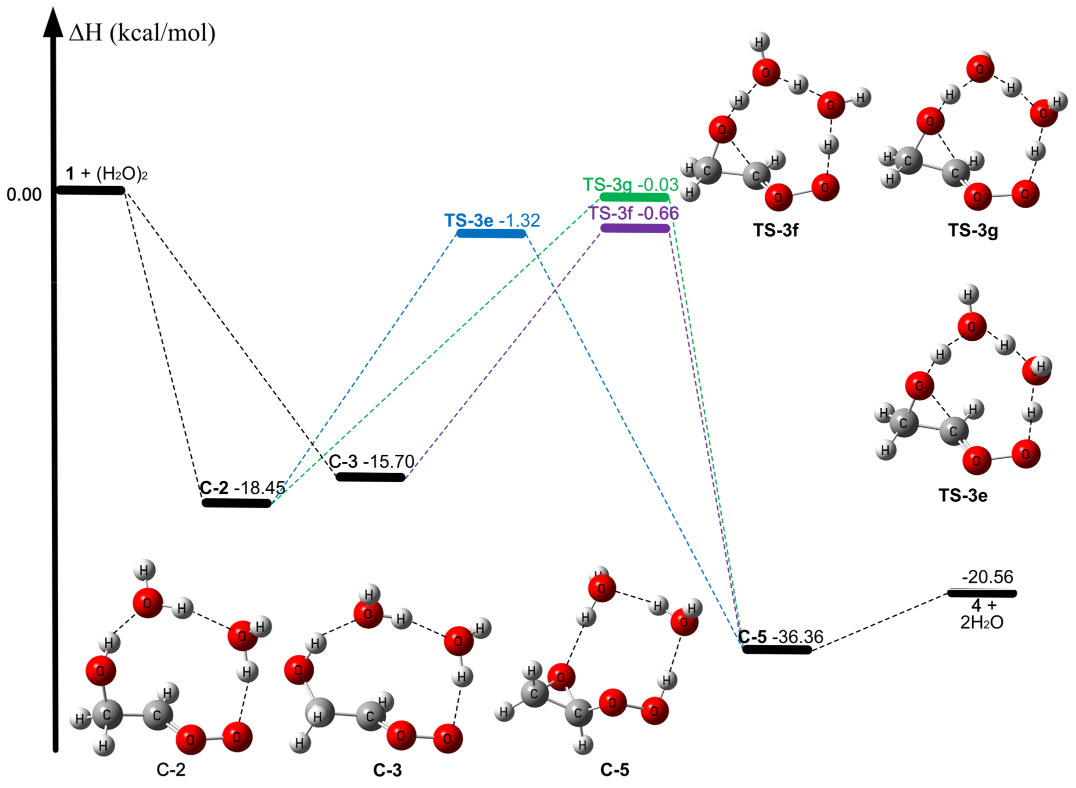

Figure S4. Relative enthalpy profile at 0 K (in kcal/mol) for calculated by M06CR/MG3S with generic scale factors .

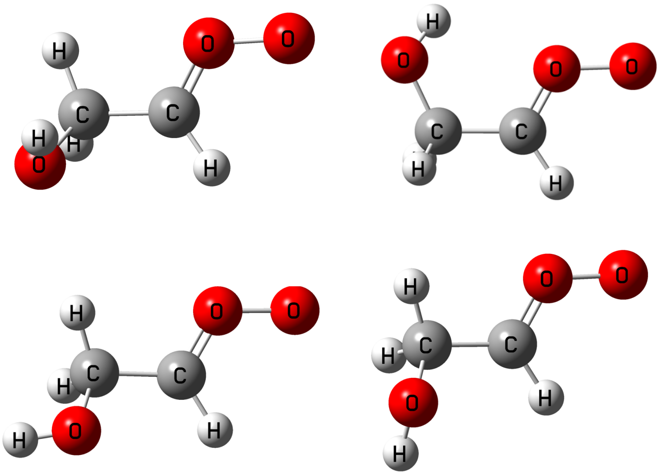

Figure S5. The distinguishable structures for conformers of **1.** These structures are used in F_{act}^{MS} factor calculations for **1**.

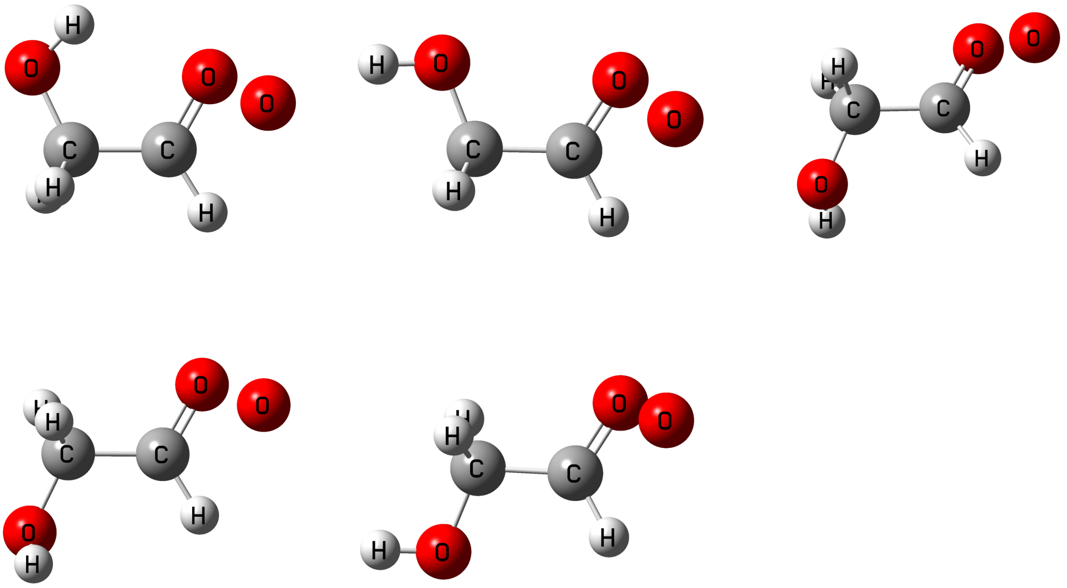

Figure S6. The distinguishable structures for conformers of **TS-1a.** These structures are used in F_{act}^{MS} factor calculations for **TS-1a**.

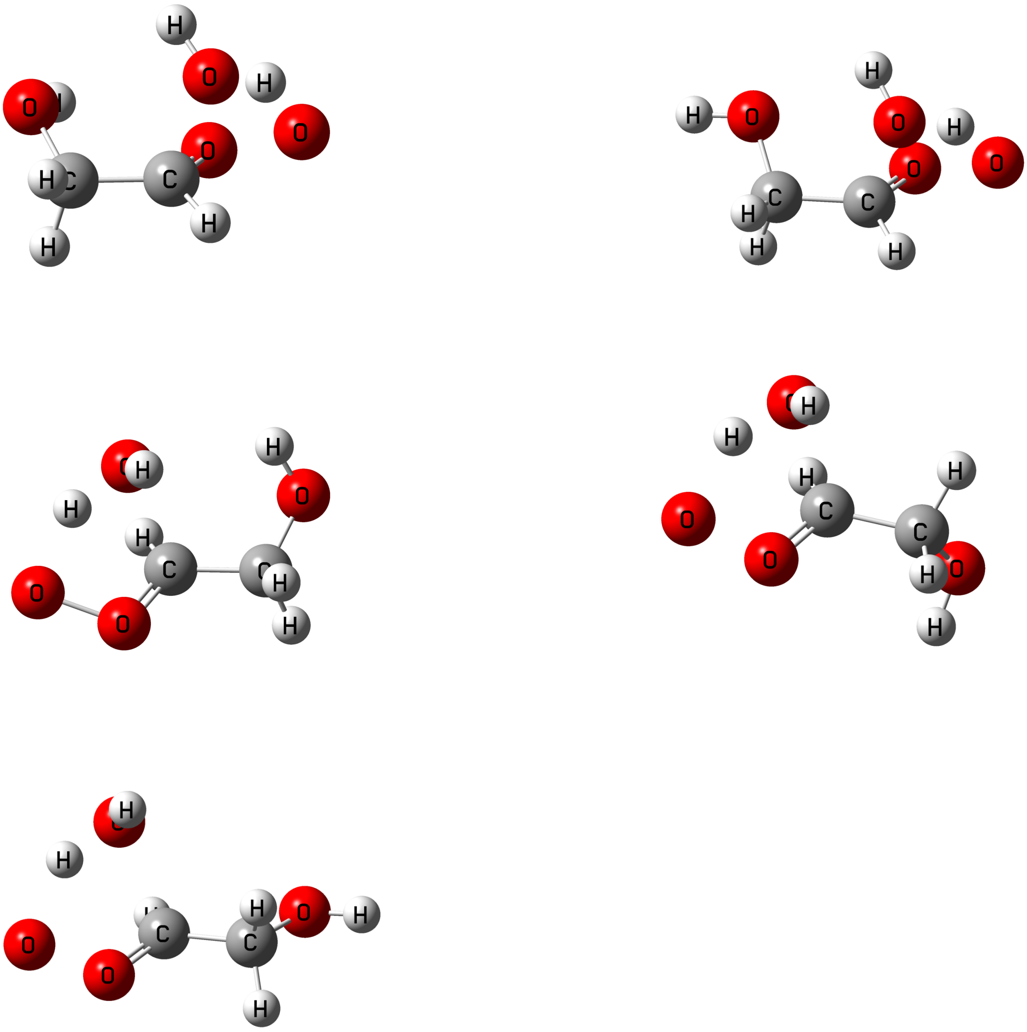

Figure S7. The distinguishable structures for conformers of **TS-2a.** These structures are used in F_{act}^{MS} factor calculations for **TS-2a**.

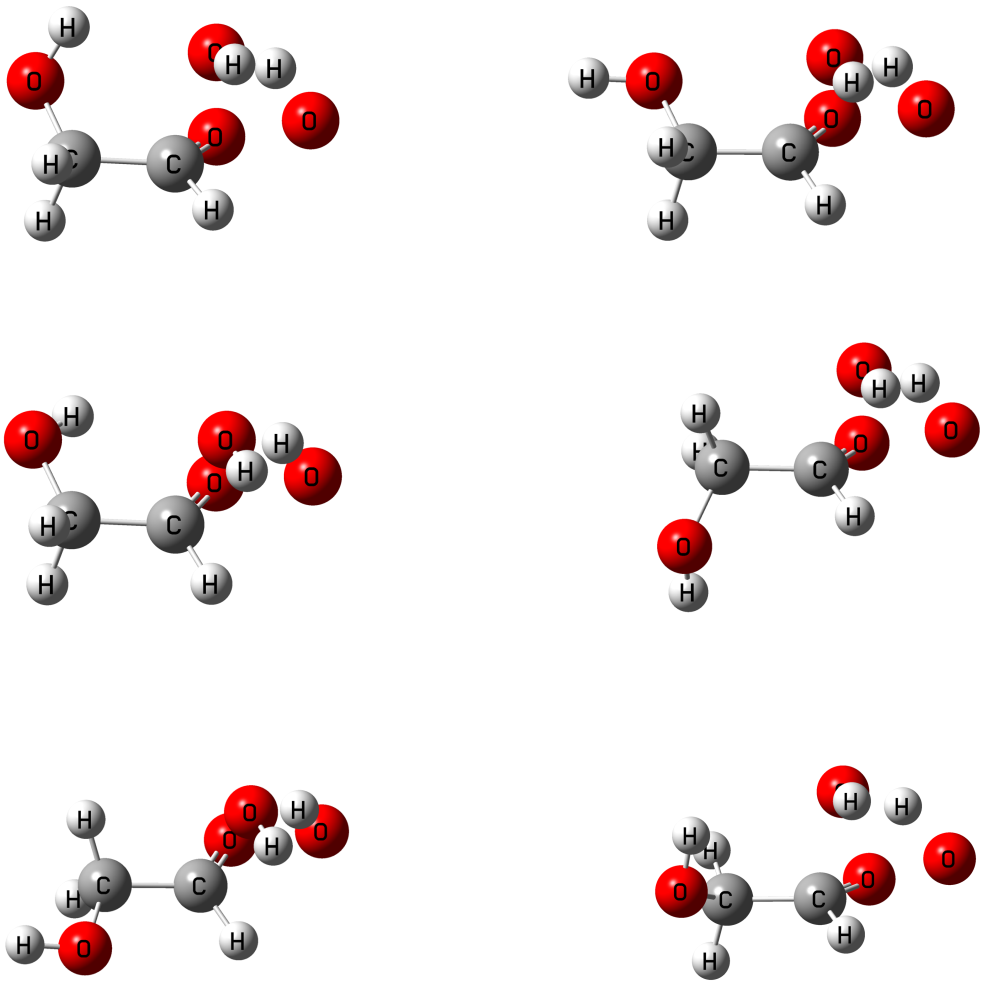

Figure S8. The distinguishable structures for conformers of **TS-2b.** These structures are used in F_{act}^{MS} factor calculations for **TS-2b**.

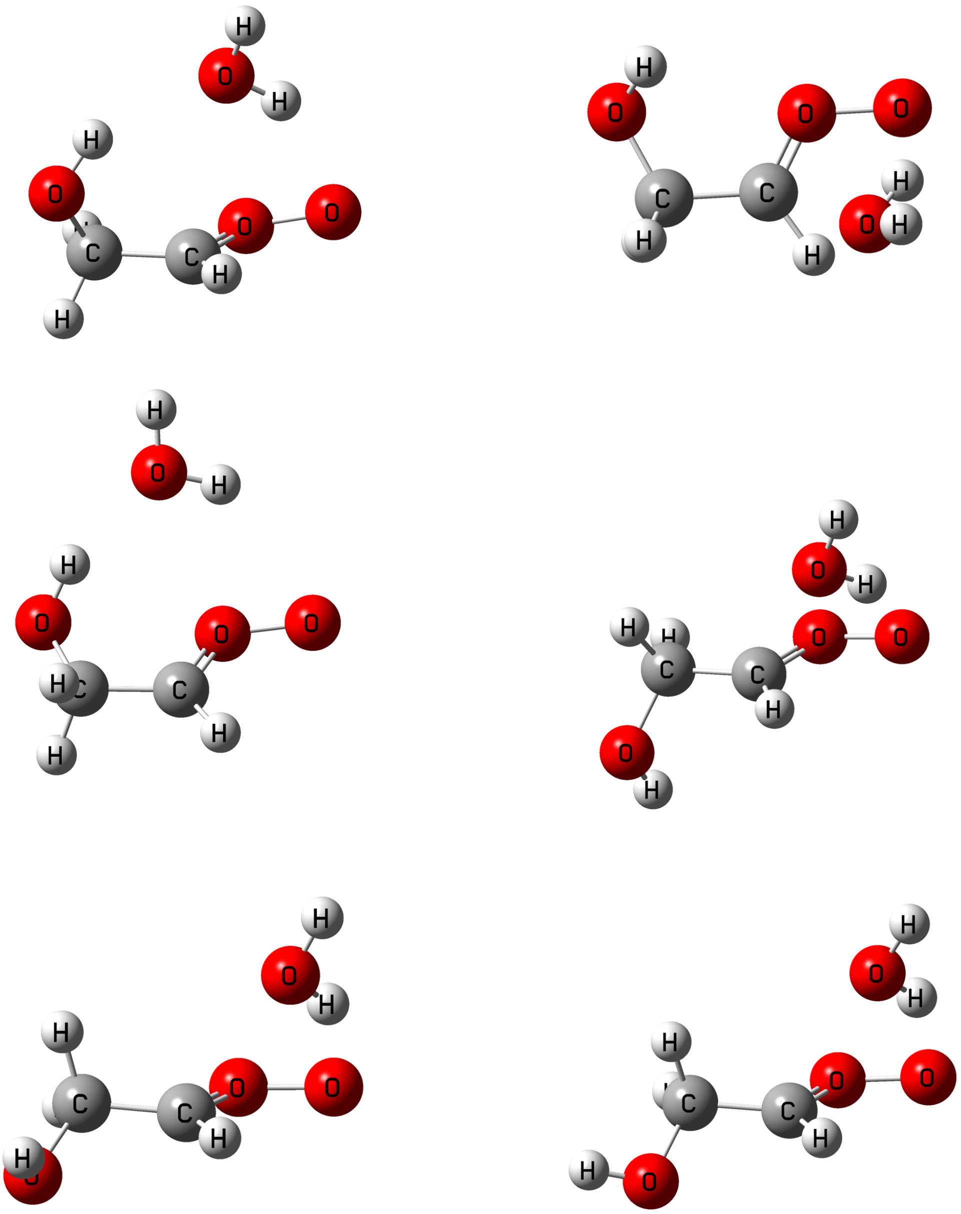

Figure S9. The distinguishable structures for conformers of **C-1.** These structures are used in F_{act}^{MS} factor calculations for **C-1**.

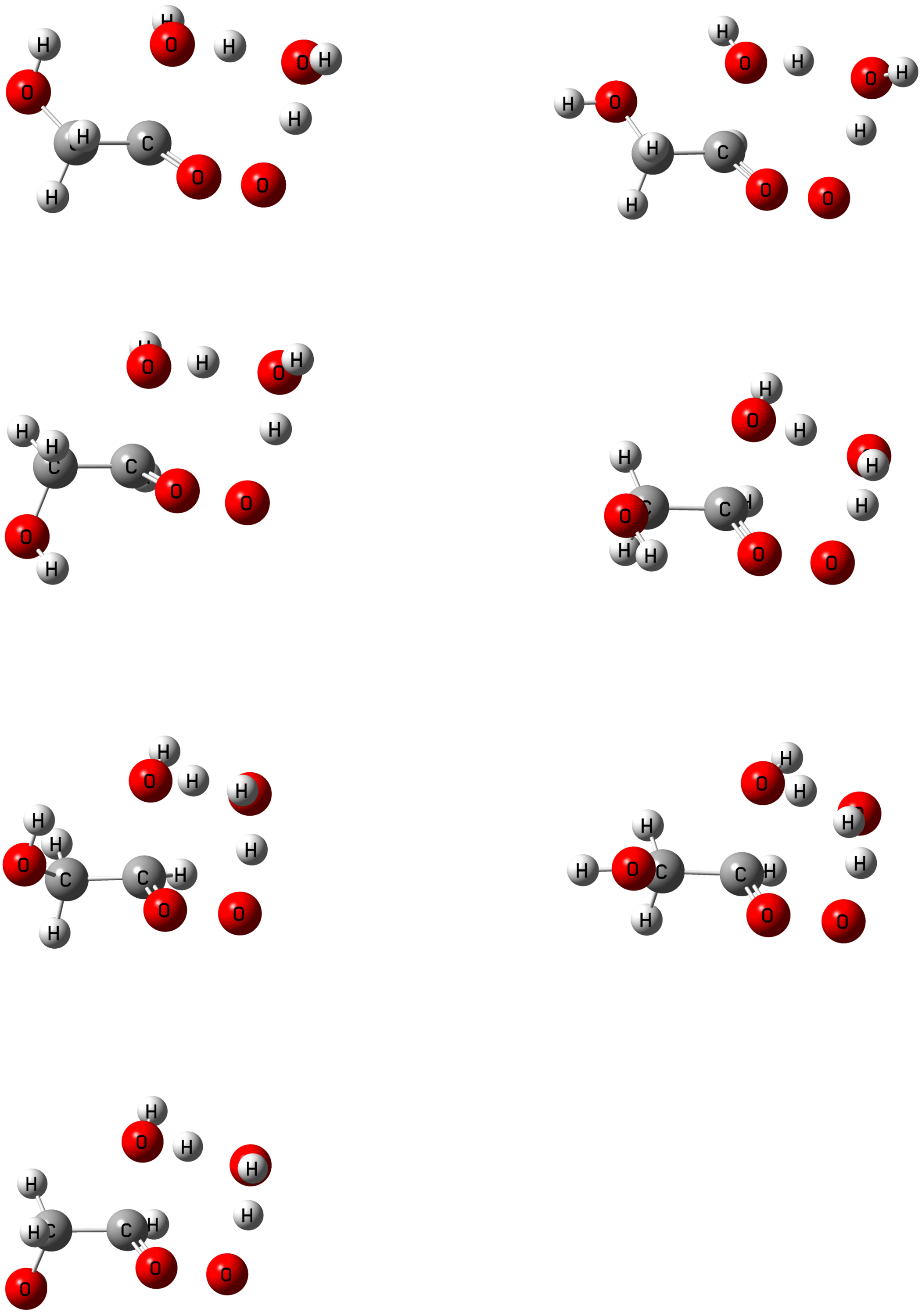

Figure S10. The distinguishable structures for conformers of **TS-3a.** These structures are used in F_{act}^{MS} factor calculations for **TS-3a**.

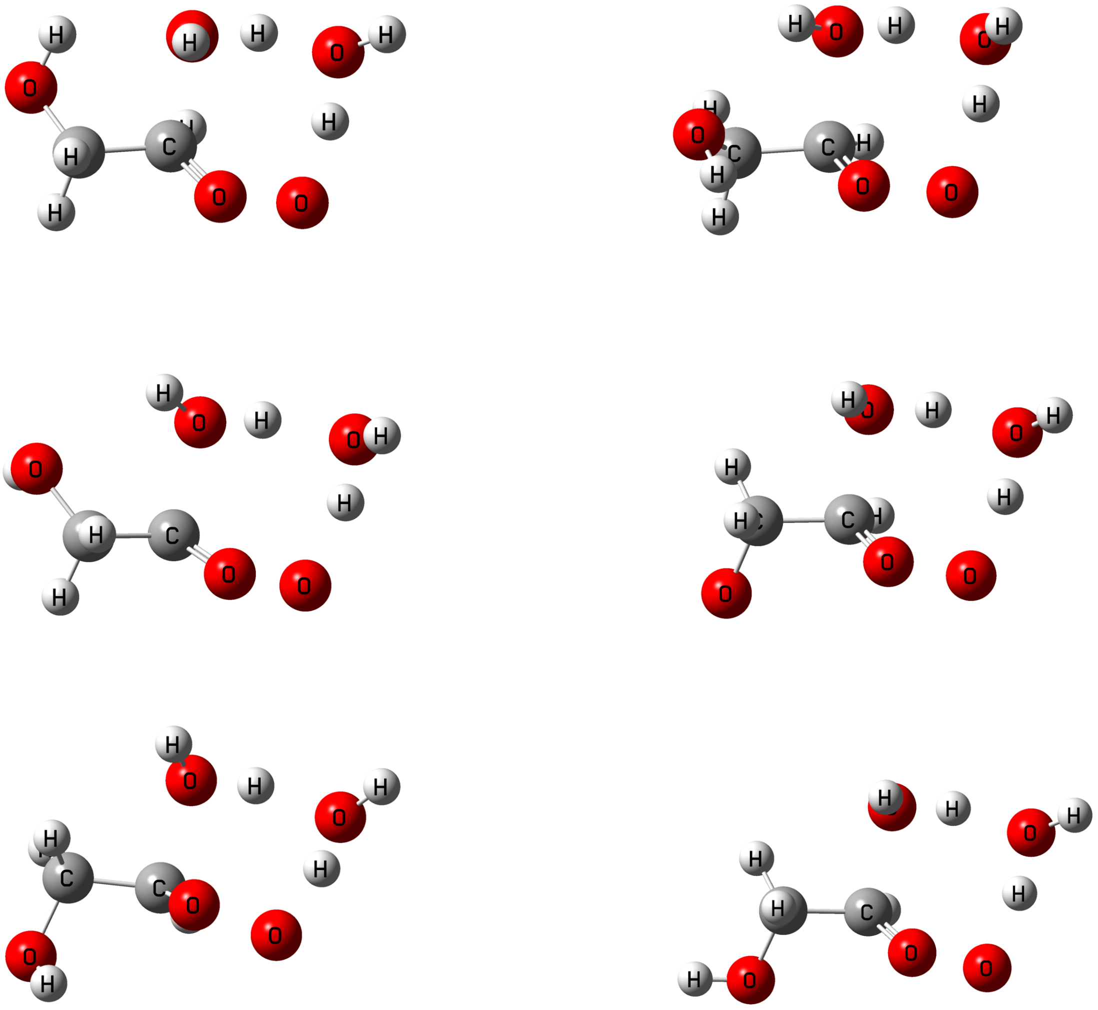

Figure S11. The distinguishable structures for conformers of **TS-3b.** These structures are used in F_{act}^{MS} factor calculations for **TS-3b**.

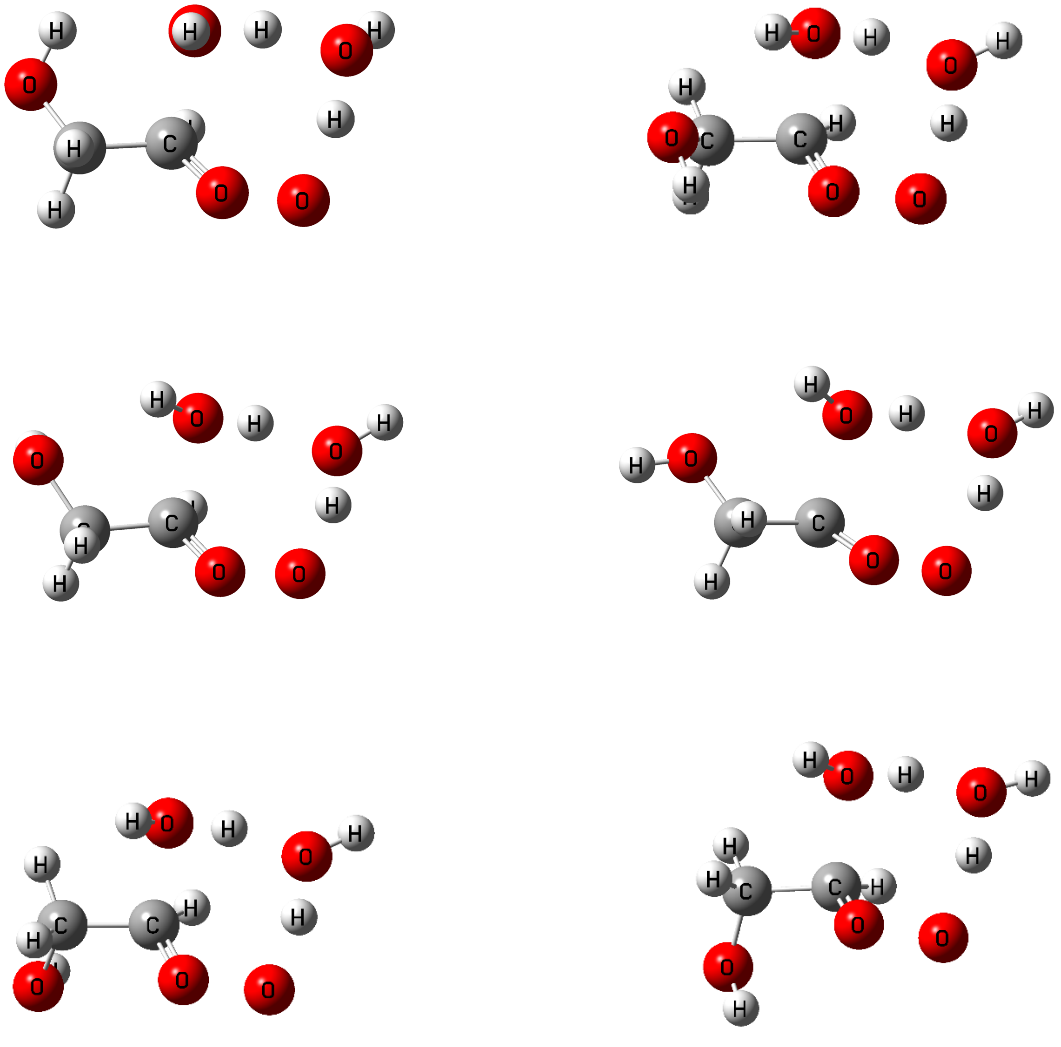

Figure S12. The distinguishable structures for conformers of **TS-3c.** These structures are used in F_{act}^{MS} factor calculations for **TS-3c**.

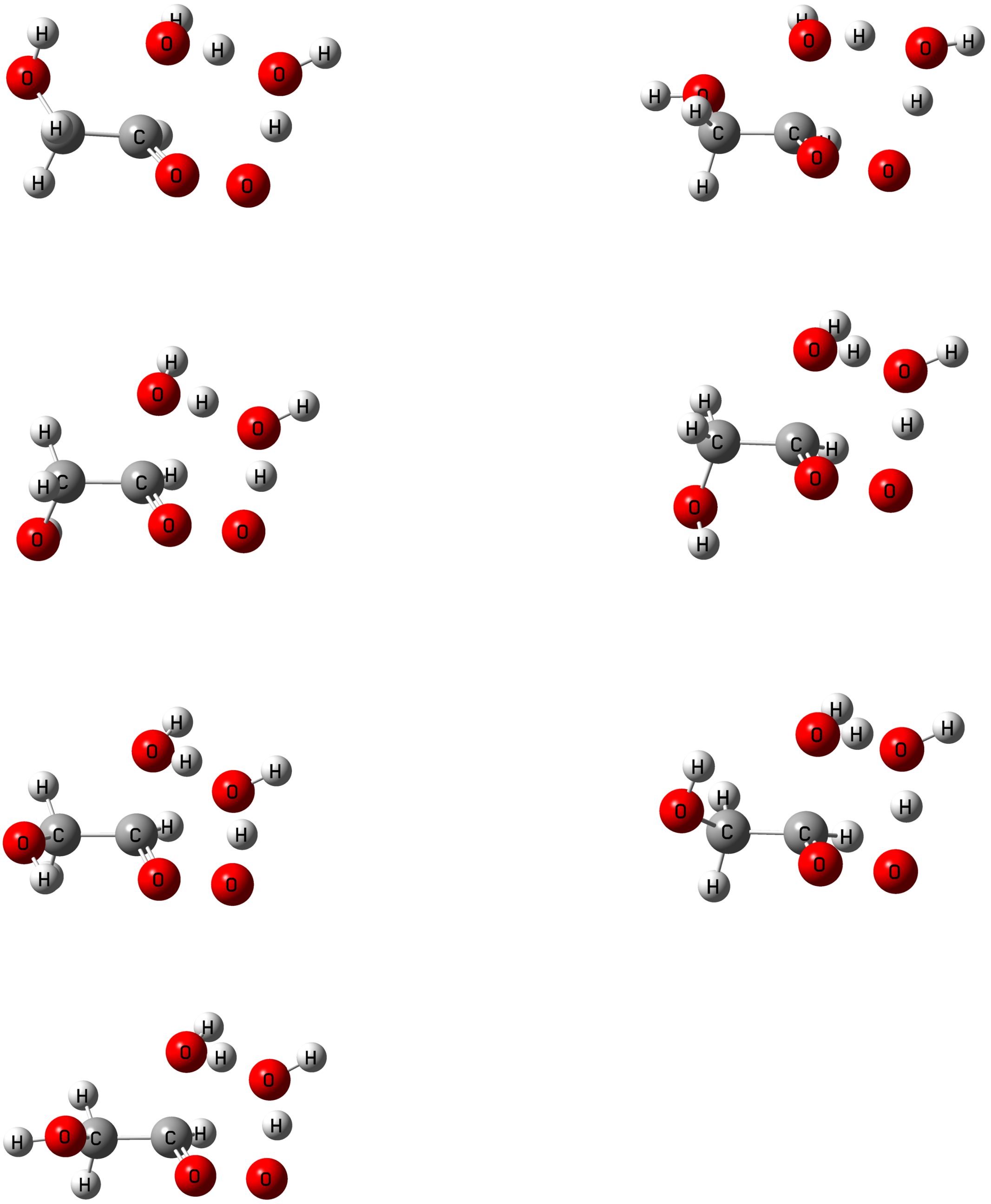

Figure S13. The distinguishable structures for conformers of **TS-3d.** These structures are used in F_{act}^{MS} factor calculations for **TS-3d**.

References

[1] Y. Georgievskii and S. J. Klippenstein, "Variable reaction coordinate transition state theory: Analytic results and application to the C_2H_3+H→C_2H_4 reaction," *The Journal of Chemical Physics*, vol. 118, no. 12, pp. 5442-5455, 2003.

[2] J. Zheng, S. Zhang, and D. G. Truhlar, "Density Functional Study of Methyl Radical Association Kinetics," *The Journal of Physical Chemistry A*, vol. 112, no. 46, pp. 11509-11513, 2008.

[3] B. Long, Y. Wang, Y. Xia, X. He, J. L. Bao, and D. G. Truhlar, "Atmospheric Kinetics: Bimolecular Reactions of Carbonyl Oxide by a Triple-Level Strategy," *Journal of the American Chemical Society*, vol. 143, no. 22, pp. 8402-8413, 2021.
